# The ecology of an adaptive radiation of three‐spined stickleback from North Uist, Scotland

**DOI:** 10.1111/mec.13746

**Published:** 2016-08-08

**Authors:** Isabel S. Magalhaes, Daniele D'Agostino, Paul A. Hohenlohe, Andrew D. C. MacColl

**Affiliations:** ^1^ School of Life SciencesUniversity of NottinghamUniversity ParkNottinghamNG7 2RDUK; ^2^ Institute for Bioinformatics and Evolutionary StudiesDepartment of Biological SciencesUniversity of IdahoMoscowID83844USA

**Keywords:** environment, genomics, phenotype, three‐spined stickleback

## Abstract

There has been a large focus on the genetics of traits involved in adaptation, but knowledge of the environmental variables leading to adaptive changes is surprisingly poor. Combined use of environmental data with morphological and genomic data should allow us to understand the extent to which patterns of phenotypic and genetic diversity within a species can be explained by the structure of the environment. Here, we analyse the variation of populations of three‐spined stickleback from 27 freshwater lakes on North Uist, Scotland, that vary greatly in their environment, to understand how environmental and genetic constraints contribute to phenotypic divergence. We collected 35 individuals per population and 30 abiotic and biotic environmental parameters to characterize variation across lakes and analyse phenotype–environment associations. Additionally, we used RAD sequencing to estimate the genetic relationships among a subset of these populations. We found a large amount of phenotypic variation among populations, most prominently in armour and spine traits. Despite large variation in the abiotic environment, namely in ion composition, depth and dissolved organic Carbon, more phenotypic variation was explained by the biotic variables (presence of predators and density of predator and competitors), than by associated abiotic variables. Genetic structure among populations was partly geographic, with closer populations being more similar. Altogether, our results suggest that differences in body shape among stickleback populations are the result of both canalized genetic and plastic responses to environmental factors, which shape fish morphology in a predictable direction regardless of their genetic starting point.

## Introduction

Understanding the origins of phenotypic diversity and speciation has challenged evolutionary biologists and ecologists for over a century. Darwin (1859) speculated that although variation within species is often correlated with abiotic variation, it is more likely that differences between populations are driven by interactions with the biotic environment (e.g. predators, competitors and parasites), because of its inherent dynamism. Adaptive radiations provide dramatic examples of rapid change in phenotypes and local adaptation of populations in contrasting environments, as illustrated by the rapid and repeated evolution of North American postglacial fishes, African cichlids, Caribbean *Anolis* lizards and Darwin's finches that have captivated evolutionary biologists for decades (Losos *et al*. 1998; Schluter 2000; Kocher 2004). These examples provide tangible evidence of phenotypic divergence and the operation of mechanisms of selection. Explanations for particular radiations often involve a combination of several factors: intrinsic, nonecological explanations (such as genetic drift), as well as extrinsic biotic and abiotic conditions. However, the integration of more than two of these factors in one study is rare. Most of the literature on adaptive radiations in animals focuses on morphological traits and how the capability of individuals to exploit different resources and niches has led to reproductive isolation (Schluter 1996; Rundell & Price 2009; Yoder *et al*. 2010). Not that abiotic factors have been unimportant in the diversification of animals; in fact, most radiations mentioned above have been shaped by external conditions (e.g. fluctuations in lake depth in Africa, glaciation in North America, El Niño events in the Galapagos, island size in the Caribbean). It is simply that empirical studies of diversification in animals have focused on biotic interactions such as resource competition (Pfennig *et al*. 2007; Tyerman *et al*. 2008) and predation (Langerhans *et al*. 2004; Nosil & Crespi 2006), as primary agents driving divergence and structuring natural communities. Additionally, studies that have examined environmental correlates of evolution have commonly focussed on the relationship between a single environmental cause (*e.g*. predation, competition or temperature) and the evolution of one or a small number of traits (Schluter 1994; Vamosi 2002; Marchinko 2009; but see Bourgeois *et al*. 1994). Nonetheless, the understanding of the origin of diversity can largely benefit not only from the integration of information on several factors, but also from the recognition of the multivariate nature of the environment and the phenotypes. Here, we used an integrated approach combining phenotypic, environmental and genomic data to analyse the extrinsic factors driving a radiation of three‐spined sticklebacks (*Gasterosteus aculeatus*). Three‐spined sticklebacks are a classic example of recent adaptive radiations where a lot of focus has been on the divergence of a few adaptive traits, such as armour and spines and the genetic changes underlying them (Cresko *et al*. 2004, Colosimo *et al*. 2005; Barrett *et al*. 2008, Chan *et al*. 2010), while knowledge of the environmental drivers of divergence has lagged behind (but see Reimchen 1980, 1992, 1994; Nosil & Reimchen 2005).

We analysed the phenotypic and genomic variation and divergence in the adaptive radiation of three‐spined stickleback on North Uist, Scotland, to try to understand the relative importance of abiotic and biotic factors in driving divergence among populations. Marine sticklebacks have colonized many lakes on this island since the ice sheet covering it melted in the past 16 000 years (Ballantyne 2010). Large variation in pH and calcium among lakes has been reported and linked to the evolution of body size or armour in stickleback in these lakes (Giles 1983; Spence *et al*. 2013; MacColl & Aucott 2014), but an integrated approach of phenotypes, genotypes and environment has so far not been attempted. In this study, we quantified phenotypic variation across 27 lakes, and looked at potential correlations between 10 external morphological traits (body shape and armour) within and across populations. Finding significant correlations among traits within populations would suggest that groups of traits are inherited jointly and evolve together, potentially constraining evolution, while significant correlations across but not within populations would suggest phenotypic variation is the result of environmentally driven selection varying across populations. We also used 30 abiotic and biotic environmental parameters to characterize the environmental variation, and restriction site‐associated DNA sequencing (RADseq; Etter *et al*. 2011) to reveal the genomewide genetic structure and divergence among populations. We then used multivariate statistical tools to explore the role of the multivariate environment and of genomic variation in shaping patterns of phenotypic variation. We first tested the hypothesis that phenotypic variation across the island is associated with variation in the biotic and in the abiotic environments. This allowed us to test the abiotic and biotic environmental axes of phenotypic variation separately and to find how much of the phenotypic variation is explained by the abiotic and biotic environments separately and combined. We then tested the hypothesis that phenotypic variation is associated with genomewide genetic variation among populations. Finding a significant association between the two would suggest that ecological processes driving the observed phenotypic variation among populations are also affecting both neutral and selected genetic variation, a process called isolation by adaptation (IBA; Nosil *et al*. 2009), although it could also be the result of the spatial autocorrelation of environments (coancestry). Finally, we analysed how both environmental and genomic variation are associated with individual phenotypic axes of variation, testing the hypothesis that both environment and genetics constraint variation in phenotypic traits.

## Material and methods

### Stickleback collection

**Figure 1:**
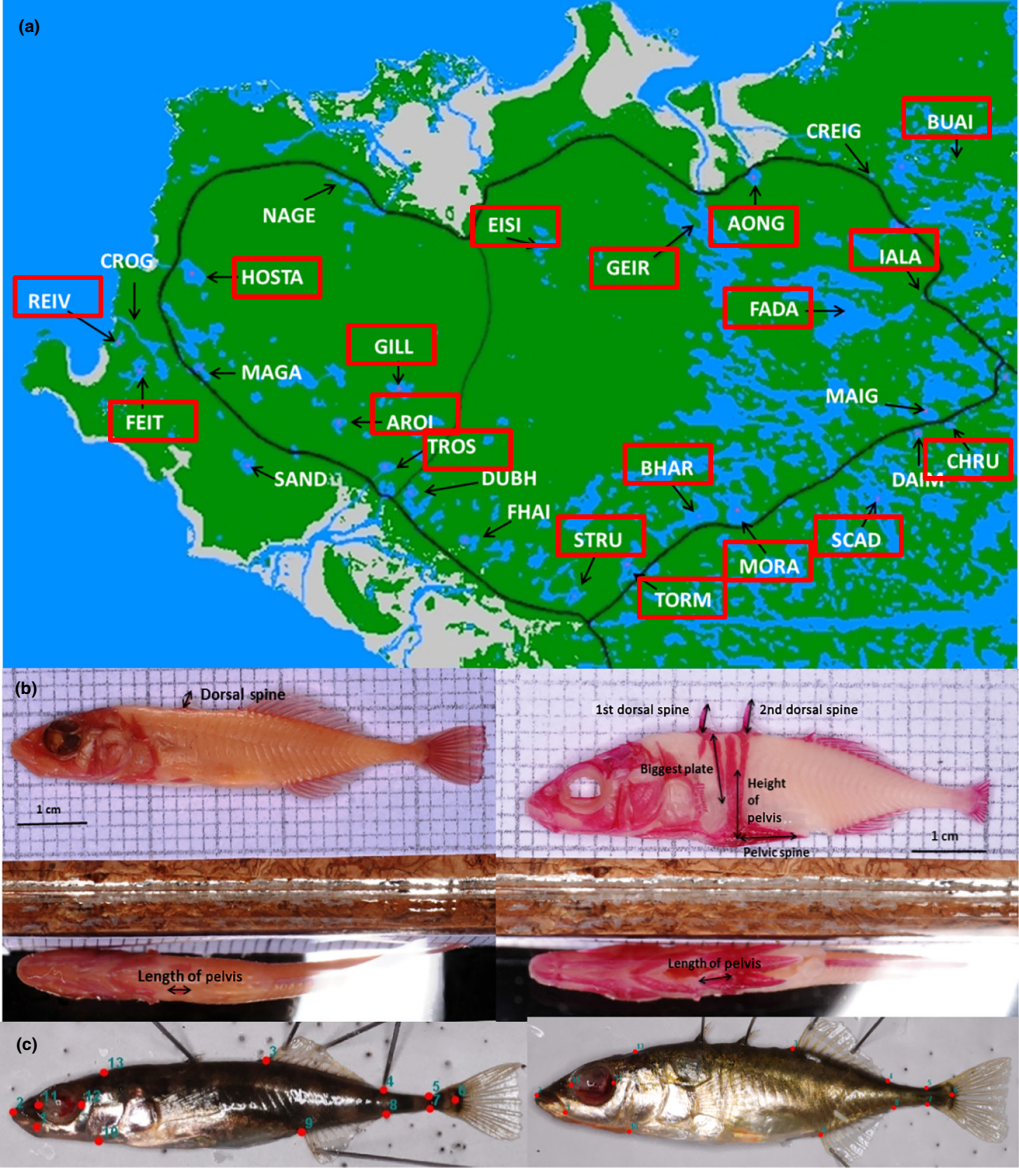
(a) Map of the island of North Uist with lakes sampled. In black text are abbreviations of the names of the sampling sites. Framed names are names of populations for which we did RADseq analyses. Pictures of the extreme phenotypes that were observed on different lakes with (b) measurements of dorsal and pelvic spines, armour plates and pelvic armour, (c) landmarks used in the analyses of body shape (13 landmarks).

We sampled 27 freshwater lakes between April and June 2013 (Fig. 1a, Table 1). Fish were collected using unbaited minnow traps, which were left in each lake for approximately 24 h. Typically, 20–30 unbaited minnow traps (Gee traps, Dynamic Aqua, Vancouver, Canada) were set in water approximately 0.3–3 m deep, along a 100–400 m stretch of shoreline. This usually comprised a substantial proportion of the perimeter of a loch (5–25%). Once the traps were collected, we counted numbers of individuals of three‐spined stickleback. These numbers were then used to estimate species’ densities (number of fish per unit effort). Density data collected in this way show significant correlations between years (ADCM, unpublished data). At the same time, we also registered the presence and counted the numbers of nine‐spined sticklebacks *Pungitius pungitius,* the only species of comparable adult size that occurs in these lochs and a potential competitor of three‐spined sticklebacks (Hart 2003). Approximately 35 three‐spined stickleback per population were selected for processing. The fish were haphazardly selected with individuals of all sizes, sex and breeding condition collected. After humanely killing the fish by overdose with the anaesthetic MS222, we measured their standard length (SL) and weighed them. We then placed each fish lying flat on their right side in a standardized position and took a lateral photograph of the left side for geometric‐morphometric analyses of body shape. Fish were later stained with alizarin red to enable measurement of external bony structures using standard protocols. All morphological measurements and analyses were done using the left side of the fish.

**Table 1:** Names of lakes sampled, abbreviations of their names, location and numbers of three‐spined sticklebacks processed from each lake (N) and number of individuals used in the genomic analyses (Ngen)

Lake Name	Abbreviation	Location	N	Ngen
Aonghais	AONG	57°39″N; 7°16″W	34	18
Mhic a'Roin	AROI	57°35″N; 7°25″W	34	19
a'Bharpa	BHAR	57°34″N; 7°17″W	33	18
na Buaile	BUAI	57°38″N; 7°11″W	35	17
Chadha Ruaidh	CHRU	57°36″N; 7°12″W	32	17
na Creige	CREI	57°39″N; 7°14″W	34	NA
an Daimh	DAIM	57°35″N; 7°12″W	33	20
Dubhasaraidh	DUBH	57°35″N; 7°24″W	32	NA
Eisiadar	EISI	57°38″N; 7°21″W	34	17
Fada	FADA	57°36″N; 7°12″W	35	15
nam Feithean	FEIT	57°36″N; 7°30″W	33	NA
Fhaing Buidhe	FHAI	57°34″N; 7°23″W	35	NA
nan Geireann	GEIR	57°38″N; 7°17″W	34	17
Mhic Gille‐bhride	GILL	57°36″N; 7°24″W	31	17
Grogary	GROG	57°37″N; 7°30″W	34	NA
Hosta	HOST	57°37″N; 7°29″W	34	19
Iala	IALA	57°37″N, 7°12″W	35	18
nam Magarlan	MAGA	57°36″N; 7°29″W	35	NA
Maighdein	MAIG	57°35″N; 7°12″W	35	NA
na Moracha	MORA	57°34″N; 7°16″W	30	21
ne Gearrachun	NAGE	57°39″N; 7°25″W	35	NA
na Reival	REIV	57°37″N; 7°31″W	35	19
Sandary	SAND	57°35″N; 7°28″W	35	NA
Scadavay	SCAD	57°35″N; 7°14″W	35	17
nan Strùban	STRU	57°34″N; 7°21″W	35	16
Tormasad	TORM	57°33″N; 7°19″W	30	19
Trosavat	TROS	57°35″N; 7°25″W	33	20

### Biotic environmental data collection

We concentrated on competition, predation and parasitism as biotic factors which might affect stickleback evolution. The lakes on North Uist contain rather simple, species poor communities. Nine‐spined stickleback is the only other small fish species present, and the only obvious competitor for three‐spined stickleback (MacColl *et al*. 2013). The commonest potential predator is the brown trout (*Salmo trutta*). Atlantic salmon (*Salmo salar*) and charr (*Salvelinus alpinus*) are present in some lochs, but the former are only present seasonally and do not usually eat in freshwater, while the latter are rare. Avian predators including terns (*Sterna* spp.) and divers (*Gavia* spp.) occur on most lochs but at very low density (with the possible exception of one loch, ‘REIV’). Insect predators of stickleback (especially diving beetle (*Dytiscus* spp.) and dragonfly (Odonata) larvae) also occur, but are rare except in lochs with no trout (‘BUAI’, ‘CHRU’, ‘IALA’ and ‘REIV’). Stickleback on North Uist are infected by numerous species of parasites (de Roij & MacColl 2012). We chose to examine two (*Gyrodactylus arcuatus* and *Schistocephalus solidus*) that are relatively common and widespread, with the potential to cause selection (MacColl 2009).

We obtained the relative densities of *P. pungitius* by estimating the proportion of fish in the minnow traps that belonged to this species. The presence of brown trout was obtained from previous work (MacColl *et al*. 2013; MacColl & Aucott 2014). For a subset of the lakes studied, we also obtained trout average length and catch rate (‘TCR’) per population (trout caught per angler per hour) from published estimates (MacColl & Aucott 2014). These data originate from fishing competitions held in fairly standardized conditions by the local angling club over a 50‐year period (1956–2006). The number of *Gyrodactylus arcuatus* and *Schistochephalus solidus* per fish was counted during dissections (see Table S1, Supporting information for average and standard deviations of parasite numbers per population and biotic variables per lake).

### Abiotic environmental data collection

Abiotic data for each lake were mostly collected on the day the minnow traps were set. Average depth (‘Av. depth’) and maximum depth (‘Max. depth’) of each lake were either estimated using a Plastimo Echotest II hand‐held depth sounder to record the depth at several points (20–180) along each lake, or taken from existing literature (Murray & Pullar 1910). The area of each loch was estimated from Google Earth using Web‐based planimeter software (http://www.freemaptools.com/area-calculator.htm) (MacColl *et al*. 2013). Conductivity, pH and salinity were measured using a calibrated pH meter (Multi 340i, WTW, Weilheim, Germany). The concentrations of metallic cation concentrations and of dissolved organic carbon (sodium (‘Na’), potassium (‘K’), calcium (‘Ca’) magnesium (‘Mg’), copper (‘Cu’), manganese (‘Mn’), zinc (‘Zn’), cadmium (‘Cd’), lead (‘Pb’), sulphates and dissolved organic carbon (DOC)), chlorophyll A (‘ChlA’) were obtained by collecting in the field two filtered water samples (one acidified with 2% nitric acid, one frozen) from each of the lakes. These samples were then analysed at the Division of Agriculture & Environmental Science at the University of Nottingham for metallic cation concentrations by inductively coupled plasma mass spectrometry (ICP‐MS), anions using a Dionex DX500 ion chromatograph with an IonPac AS14A (4 × 250 mm) and dissolved organic carbon (DOC) using a Shimadzu TOC‐Vcph with an ASI‐V autosampler.

Littoral substrate at 0.5 m depth (percentage of mud, soft peat, hard peat, sand, gravel and rock in 1 m^2^ area) was estimated by visual inspection and probing with a pole every 50 m around the margin of each lake, or for 500 m either side of where the traps were set.

Light transmission spectra in the 179–882 nm range were determined using an OceanOptics USB2000 + UV + VIS‐ES spectrometer with a fibre cable and spectrasuite software. Measurements were taken in the shade. We then used the spectra to estimate the mean transmission rate, maximum transmission rate and the wavelength (nm) with the maximum transmission rate. We also estimated the relative transmission of long wavelengths, which can be quantified by the ‘orange ratio’, or the integral of 400–550 nm transmission divided by the integral of 550–700 nm transmission (Endler & Houde 1995). This orange ratio is larger when longer wavelengths are transmitted more than short wavelengths, that is the environment is more red‐shifted.

### Data analyses

##### Axes of phenotypic variation – body shape, armour and spine analyses

To quantify body shape variation across individuals and lakes, we digitized 13 homologous landmarks (Fig. 1b) using the tps software package (Rohlf & Marcus 1993; Rohlf 2010a,b). Landmark coordinates for 908 individuals were exported and analysed using morphoj 1.03 (Klingenberg 2011). Briefly, we first performed a Procrustes superimposition (Dryden & Mardia 1998) to extract shape coordinates for further analyses (Dryden & Mardia 1998; Rohlf 1999; Zelditch *et al*. 2004), and then performed a size correction to account for any allometric effects (Loy *et al*. 1996; Klingenberg 2010) using a multivariate regression pooled by lochs of individual Procrustes coordinates against the logarithm of centroid size and tested its significance using a permutation test against the null hypothesis of independence (10 000 iterations). Last, we performed a principal component analysis (PCA) on the regression residuals to extract the PCs of body shape variation (bodyPCs). We retained the first 2 bodyPCs (the only axes that explained more than 10% of the total variance) and used them in further analyses.

Using photographs of the left side of alizarin‐stained individual fish, we measured the following body armour and spines traits: number of armour plates (‘n. plates’), length of 1st dorsal spine (‘DS1’) and 2nd dorsal spine (‘DS2’), length of biggest armour plate (‘BAP’), pelvic spine length (‘PS’), length of the horizontal process of the pelvis (‘LP’) and height of the ascending process of the pelvis (‘HP’) (Fig. 1b) (see Table S3, Supporting information for averages and standard deviations of all traits and SL per population). Because all traits, apart from number of plates, were significantly correlated with SL (results not shown), we size corrected the data by regressing each trait against SL (all individuals from all lakes pooled) and used the residuals in further analyses.

To estimate and visualize the major axes of phenotypic variation across individuals and lakes, we combined the first two body PCs, the number of plates and the measurements on armour and spines in one principal component analysis (PCA). All variables were scaled and centred prior to the PCA.

Finally, we tested for collinearity of the traits analysed by performing Pearson's correlations between individual measurements for each population separately and with all individuals from all populations pooled. We adjusted the statistical significance in the above tests for multiple comparisons using a false discovery rate (FDR) of 0.05 (Benjamini & Hochberg 1995). We analysed all data in r v3.0.0 (http://www.R-project.org.).

##### Axes of environmental variation – abiotic environmental data analyses

In order to visualize axes of abiotic environmental variance across lakes, we combined all 26 abiotic environmental variables in one PCA to obtain the major axes of environmental variation. To establish which environmental variables are correlated with each other, we performed Pearson's correlations for all environmental variables. We adjusted the statistical significance in the above tests for multiple comparisons using a false discovery rate (FDR) of 0.05 (Benjamini & Hochberg 1995).

##### RAD library preparation and sequence analyses

Genomic DNA was purified from 17 to 21 individuals from each of 18 of the populations, chosen to represent a widely distributed subset of the most environmentally and phenotypically variable lakes. Extracted genomic DNA was normalized to a concentration of 25 ng/μL in 96‐well plates and processed into RAD libraries according to Etter *et al*. (2011), using the restriction enzyme SbfI‐HF (NEB). Samples were individually ligated to adaptors with 6‐bp in‐line barcodes and multiplexed in libraries of 192. Libraries were sequenced in two lanes on an Illumina HiSeq sequencer at the University of Oregon, producing 100‐bp single‐end reads.

Raw sequence reads were demultiplexed using Stacks – 1.35 (Catchen *et al*. 2011, 2013). Only those reads with the correct barcode and an unambiguous RAD site were retained. At this stage, we excluded from further analyses 21 individuals that had less than 500 000 reads, and retained a total of 324 individuals. The retained reads were then aligned to the three‐spined stickleback reference genome (version BROADs1, Ensembl release 82) using GSnap (Wu & Watanabe 2005; Wu & Nacu 2010). Reference mapping with GSnap took sequence quality information into account, allowed for up to five mismatches and up to 2 indels between each read and the reference sequence and ignored reads which mapped against more than a single position in the genome. Aligned reads were analysed in Stacks, which derived each locus from overlapping GSnap alignments to produce a consensus sequence (see Table S5, Supporting information for detailed summary statistics of number of number of reads retained and aligned). SNPs were determined and genotypes called using a maximum‐likelihood statistical model implemented in Stacks (Catchen *et al*. 2013). Population genetics statistics (major allele frequency, percentage polymorphic loci and nucleotide diversity) were calculated for each SNP using the populations program in Stacks. The following filters were applied: only SNPs that were present in at least 80% of all individuals combined and in at least one individual in all 18 populations were included in the analyses; SNPs with a minor allele frequency below 0.05 were removed; we retained one random SNP per RAD locus to avoid as much as possible linked loci; and we removed the sex chromosome (chromosome 19) from the analyses. The same program was used to calculate within‐population fixation indices (F_IS_; Wright 1951), to measure genetic differentiation among populations from different lakes (*F*_ST_, Hohenlohe *et al*. 2010) and to generate output files for further analyses. To visualize the genetic distances among individuals and populations, we computed an unrooted Neighbour‐Joining tree using the r package Adegenet (Jombart 2008). The same package was used to perform a PCA and plot population means for the first two major axes of genetic variation. To test for population subdivision and bottlenecks, we estimated effective populations sizes (Ne) using the molecular linkage disequilibrium (LD) method and the (Waples & Do 2008) and the heterozygote‐excess (HE) method (Zhdanova & Pudovkin 2008) as implemented in neestimator V2 (Do *et al*. 2014).

We analysed population genetic structure and assigned individuals to populations using a variational Bayesian framework for posterior inference from large SNP genotype data as implemented in faststructure (Pritchard *et al*. 2000; Raj *et al*. 2014). The faststructure algorithm was run for 1–18 populations (*K*) using the default value of conversion criterion of 10e‐6 and the simple prior. We then run the algorithm installed in the same program to find the appropriate number of model components that explain structure in the data set.

Finally, we tested for isolation by distance (IBD) by estimating the correlation between the matrix of genetic distance between pairs of populations (*F*_ST_/(1−*F*_ST_)) and the matrix of geographic distances, and tested the significance of the correlation using a Mantel test with 10 000 permutations as implemented in arlequin (Excoffier *et al*. 2005).

##### Phenotype–genome–environment associations

To analyse associations between the phenotype, the genome and environment, the phenotypic data were related to environmental and genomic variables using canonical redundancy analyses (RDA) (Rao 1964). This constrained ordination technique consists of a multivariate multiple regression (MMR) relating the matrix of response variables (phenotypic traits) to a corresponding matrix of explanatory variables, followed by a PCA on the fitted values resulting from the MMR model to extract the RDA axes. The RDA axes are therefore the linear combinations of the explanatory variables that best explain the variation of the response matrix (see Borcard *et al*. 2011 for a detailed explanation of the RDA method). A stepwise backward selection procedure by exact Akaike information criteria (AIC) was used to select variables that best explained variation in the response data with the significance of the best models assessed by permutations (Anderson & Legendre 1999). The *R* ^2^ of each model was adjusted for multiple regressions (Ezekiel 1930). RDAs and backward selection of variables were performed using the ‘vegan’ package (Oksanen *et al*. 2013) in r. Our response matrix consisted of population averages of the 10 phenotypic variables (SL, bodyPC1, bodyPC2, n. plates, DS1, DS2, BAP, PS, LP and HP).

The first part of the analyses focused on the relationship between the whole phenotype and the environment and involved relating the response matrix to two environmental explanatory matrices in two separate RDAs: the first matrix consisted of 10 PCs of abiotic environmental variation and the second of 4 biotic variables (population averages of *Gyrodactylus arcuatus* and *Schistochephalus solidus,* presence and percentage of *P. pungitius* and trout presence). We then combined the best explanatory abiotic and biotic variables in one explanatory matrix to assess the percentage of the phenotypic variation explained by the two sets of explanatory variables combined. Additionally, we repeated the analyses using a reduced data set of 23 populations so we could include quantitative information on trout catch rate (TCR) (rather than just presence) in the biotic explanatory variables.

The second part of the analyses focused on the relationship between the phenotype and the genetic data and involved performing an RDA relating the phenotypic response matrix to a genetic explanatory matrix consisting of population averages of the first 10 PCs of genetic variation. This analysis was performed using the data set of 18 populations for which genetic information was available.

Finally, as different traits might be associated with different aspects of the environment and the genome, we analysed the relationship between separate phenotypic traits, the genome and the environment. First, we performed multiple linear regressions with the first four phenotypic PCs as response variables and the environmental and genetic variables used in the previous analyses as explanatory variables. A stepwise backward selection procedure by exact AIC was used to select variables that best explained variation in the response data with the significance of the best models assessed by permutations (Anderson & Legendre 1999) as implemented in the r package ‘ape’ (Paradis *et al*. 2004). Then, we used the same method to analyse the relationship between the genomic variation and the environment and performed multiple linear regressions with the first 10 genomic PCs as response variables and the environmental variables as response variables.

## Results

### Axes of phenotypic variation

The two bodyPCs that were retained for further analyses explained a total of 50.7% of the variance. PC1 of body shape (‘bodyPC1’) distinguished pronounced shape changes associated with the position of the beginning of the dorsal and anal fins and length of the caudal peduncle, and PC2 (‘bodyPC2’) with the position of the end of the dorsal fin, position of the anal fin and lower jaw length (Fig. 1, see Table S4, Supporting information for loadings and % variance explained by each PC).

**Figure 2:**
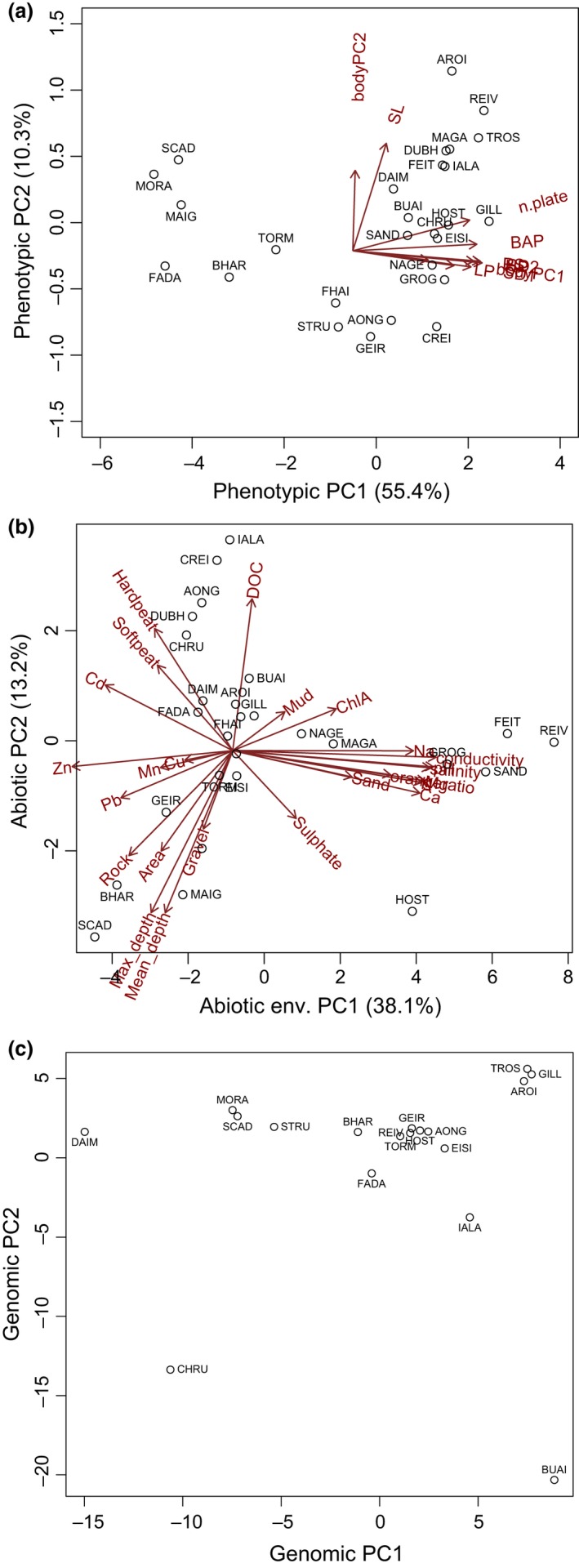
PCA analyses of (a) 10 phenotypic variables, (b) 25 abiotic environmental variables (variables follow Material & Methods) and (c) 9 135 genetic markers. Arrows and text at the end of them indicate the loadings of each variable on each axis, and the circles indicates the relative average phenotype (a) and relative environment (b) of each lake. Site names follow Fig. 1.

The PCA combining all phenotypic (shape and size plus armour) traits revealed that the first four PCs of phenotypic variation explained 83% of the variance among individuals (see Table S6, Supporting information for details on loadings and percentage explained by each of the 10 PC axes). In this analysis, all armour and spine measurements and counts had heavy positive loadings on PC1 (‘armour PC’), which explained 55% of the variation. Standard length had a heavy positive loading on PC2 (‘SL PC’), which accounted for 10% of the variation. We found a gradient of populations along PC1, ranging from populations of individuals with no armour plates or dorsal and ventral spines to populations of individuals that have all spines and some armour plates. The second axis showed a gradient starting with populations with the shortest individuals and ending with populations with the longest individuals (Fig. 2a). PC3 of phenotypic variation explained 10% of the variation was mostly represented by bodyPC2 and therefore distinguished among individuals with a deeper body, larger head and short caudal peduncle and individuals with a more slender body, smaller head and more elongated peduncle. PC4 (7.7%of phenotypic variation) separated individuals based on bodyPC1, which distinguished pronounced shape changes associated the body depth and length of the posterior body and caudal peduncle.

Phenotypic variation had a modular structure, with certain groups of traits being consistently positively correlated with each other, but not with some other traits. When individuals from all populations were pooled together all armour traits were highly positively correlated (Pearson's correlation product (*r*) >0.5, *P*‐value ≤0.05 after adjustment for multiple testing) (Table S7, Supporting information). Other variables such as SL and bodyPC1 and bodyPC2 were also significantly correlated with some armour traits, although not as strongly. Within populations, the number of traits correlated varied greatly: on average, 10 correlations were significant within populations, ranging between 1 (DAIM) and 24 (EISI). Of 405 correlations among all armour traits (except number of plates), 175 (43%) were significant.

### Biotic and Abiotic environmental variation across lakes

The lakes sampled varied in presence and abundance of brown trout and nine‐spined stickleback. In total, 3 lakes had no trout and no nine‐spined sticklebacks in them, 13 had both species present, 9 had only trout, and 1 (REIV) had only nine‐spined stickleback in them, in addition to three‐spined stickleback. Density of brown trout (as measured by TCRs) ranged between 0.053 ± 0.01 (SE) (DUBH) and 2.29 ± 0.42 (MORA) fish per angler per hour. In lakes where 9‐spined stickleback was presented, they made up between 6% and 64% of the fish caught in the minnow traps. The prevalence of parasites found on fish was also quite variable: lakes ranged from no three‐spined stickleback infected with *Schistochephalus* spp. (CREI, DUBH, NAGE) to an average of 5 parasites per fish (BHAR), and infections with *Gyrodactylus arcuatus* ranged from 0 (CHRU, DAIM) to 6 (GILL) parasites per fish.

The first PC of abiotic environment (‘envPC1’) explained 38.1% of the variance among lakes and discriminated between alkaline lakes with high levels of alkali metals and clear water, such as REIV, SANN, GROG, FEIT and HOST at one end and acidic lakes with low conductivity such as MAIG, SCAD and BHAR at the other (Fig. 2b) (see Table S8, Supporting information for details on loadings and percentage explained by each of the envPC axes). PC2 (‘envPC2’) explained 13.2% of the variance among lakes and discriminated between very shallow lakes with high DOC such as IALA and CREIG and relatively deep oligotrophic lakes such as MAIG, SCAD and BHAR (Fig. 2b). The loadings on these two PCs reflected the fact that some of the environmental variables analysed formed groups of highly correlated variables (Pearson's correlation product (*r*) >0.5, *P*‐value < 0.05 after adjustment for multiple testing, Table S9, Supporting information): pH, salinity, conductivity, Zn, Ca, Na, Mg, K and orange ratio formed one group, and average and maximum depth and DOC formed a second group of highly correlated variables with high loadings on the first (PC1) and second (PC2) major axes of environmental variation, respectively.

### Genetic variation and divergence

populations retained 9135 SNPs across all data (Table 2). Note that this set of loci is a representative sample of genetic variation across the genome, presumably including both neutral loci and loci under various forms of selection. Two populations stood out for their much higher numbers of private alleles (BUAI: 31 and CHRU: 25). These two populations also showed reduced levels of genetic diversity as indicated by the lowest numbers of polymorphic sites (BUAI: 2390, CHRU: 1244), percentages of polymorphic sites, observed heterozygosities and nucleotide diversity (Table 2). The highest genetic diversity was found in populations GEIR, SCAD and MORA, and HOST, which have the highest numbers of polymorphic sites (GEIR: 6 952, HOST:6 892, SCAD: 6 870, MORA:6 814), highest percentages of polymorphic sites and the highest observed heterozygosities and nucleotide diversity (Table 2). We found no evidence for inbreeding in any of the populations and 12 of the 18 populations analysed had infinite Ne (Table 2). Two populations (BUAI and CHRU) had very small Ne values estimated through the HE method, suggesting these populations have gone through bottlenecks, and three populations (FADA, AROI and STRU) had very low small Ne values estimated through the LD method, suggesting substructure or immigration of different genotypes.

**Table 2:** Summary of genetic statistics for 18 populations. Statistics shown for analyses including only nucleotide positions that are polymorphic in at least one population (V) and for analyses including all nucleotide positions. From left to right: abbreviation of lake name (pop_ID), average number of individuals genotyped at each locus (N), number of variable nucleotide sites unique to a population (Private), number of polymorphic nucleotide sites, percentage of polymorphic sites, average frequency of the major allele (P), average observed heterozygosity per locus (Hobs), average nucleotide diversity (π), average inbreeding coefficient (*F*_IS_), and effective populations sizes (Ne) and their confidence intervals (in parentheses) estimated through the LD and the HE methods

pop _ID	Ind	Private	Poly_Sites	%Poly_loci (V)	%Poly_loci	*P* (V)	*P*	ObsHet (V)	ObsHet	π (V)	π	*F*_IS_ (V)	*F*_IS_	Ne (LD)	Ne (HE)
FADA	14.66	0	6108	0.54	66.86	0.86	0.999	0.178	0.0015	0.197	0.0016	0.076	0.0006	5 (5–5)	∞ (∞–∞)
IALA	17.76	6	4933	0.44	54.00	0.90	0.999	0.151	0.0012	0.151	0.0012	0.003	0.0000	∞ (∞–∞)	∞ (896.6–∞)
BUAI	16.80	31	2390	0.21	26.16	0.96	1.000	0.059	0.0005	0.055	0.0004	−0.007	−0.0001	181.7 (169–196.4)	19.2 (14.1–30.5)
AONG	17.72	1	5305	0.47	58.07	0.86	0.999	0.187	0.0015	0.189	0.0015	0.008	0.0001	∞ (∞–∞)	∞ (∞–∞)
GEIR	16.44	0	6952	0.62	76.10	0.84	0.999	0.222	0.0018	0.232	0.0019	0.034	0.0003	∞ (∞–∞)	∞ (∞–∞)
EISI	16.55	1	4221	0.38	46.21	0.91	0.999	0.134	0.0011	0.135	0.0011	0.008	0.0001	243.2 (229.7–258.4)	∞ (∞–∞)
HOST	18.68	0	6829	0.61	74.76	0.84	0.999	0.220	0.0018	0.223	0.0018	0.013	0.0001	157.6 (154.5–160.8)	∞ (∞–∞)
REIV	18.69	1	6302	0.56	68.99	0.84	0.999	0.221	0.0018	0.220	0.0018	0.004	0.0000	∞ (∞–∞)	∞ (515.2–∞)
AROI	18.66	1	4649	0.41	50.89	0.91	0.999	0.136	0.0011	0.134	0.0011	−0.002	0.0000	6.3 (6.2–6.3)	142.9 (49.9–∞)
GILL	16.50	0	5210	0.46	57.03	0.87	0.999	0.180	0.0015	0.188	0.0015	0.022	0.0002	∞ (∞–∞)	∞ (∞–∞)
TROS	19.47	0	6514	0.58	71.31	0.85	0.999	0.213	0.0017	0.215	0.0017	0.008	0.0001	∞ (∞–∞)	∞ (∞–∞)
STRU	15.57	0	5437	0.49	59.52	0.88	0.999	0.175	0.0014	0.172	0.0014	0.000	0.0000	10 (10–10.1)	4620.2 (72.5–∞)
TORM	18.63	1	5837	0.52	63.90	0.86	0.999	0.200	0.0016	0.202	0.0016	0.008	0.0001	∞ (∞–∞)	∞ (∞–∞)
BHAR	17.66	0	6460	0.58	70.72	0.85	0.999	0.216	0.0018	0.217	0.0018	0.005	0.0000	∞ (∞–∞)	∞ (∞–∞)
MORA	20.66	0	6814	0.61	74.59	0.84	0.999	0.224	0.0018	0.224	0.0018	0.005	0.0000	∞ (∞–∞)	∞ (∞–∞)
SCAD	16.67	0	6870	0.61	75.21	0.84	0.999	0.228	0.0019	0.231	0.0019	0.011	0.0001	∞ (∞–∞)	∞ (∞–∞)
DAIM	19.62	7	3261	0.29	35.70	0.92	0.999	0.115	0.0009	0.113	0.0009	−0.003	0.0000	35.1 (34.7–35.5)	52 (27.4–618.1)
CHRU	16.84	25	1244	0.11	13.62	0.97	1.000	0.051	0.0004	0.047	0.0004	−0.007	−0.0001	∞ (∞–∞)	10.9 (7.9–17.8)

Genetic divergence among populations as represented by *F*_ST_ values was generally high (average *F*_ST_ = 0.200), but we found high variability among comparisons (Table S10, Supporting information). The lowest *F*_ST_ values were found between populations from the same catchment that have streams connecting them: SCAD and MORA (*F*_ST_ = 0.023), and TROS and GILL (*F*_ST_ = 0.024). Populations that have connections to the sea, such as HOST and GEIR, also tended to be less differentiated. The highest genetic divergence was found between the two least genetically diverse populations, BUAI and CHRU (*F*_ST_ = 0.575). Comparisons involving these two populations generally had high *F*_ST_ values (Table S10, Supporting information).

**Figure 3:**
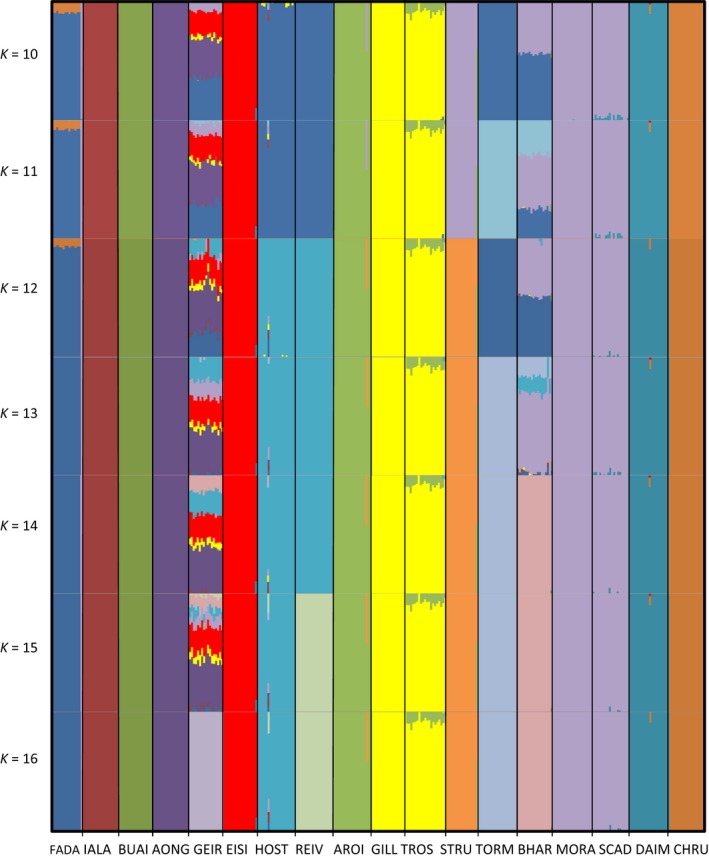
Plot of Bayesian population assignment test based on 9 135 markers with the software faststructure. Each vertical line represents an individual, and colours indicate the proportion of an individual's genotype assigned to a particular cluster. The populations listed from left to right correspond to populations starting on the northeast of North Uist, westwards via the north of the island and back east via the south of the island.

The Bayesian clustering analysis with faststructure showed that statistically *K* = 16 was the best supported number of clusters. Each step from *k* = 10 to *k* = 15 gradually differentiated among geographically close populations, and at *K* = 16, 14 clusters were formed by individual populations and two clusters were formed by pairs of populations from the same catchment (TROS and GILL, MORA and SCAD) (Fig. 3). The PCA (Fig. 2c) and the NJ tree (Fig. S1, Supporting information) largely reflected this structure. Genetic structure was not correlated with geographic distances (*r *=* *−0.007, *P *=* *0.73).

### Phenotype–environment–genotype associations

**Figure 4:**
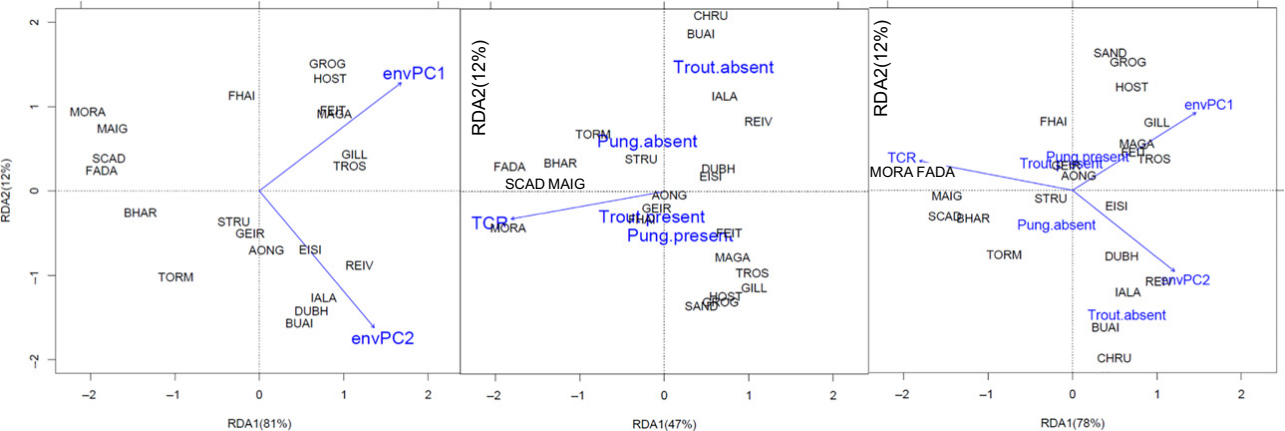
Distance biplot of the RDA ordination of descriptors of phenotypic variation constrained by the environmental variables (reduced data set of 23 lakes showed). Only the environmental factors retained by backward selection (*P* < 0.05) are shown. Quantitative explanatory variables are indicated by arrows. The bottom and left‐hand scales are for the objects, and the top and right‐hand scales are for the explanatory variables. Distances between sites (lakes) points approximate their Euclidean distances. Distances between centroids and between centroids and sites points approximate their Euclidean distances.

The RDA showed that when all phenotypic traits were analysed together the abiotic environmental variables envPC1 and envPC2 explained 36% of the variation in the phenotype across populations when all 27 populations were included and 37% of the phenotypic variation when 23 populations with more detailed ecological data were included. The relationship was highly significant (Table 3) and envPC1 and envPC2 had similar loadings on both RDA axes (see supporting online information for detailed results of all the RDA analyses performed). The analyses showed a continuum of correlated phenotypes and abiotic environments ranging from populations of fish with no armour or dorsal and pelvic spines and a small body, living in lakes with low environmental PC1 and PC2 (i.e. acidic, deep, oligotrophic lakes) to populations of fish with pelvic and dorsal spines and armour and deeper and longer bodies living in alkaline, shallow lakes (Fig. 4).

**Table 3:** Results of permutations tests on RDA analyses and the proportion of the phenotypic variation explained by each model (adjust *R* ^2^) for the complete data set of 27 populations and the reduced ones

Best model	Adjust *R* ^2^	d.f.	Var	*F*	Pr (>F)
All 27 populations					
env_PC1 + env_PC2	0.356	2	4.054	8.182	0.001***
%*P. pungitius* + trout presence	0.259	2	3.156	5.534	0.007**
env_PC1 + env_PC2 + %*P. pungitius* + trout presence	0.454	4	5.531	5.570	0.001***
Reduced data set – 23 populations					
env_PC1 + env_PC2	0.369	2	4.263	7.429	0.001***
*P. pungitius* presence + trout presence + TCR	0.511	3	5.776	8.661	0.001***
env_PC1 + env_ PC2 + *P. pungitius*_presence + trout presence + TCR	0.594	5	6.865	7.446	0.001***
Reduced data set – 18 populations					
gen_PC1	0.088	1	1.4171	2.6418	0.08.

Significance codes: *P* < 0.001 ‘***’, *P* < 0.01 ‘**’, *P* < 0.05 ‘*’, *P* < 0.1 ‘.’, *P* > 0.1 ‘n.s.’

The inclusion of TCR in the matrix of biotic explanatory variables greatly increased the explanatory power of the biotic model. In the complete data set (27 populations), proportion of *P. punigitius* and trout presence explained 25% of the phenotypic variance, while in the reduced data set *P. pungitius* presence and trout presence and TCR explained 51% of the phenotypic variance (Table 3). Therefore, we focused on the results of the latter model. The relationship between phenotypes and the three biotic variables was highly significant (Table 3), but only the first two RDA axes, which explain 90% of the phenotype–environment relationship, were significant (online Supporting information). The highest loadings on RDA1 and RDA2 were TCR and trout presence, respectively. The first axis differentiated between lakes with higher abundance of trout where fish have low or no armour and spines and are small bodied, vs. lakes with low density or no trout where fish are plated and have longer bodies (Fig. 4).

As expected, the models combining the biotic and abiotic variables in one explanatory matrix explained a higher percentage of the phenotypic variation than the biotic or abiotic variables separately (Table 3, Fig. 4). However, the phenotypic variation explained by all variables together is less than the sum of the variations explained by the two groups of variables. Therefore, some amount of variation is explained jointly by the two sets, consistent with the fact that biotic and abiotic variables are intercorrelated.

The RDA relating phenotypes and genetic axes of variation revealed that only the first axis of genetic variation (‘genPC1’) explained some of the phenotypic variation (8%) and this model was not significant (Table 3).

The analyses of the association between individual phenotypic axes of variation and environmental and genetic variables revealed that the ‘Armour PC’ was similarly significantly associated with abiotic and biotic environmental variables and genetic variables, and that combined envPC1, TCR and several genetic PCs explained 79% of the variation in armour traits found across lakes (Table 4a). The ‘SL PC’ was only significantly associated with biotic environmental variables and genetic variables, but not the abiotic environment. Combined, the number of *G. arcuatus*, proportion of *P. pungitius* and genPC9 explained 50% of the variation in fish length across populations. Contrary to the ‘SL PC’, the ‘1st body shape PC’ was only significantly associated with abiotic environmental variables and genetic variables, but not with the biotic environment. Combined, several axes of abiotic and genetic variables explained 79% of the variation in body shape across populations. The ‘2nd body shape PC’ was significantly associated with biotic, abiotic and genetic variables. However, when all explanatory variables were combined the biotic variables were excluded from the best fit model, which included 4 axes of abiotic variation and 4 axes of genetic variation and explained 87% of the variation in body shape.

**Table 4:** Results of multiple regressions. Shown are the models that best explained variation in the response data after stepwise backward selection of explanatory variables by exact AIC, the *F*‐statistics of the 1000 permutations of residuals of full model, and the proportion of the (a) phenotypic variation and (b) genetic variation explained by each model (adjust *R* ^2^)

Best model	AIC	*F*	d.f.	*P*	Adjust *R* ^2^
(a)					
*Response: Phen_PC1 (‘armour PC’)*					
Abiotic: envPC1 + envPC2	29.64	13.579	2,24	0.001	0.492
Biotic: Gyrodactylus + Schistochefalus + TCR	19.82	15.267	3,19	0.001	0.661
Genetic: genPC1 + genPC2 + genPC3 + genPC5 + genPC6 + genPC7 + genPC10	25.79	3.434	7,10	0.038	0.501
Combined: envPC1 + TCR + genPC1 + genPC5 + genPC6 + genPC7	10.34	10.430	6,9	0.001	0.790
*Response: Phen_PC2 (‘SL PC’)*					
Abiotic:envPC8 + envPC9	−33.12	1.945	2,24	0.183	0.068
Biotic:% *P. pungitius* + Gyrodactylus	−39.20	6.365	2,20	0.006	0.328
Genetic: genPC4 + genPC6 + genPC9 + genPC10	−25.58	3.601	4,13	0.035	0.380
Combined: % Pungitius + Gyrodactylus + genPC4 + genPC6 + genPC9 + genPC10	−28.12	3.549	6,9	0.043	0.505
*Response: Phen_PC3 (‘1st body shape PC’)*					
Abiotic:envPC1 + envPC2 + envPC3 + envPC6	−53.94	11.517	4,22	0.001	0.618
Biotic: Gyrodactylus	−26.26	2.626	1,21	0.121	0.069
Genetic: genPC1 + genPC10	−39.83	8.269	2,15	0.004	0.461
Combined: envPC3 + envPC6 + genPC1 + genPC10	−50.57	15.370	4,11	0.000	0.793
*Response: Phen_PC4 (‘2nd body shape PC’)*					
Abiotic:envPC2 + envPC3 + envPC5 + envPC9	−25.42	5.627	4,22	0.005	0.416
Biotic: Gyrodactylus + TCR	−16.69	4.296	2,20	0.029	0.230
Genetic: genPC1 + genPC2 + genPC3 + genPC4 + genPC5 + genPC6 + genPC10	−29.78	12.630	7,10	0.000	0.827
Combined: envPC2 + envPC3 + envPC5 + envPC9 + genPC2 + genPC3 + genPC4 + genPC5 + genPC10	−33.18	12.600	9,6	0.003	0.874
(b)					
*Response: genPC1*					
Abiotic: envPC1 + envPC2 + envPC3 + envPC4 + envPC5 + envPC7 + envPC10	72.71	3.260	4,7	0.045	0.482
Biotic: %*P. pungitius* + TCR	63.99	3.034	2,13	0.082	0.213
*Response: genPC2*					
Abiotic: envPC2 + envPC3 + envPC9	75.42	3.373	3,14	0.049	0.295
Biotic: gyro + TCR	69.25	3.186	2,13	0.074	0.226
*Response: genPC3*					
Abiotic:envPC1 + envPC3 + envPC5 + envPC8 + envPC9	68.57	4.600	5,12	0.014	0.514
Biotic: none	—	—	—	—	—
*Response: genPC4*					
Abiotic:envPC3 + envPC4 + envPC8 + envPC10	70.41	2.810	4,13	0.070	0.299
Biotic: none	—	—	—	—	—
*Response: genPC5*					
Abiotic: envPC3 + envPC4 + envPC8 + envPC10	69.63	2.108	3,14	0.145	0.164
Biotic: none	—	—	—	—	—
*Response: genPC6*					
Abiotic:envPC2 + envPC3 + envPC6 + envPC7 + envPC8 + envPC9	55.73	5.461	6,11	0.008	0.612
Biotic: none	—	—	—	—	—
*Response: genPC7*					
Abiotic: envPC3 + envPC4 + envPC8 + envPC10	—	—	—	—	—
Biotic: % *P. pungitius*	43.92	10.600	1,14	0.006	0.39
*Response: genPC8*					
Abiotic:envPC1 + envPC8	60.33	4.661	2,15	0.027	0.301
Biotic: none	—	—	—	—	—
*Response: genPC9*					
Abiotic: envPC2 + envPC4 + envPC6 + envPC8 + envPC9	53.89	4.478	5,12	0.016	0.506
Biotic: none	—	—	—	—	—
*Response: genPC10*					
Abiotic: envPC1 + envPC2 + envPC4 + envPC5 + envPC6 + envPC7 + envPC9	49.79	4.594	7,10	0.015	0.597
Biotic: % *P. pungitius*	55.01	3.720	1,14	0.074	0.153

The analyses of the association between individual genetic axes of variation and the environment revealed that genetic variation was largely associated with the abiotic environment. Seven of 10 genomic PCs were significantly associated with abiotic environmental PCs, 1 PC was significantly associated with the biotic environment (genPC8) and 2 PCs (genPC4, genPC5) were not associated with the environment (Table 4b).

## Discussion

### Phenotypic variation and its association with the environment

In this study, we found large morphological variation among populations of three‐spined stickleback from North Uist, particularly in armour traits, but also in body length and shape. Our results also show that extreme morphologies that have not been reported in other stickleback radiations, namely small fish with no dorsal and pelvic spines or armour, are present in some of the lakes. Phenotypic variation appears to have a modular structure to some extent, with certain groups of traits being quite strongly intercorrelated, especially armour traits. This could arise because traits are genetically correlated (Miller *et al*. 2014) or because the environment is driving the correlation between traits. Genetic correlations could arise either because traits are controlled by the same underlying, or physically linked genes, or because the genes are in linkage disequilibrium. It seems likely that some traits (for example dorsal spines one and two, or different measures of pelvis size) have some measure of shared genetic control, but we know that other pairs of traits (such as plate number and pelvis size) are not controlled by the same genes (Colosimo *et al*. 2005; Chan *et al*. 2010). However, the fact that we found that there is correlation of traits across populations, even when those traits are not correlated within populations, is consistent with trait correlations being a product of environmentally driven correlational selection which varies in its form across populations (Sinervo & Svensson 2002). The fact that morphological differences across populations are strongly associated with the abiotic and biotic environment further supports this idea.

The strong association between phenotypes and abiotic environmental variation across lakes came from stickleback inhabiting acidic, low in ion composition, deep and oligotrophic lakes exhibiting extreme reduction of armour traits and body length compared to sticklebacks inhabiting more alkaline and eutrophic waters. However, variation in some traits was better explained by predation and competition regimes. Presence of nine‐spined stickleback and density of brown trout (as measured by TCR) explained a high percentage of the phenotypic variation. Consistent with MacColl *et al*. (2013) for an overlapping set of lakes, we found that three‐spined stickleback are longer when in sympatry with nine‐spined stickleback. In the four lakes without trout, which are all small and shallow but otherwise very different in abiotic environment, three‐spined stickleback appear to have converged on a rather similar body shape (Fig. 4). Perhaps more surprisingly, armour reduction seems to be associated with higher trout density. The latter result appears contrary to previous findings of positive associations between spine length and predation (Moodie 1972; Gross 1978; Reimchen 1994) and the report that salmonids are uncommon in low pH lakes (Reimchen 1992). The relative importance and directionality of the selective pressures of trout presence and abundance on armour diversification in sticklebacks from North Uist is a controversial issue (Spence *et al*. 2013; MacColl & Aucott 2014; Smith *et al*. 2014), but possible explanations come from the fact that trout density on North Uist is negatively associated with trout size (MacColl & Aucott 2014). Larger trout may be more voracious predators, at least of adult stickleback, a hypothesis that is supported by analysis of trout stomach contents (J. Whiting, S. Young and A. MacColl, unpublished data). This effect on the evolution of stickleback armour could be exacerbated if the armour of large stickleback is more effective than the armour of small stickleback in defence against predation.

The fact that we found phenotypic variation to be strongly associated with environmental variation across lakes is consistent with central tenets of the ecological theory of adaptive radiation (Schluter 2000). Also, in keeping with Darwin's (1859) intuition about agents of selection, the variation in phenotypes is better predicted by biotic variables, especially the density of predators and competitors. This is also expected from classical ideas about adaptive radiation (Lack 1947; Simpson 1953), but has seldom been documented at this scale before. Nonetheless, although biotic variation appears more important than abiotic variation simply in terms of the amount of variation explained, our analyses suggest that biotic and abiotic variables are intercorrelated. Therefore, it is not really possible to separate the two ecologically, as the former presumably develops on the foundations of the latter. It is then interesting to speculate on the (eco)evolutionary consequences of the way in which ecological communities, and the selection they cause, are predicated on the abiotic environment in which they develop. Our results suggest that presence and abundance of nine‐spine stickleback and trout are most likely associated with physical and chemical characteristics of the lakes. Absence of trout has been previously associated with small lakes with low pH (Reimchen 1992), and alkaline metal concentrations seem to be a good predictor of presence of nine‐spined stickleback (MacColl *et al*. 2013). It seems likely that abiotic conditions are more deterministic (although there may well be some level of chemical feedback for example that depends on biotic communities), whereas biotic conditions are more stochastic and dynamic, as a result of both dispersal and ‘Red Queen’ processes. Such ideas seem seldom considered in the adaptive radiation literature. In the case of the three‐spined stickleback from North Uist, phenotypic variation is shaped not just by physical and chemical characteristics of a lake or by predation or competition regimes separately, but by the combination of all of these factors and their interactions.

### Genetic clustering and divergence

We found a strong genetic divergence among the populations analysed, comparable to that found among freshwater populations that are geographically further apart in other stickleback systems (Hohenlohe *et al*. 2010; Deagle *et al*. 2011; Jones *et al*. 2012). Here, we assessed only the genome‐wide patterns of genetic divergence between populations, which are expected to be determined by a combination of adaptive and neutral processes (Kimura 1984). Local adaptation is achieved by a combination of environmental filtering of genotypes through selection against migrants or individual preference to remain in a particular environment (Williams 1966; Kawecki & Ebert 2004; Orsini *et al*. 2013), and neutral isolation by distance (IBD; Wright 1943) that reduces gene flow among geographically distant or separated populations. If differential rates of gene flow are responsible for genetic divergence, we should find a correlation between genetic divergence and geographic factors such as landscape barriers and geographic distances (Wright 1943). Most of the lakes sampled were in separate catchments (although this could have been different in the past) and therefore joined only through connections to the sea. However, several lakes apparently have no accessible, extant connection to the sea, even if they are very close to it. We assumed that lakes in separate catchments were independently colonized by marine sticklebacks, and tested for IBD using distances through freshwater when possible or through the sea otherwise, but results were not significant. Nonetheless, there does appear to be some role for geography in determining genetic divergence among populations. The faststructure analyses clearly indicated that most genetic clusters were formed by populations that are either in the same catchment or geographically near each other, which suggests a role for geography in the genetic divergence of populations.

### Ecological and genomic axes of morphological diversity

We hypothesized the observed phenotypic variation among populations to be the result of either adaptation, neutral genetic drift within relatively isolated populations, or common ancestry. For all cases, we would predict a correlation between genomewide genetic variation and multivariate phenotypic variation. However, when all phenotypic traits were analysed together, we found that genetic variation explained very little of the variation in the phenotype across populations. To the extent that the phenotypes we examined are heritable, an explanation for the lack of association between the two is that phenotypes are genetically controlled by a relatively small number of loci that are not in linkage disequilibrium with the markers we analysed. If this is the case then the divergence represented by the genomewide F_ST_ and PC values is mostly the result of neutral processes (gene flow and genetic drift) and therefore not associated with the phenotypes. This would be especially true if much of the overall divergence between populations is the stochastic result of drift, which should be expected when population sizes are small, which seems to be the case for a few populations, but not most of them.

The strongest evidence supporting the fact that we need to look at factors other than just genetic ones to explain phenotypic variation across North Uist is that we find similar morphologies both in genetically very divergent populations, such as BUAI and CHRU, as well as in genetically similar populations, such as MORA and SCAD (Fig. 2). This result, together with the fact that we recover the signal of a strong effect of environmental factors in terms of fish morphology, suggests that such factors are able to shape fish phenotypes in a predictable direction regardless of their genetic starting point, although it is also possible that some traits are under complex genetic control, but that not all causal SNPs are shared between populations. Another possibility is that phenotypic variation among lakes could be at least partly environmentally induced, that is plastic (although the form of the response to environment, the reaction norm, could still be under genetic control). Alternatively, as described above, if phenotypic differentiation is associated with fixed genetic differences and controlled by a relatively small portion of the genome, heritable divergence in selected traits could occur without leaving a signature across the genome. Future studies focusing on outlier analyses will be able to shed more light on potential statistical associations of phenotypic differences with outlier‐specific genotypes.

Despite the lack of association between genetic variation and phenotype when all phenotypic traits were analysed together, the multiple linear regressions revealed strong associations between individual traits and environmental and genetic variation, supporting the idea of a combined action of these two factors shaping variation in those traits. The environment is probably to influence trait variation among populations both within the lifespan of an individual (phenotypic plasticity) and over multiple generations through selection on different genotypes (Robinson & Wilson 1996). So far evidence points in the direction of armour traits being highly heritable (Peichel *et al*. 2001; Chan *et al*. 2010) and therefore variation in armour morphology across lakes most likely being the result of different genotypes being selected in different environments. For body shape variation, there is evidence supporting the idea that adaptive phenotypic plasticity plays an important role in establishing differences in body shape between stickleback populations (Day *et al*. 1994; Wund *et al*. 2008; Reid & Peichel 2010). In addition, the significant associations between genetic and environmental variation suggest that the environment is also shaping genetic variation. However, it is also possible that such associations arise because of structure in patterns of colonization: if separate lineages of marine stickleback established the freshwater populations in different parts of North Uist, this could lead to association between overall genomic variation and environment.

Together, our results of strong association between individual phenotypic traits and environmental variation suggest that differences in body shape among stickleback populations are the result of responses to environmental factors, while the genetic variation and the high genome‐wide F_ST_ values among populations support a scenario of either selection for specific genotypes or genetic drift due to small population sizes resulting in strong divergence among populations across the genome.

I.S.M. and A.D.C.M. conceived and designed the study, did field work, did data analyses and the writing. D.D. did field work and contributed to the data collection and analyses. P.A.H. contributed to the making of the RAD libraries, data analyses and the writing.

## Data accessibility

Lake depth coordinates, Individual morphological and genomic PCs, and faststructure files: http://dx.doi.org/10.5061/dryad.rb5s1.

## Supporting information


**Fig. S1** Neighbour‐Joining tree based on 9 135 genetic markers for 324 individuals from18 populations.
**Table S1** Number of individuals divided by males and females (F/M) collected for processing, individuals respectively.
**Table S2a** Collection date of abiotic environmental variables and measured physical and chemical characteristics of 27 lochs, from left to right: mean depth (Mean depth), maximum depth (Max depth), surface area (area), pH, conductivity, salinity, composition of the margin substrate (proportions of mud, soft peat, hard peat, gravel, rock).
**Table S2b** Water light spectra (orange ratio), chlorophyll A and chemical characteristics of 27 lochs.
**Table S3** Trait means (M) and standard deviations (SD) per population in mm (from left to right).
**Table S4** Loadings of landmarks on the first two PC axes and the percent variance explained by each axis.
**Table S5** Population‐level summary statistics for RAD sequences.
**Table S6** Loadings of morphological variables on the 10 PC axes and the percent variance explained by each axis.
**Table S7** Pearson correlation coefficients (r) among traits for all populations pooled together and each population separately.
**Table S8** Loadings of abiotic environmental variables on the axes of environmental variation and the percent variance explained by each axis.
**Table S9** Pearson correlation coefficients (r), P‐values and FDR corrected P‐values of correlations among abiotic environmental variables.
**Table S10** Summary statistics for the 18 populations from North Uist with RAD sequencing data as outputted by the program populations.
**Table S11** F_ST_ values based on whole genome among 18 freshwater populations of three‐spined stickleback from North Uist.

## References

[mec13746-bib-0001] Anderson MJ , Legendre P (1999) An empirical comparison of permutation methods for tests of partial regression coefficients in a linear model. Journal of Statistical Computation and Simulation, 62, 271–303.

[mec13746-bib-0002] Ballantyne C (2010) Extent and deglacial chronology of the last British‐Irish Ice Sheet: implications of exposure dating using cosmogenic isotopes. Journal of Quaternary Science, 25, 515–534.

[mec13746-bib-0100] Barrett RD , Rogers SM , Schluter D (2008) Natural selection on a major armor gene in threespine stickleback. Science, 322, 255–257. 18755942 10.1126/science.1159978

[mec13746-bib-0004] Benjamini Y , Hochberg Y (1995) Controlling the false discovery rate: a practical and powerful approach to multiple testing. Journal of the Royal Statistical Society. Series B (Methodological), 57, 289–300.

[mec13746-bib-0005] Borcard D , Gillet F , Legendre P (2011) Numerical Ecology with R. Springer, New York, USA.

[mec13746-bib-0006] Bourgeois JF , Blouw DM , Koenings JP , Bell MA (1994) Multivariate analysis of geographic covariance between phenotypes and environments in the threespine stickleback, *Gasterosteus aculeatus*, from the Cook Inlet area, Alaska. Canadian Journal of Zoology, 72, 1497–1509.

[mec13746-bib-0007] Catchen JM , Amores A , Hohenlohe P , Cresko W , Postlethwait JH (2011) Stacks: building and genotyping loci de novo from short‐read sequences. G3: Genes, Genomes, Genetics, 1, 171–182. 22384329 10.1534/g3.111.000240 PMC3276136

[mec13746-bib-0008] Catchen J , Hohenlohe PA , Bassham S , Amores A , Cresko WA (2013) Stacks: an analysis tool set for population genomics. Molecular Ecology, 22, 3124–3140. 23701397 10.1111/mec.12354 PMC3936987

[mec13746-bib-0009] Chan YF , Marks ME , Jones FC *et al* (2010) Adaptive evolution of pelvic reduction in sticklebacks by recurrent deletion of a Pitx1 enhancer. Science, 327, 302–305. 20007865 10.1126/science.1182213 PMC3109066

[mec13746-bib-0010] Colosimo PF , Hosemann KE , Balabhadra S *et al* (2005) Widespread parallel evolution in sticklebacks by repeated fixation of ectodysplasin alleles. Science, 307, 1928–1933. 15790847 10.1126/science.1107239

[mec13746-bib-0103] Cresko WA , Amores A , Wilson C *et al* (2004) Parallel genetic basis for repeated evolution of armor loss in Alaskan threespine stickleback populations. Proceedings of the National Academy of Sciences of the United States of America, 101, 6050–6055. 15069186 10.1073/pnas.0308479101 PMC395921

[mec13746-bib-0011] Darwin C (1859) The Origin of Species. Murray, London.

[mec13746-bib-0012] Day T , Pritchard J , Schluter D (1994) A comparison of two sticklebacks. Evolution, 48, 1723–1734. 10.1111/j.1558-5646.1994.tb02208.x 28568405

[mec13746-bib-0013] Deagle BE , Jones FC , Chan YF *et al* (2011) Population genomics of parallel phenotypic evolution in stickleback across stream–lake ecological transitions. Proceedings of the Royal Society of London B: Biological Sciences, 279, 1277–1286. 10.1098/rspb.2011.1552 PMC3282360 21976692

[mec13746-bib-0014] Do C , Waples RS , Peel D , Macbeth GM , Tillett BJ , Ovenden JR (2014) NeEstimator V2: re‐implementation of software for the estimation of contemporary effective population size (Ne) from genetic data. Molecular Ecology Resources, 14, 209–214. 23992227 10.1111/1755-0998.12157

[mec13746-bib-0015] Dryden IL , Mardia KV (1998). Statistical Analysis of Shape. Wiley, London.

[mec13746-bib-0016] Endler JA , Houde AE (1995) Geographic variation in female preferences for male traits in *Poecilia reticulata* . Evolution, 49, 456–468. 10.1111/j.1558-5646.1995.tb02278.x 28565093

[mec13746-bib-0106] Etter PD , Preston JL , Bassham S *et al* (2011) Local de novo assembly of RAD paired‐end contigs using short sequencing reads. PloS One, 6, e18561. 21541009 10.1371/journal.pone.0018561 PMC3076424

[mec13746-bib-0017] Excoffier L , Estoup A , Cornuet JM (2005) Bayesian analysis of an admixture model with mutations and arbitrarily linked markers. Genetics, 169, 1727–1738. 15654099 10.1534/genetics.104.036236 PMC1449551

[mec13746-bib-0018] Ezekiel M (1930) Methods of Correlational Analysis. Wiley, New York, New York.

[mec13746-bib-0019] Giles N (1983) The possible role of environmental calcium levels during the evolution of phenotypic diversity in Outer Hebridean populations of the Three‐spined stickleback, *Gasterosteus aculeatus* . Journal of Zoology, 199, 535–544.

[mec13746-bib-0020] Gross HP (1978) Natural selection by predators on the defensive apparatus of the three‐spined stickleback, *Gasterosteus aculeatus* L. Canadian Journal of Zoology, 56, 398–413.

[mec13746-bib-0021] Hart PJ (2003) Habitat use and feeding behaviour in two closely related fish species, the three‐spined and nine‐spined stickleback: an experimental analysis. Journal of Animal Ecology, 72, 777–783.

[mec13746-bib-0022] Hohenlohe PA , Bassham S , Etter PD , *et al* (2010) Population genomics of parallel adaptation in threespine stickleback using sequenced RAD tags. PLoS Genetics, 6, e1000862. 20195501 10.1371/journal.pgen.1000862 PMC2829049

[mec13746-bib-0025] Jombart T (2008) adegenet: an R package for the multivariate analysis of genetic markers. Bioinformatics, 24, 1403–1405. 18397895 10.1093/bioinformatics/btn129

[mec13746-bib-0026] Jones FC , Grabherr MG , Chan YF *et al* (2012) The genomic basis of adaptive evolution in threespine sticklebacks. Nature, 484, 55–61. 22481358 10.1038/nature10944 PMC3322419

[mec13746-bib-0027] Kawecki TJ , Ebert D (2004) Conceptual issues in local adaptation. Ecology Letters, 7, 1225–1241.

[mec13746-bib-0101] Kimura M (1984) The Neutral Theory of Molecular Evolution. Cambridge University Press, Cambridge, UK.

[mec13746-bib-0028] Klingenberg CP (2010) Evolution and development of shape: integrating quantitative approaches. Nature Reviews Genetics, 11, 623–635. 10.1038/nrg2829 20697423

[mec13746-bib-0029] Klingenberg CP (2011) MorphoJ: an integrated software package for geometric morphometrics. Molecular Ecology Resources, 11, 353–357. 21429143 10.1111/j.1755-0998.2010.02924.x

[mec13746-bib-0030] Kocher TD (2004) Adaptive evolution and explosive speciation: the cichlid fish model. Nature Reviews Genetics, 5, 288–298. 10.1038/nrg1316 15131652

[mec13746-bib-0031] Lack D (1947) Darwin's Finches. Cambridge University Press, Cambridge.

[mec13746-bib-0032] Langerhans RB , Layman CA , Shokrollahi A *et al* (2004) Predator‐driven phenotypic diversification in *Gambusia affinis* . Evolution, 58, 2305–2318. 15562692 10.1111/j.0014-3820.2004.tb01605.x

[mec13746-bib-0033] Losos JB , Jackman TR , Larson A *et al* (1998) Contingency and determinism in replicated adaptive radiations of island lizards. Science, 279, 2115–2118. 9516114 10.1126/science.279.5359.2115

[mec13746-bib-0034] Loy A , Cataudella S , Corti M (1996) Shape changes during the growth of the Sea Bass, *Dicentrarchus labrax* (Teleostea: Perciformes), in relation to different rearing conditions: an application of thin‐plate spline regression analysis. Nato ASI Series A Life Sciences, 284, 399–406.

[mec13746-bib-0035] MacColl ADC (2009) Parasite burdens differ between sympatric three‐spined stickleback species. Ecography, 32, 153–160.

[mec13746-bib-0036] MacColl ADC , Aucott B (2014) Inappropriate analysis does not reveal the ecological causes of evolution of stickleback armour: a critique of Spence *et al*. 2013. Ecology and Evolution, 4, 3509–3513. 25478143 10.1002/ece3.1179 PMC4224526

[mec13746-bib-0037] MacColl ADC , Nagar AE , Roij J (2013) The evolutionary ecology of dwarfism in three‐spined sticklebacks. Journal of Animal Ecology, 82, 642–652. 23237226 10.1111/1365-2656.12028

[mec13746-bib-0038] Marchinko KB (2009) Predation's role in repeated phenotypic and genetic divergence of armor in threespine stickleback. Evolution, 63, 127–138. 18803682 10.1111/j.1558-5646.2008.00529.x

[mec13746-bib-0039] Miller CT , Glazer AM , Summers BR *et al* (2014) Modular skeletal evolution in sticklebacks is controlled by additive and clustered quantitative trait loci. Genetics, 197, 405–420. 24652999 10.1534/genetics.114.162420 PMC4012497

[mec13746-bib-0040] Moodie GEE (1972) Morphology, life history, and ecology of an unusual stickleback (*Gasterosteus aculeatus*) in the Queen Charlotte Islands, Canada. Canadian Journal of Zoology, 50, 721–732.

[mec13746-bib-0041] Murray J , Pullar L (1910) Bathymetrical Survey of the Freshwater Lochs of Scotland. Challenger Office, Edinburgh.

[mec13746-bib-0042] Nosil P , Crespi BJ (2006) Experimental evidence that predation promotes divergence in adaptive radiation. Proceedings of the National Academy of Sciences, 103, 9090–9095. 10.1073/pnas.0601575103 PMC1482571 16754870

[mec13746-bib-0043] Nosil P , Reimchen TE (2005) Ecological opportunity and levels of morphological variance within freshwater stickleback populations. Biological Journal of the Linnean Society, 86, 297–308.

[mec13746-bib-0044] Nosil P , Funk DJ , Ortiz‐Barrientos D (2009) Divergent selection and heterogeneous genomic divergence. Molecular Ecology, 18, 375–402. 19143936 10.1111/j.1365-294X.2008.03946.x

[mec13746-bib-0045] Oksanen JF , Blanchet G , Kindt R *et al* (2013) Vegan: Community Ecology Package. R package version 2.0‐10.

[mec13746-bib-0046] Orsini L , Vanoverbeke J , Swillen I *et al* (2013) Drivers of population genetic differentiation in the wild: isolation by dispersal limitation, isolation by adaptation and isolation by colonization. Molecular Ecology, 22, 5983–5999. 24128305 10.1111/mec.12561

[mec13746-bib-0047] Paradis E , Claude J , Strimmer K (2004) APE: analyses of phylogenetics and evolution in R language. Bioinformatics, 20, 289–290. 14734327 10.1093/bioinformatics/btg412

[mec13746-bib-0048] Peichel CL , Nereng KS , Ohgi KA (2001) The genetic architecture of divergence between threespine stickleback species. Nature, 414, 901–905. 11780061 10.1038/414901a

[mec13746-bib-0049] Pfennig DW , Rice AM , Martin RA (2007) Field and experimental evidence for competition's role in phenotypic divergence. Evolution, 61, 257–271. 17348937 10.1111/j.1558-5646.2007.00034.x

[mec13746-bib-0050] Pritchard JK , Stephens M , Donnelly P (2000) Inference of population structure using multilocus genotype data. Genetics, 155, 945–959. 10835412 10.1093/genetics/155.2.945 PMC1461096

[mec13746-bib-0051] Raj A , Stephens M , Pritchard JK (2014) fastSTRUCTURE: variational inference of population structure in large SNP data sets. Genetics, 197, 573–589. 24700103 10.1534/genetics.114.164350 PMC4063916

[mec13746-bib-0052] Rao C (1964) The use and interpretation of principal components analysis in applied research. The Indian Journal of Statistics, Series A, 26, 329–358.

[mec13746-bib-0053] Reid DT , Peichel CL (2010) Perspectives on the genetic architecture of divergence in body shape in sticklebacks. Integrative and Comparative Biology, icq030, 1–10. 10.1093/icb/icq030 PMC2981589 21082067

[mec13746-bib-0054] Reimchen TE (1980) Spine deficiency and polymorphism in a population of *Gasterosteus aculeatus*: an adaptation to predators? Canadian Journal of Zoology, 58, 1232–1244.

[mec13746-bib-0055] Reimchen TE (1992) Naikoon Provincial Park, Queen Charlotte Islands: Biophysical Data for Freshwater Habitats. Ministry of the Environment, Government of British Columbia, Queen Charlotte City, BC, Canada.

[mec13746-bib-0056] Reimchen TE (1994) Predators and morphological evolution in threespine stickleback In: The Evolutionary Biology of the Threespine Stickleback (eds Bell MA, Foster SA), pp. 240–276. Oxford University Press, Oxford, UK

[mec13746-bib-0057] Robinson BW , Wilson DS (1996) Genetic variation and phenotypic plasticity in a trophically polymorphic population of pumpkinseed sunfish (*Lepomis gibbosus*). Evolutionary Ecology, 10, 631–652.

[mec13746-bib-0059] Rohlf FJ (1999) Shape statistics: Procrustes superimpositions and tangent spaces. Journal of Classification, 16, 197–223.

[mec13746-bib-0060] Rohlf FJ (2010a) tpsDig, digitize landmarks and outlines, version 2.16. Department of Ecology and Evolution, State University of New York at Stony Brook, NJ.

[mec13746-bib-0061] Rohlf FJ (2010b) tpsUtil, file utility program, version 1.46. Department of Ecology and Evolution, State University of New York at Stony Brook, NJ.

[mec13746-bib-0062] Rohlf FJ , Marcus LF (1993) A revolution in morphometrics. Trends in Ecology and Evolution, 8, 129–132. 21236128 10.1016/0169-5347(93)90024-J

[mec13746-bib-0063] de Roij J , MacColl ADC (2012) Consistent differences in macroparasite community composition among populations of three‐spined sticklebacks, *Gasterosteus aculeatus* L. Parasitology, 139, 1478–1491. 23025902 10.1017/S0031182012000789

[mec13746-bib-0064] Rundell RJ , Price TD (2009) Adaptive radiation, nonadaptive radiation, ecological speciation and nonecological speciation. Trends in Ecology & Evolution, 24, 394–399. 19409647 10.1016/j.tree.2009.02.007

[mec13746-bib-0065] Schluter D (1994) Experimental evidence that competition promotes divergence in adaptive radiation. Science, 266, 798–801. 17730400 10.1126/science.266.5186.798

[mec13746-bib-0066] Schluter D (1996) Ecological causes of adaptive radiation. American Naturalist, 148, 40–64.

[mec13746-bib-0067] Schluter D (2000) The Ecology of Adaptive Radiation. Oxford University Press, Oxford, UK.

[mec13746-bib-0068] Simpson GG (1953) The Major Features of Evolution. Columbia University Press, New York, New York.

[mec13746-bib-0069] Sinervo B , Svensson E (2002) Correlational selection and the evolution of genomic architecture. Heredity, 89, 329–338. 12399990 10.1038/sj.hdy.6800148

[mec13746-bib-0070] Smith C , Spence R , Barber I *et al* (2014) The role of calcium and predation on plate morph evolution in the three‐spined stickleback (*Gasterosteus aculeatus*). Ecology and Evolution, 4, 3550–3554. 25478147 10.1002/ece3.1180 PMC4224530

[mec13746-bib-0071] Spence R , Wootton RJ , Barber I *et al* (2013) Ecological causes of morphological evolution in the three‐spined stickleback. Ecology and Evolution, 3, 1717–1726. 23789080 10.1002/ece3.581 PMC3686204

[mec13746-bib-0072] Tyerman JG , Bertrand M , Spencer CC *et al* (2008) Experimental demonstration of ecological character displacement. BMC Evolutionary Biology, 8, 34. 18234105 10.1186/1471-2148-8-34 PMC2267161

[mec13746-bib-0104] Vamosi S (2002) Predation sharpens the adaptive peaks: survival trade‐offs in sympatric sticklebacks. Annales Zoologici Fennici, 39, 1–28.

[mec13746-bib-0074] Waples RS , Do C (2008) LDNE: a program for estimating effective population size from data on linkage disequilibrium. Molecular Ecology Resources, 8, 753–756. 21585883 10.1111/j.1755-0998.2007.02061.x

[mec13746-bib-0075] Williams GC (1966) Adaptation and Natural Selection. Princeton University Press, Princeton, New Jersey.

[mec13746-bib-0076] Wright S (1943) Isolation by distance. Genetics, 28, 114–138. 17247074 10.1093/genetics/28.2.114 PMC1209196

[mec13746-bib-0105] Wright S (1951) The genetical structure of populations. Annals of Eugenics, 15, 323–354. 24540312 10.1111/j.1469-1809.1949.tb02451.x

[mec13746-bib-0077] Wu TD , Nacu S (2010) Fast and SNP‐tolerant detection of complex variants and splicing in short reads. Bioinformatics, 26, 873–881. 20147302 10.1093/bioinformatics/btq057 PMC2844994

[mec13746-bib-0078] Wu TD , Watanabe CK (2005) GMAP: a genomic mapping and alignment program for mRNA and EST sequences. Bioinformatics, 21, 1859–1875. 15728110 10.1093/bioinformatics/bti310

[mec13746-bib-0079] Wund MA , Baker JA , Clancy B *et al* (2008) A test of the “flexible stem” model of evolution: ancestral plasticity, genetic accommodation, and morphological divergence in the threespine stickleback radiation. The American Naturalist, 172, 449–462. 10.1086/590966 18729721

[mec13746-bib-0080] Yoder JB , Clancey E , Des Roches S *et al* (2010) Ecological opportunity and the origin of adaptive radiations. Journal of Evolutionary Biology, 23, 1581–1596. 20561138 10.1111/j.1420-9101.2010.02029.x

[mec13746-bib-0081] Zelditch ML , Swiderski DL , Sheets DH *et al* (2004) Geometric Morphometrics for Biologists: A Primer. Elsevier Academic Press, London, UK.

[mec13746-bib-0082] Zhdanova O , Pudovkin AI (2008) Nb_HetEx: a program to estimate the effective number of breeders. Journal of Heredity, 99, 694–695. 18703539 10.1093/jhered/esn061

