# Supplementary material for: The ecology of an adaptive radiation of three‐spined stickleback from North Uist, Scotland
Source: Mol Ecol. 2016 Aug 8;25(17):4319–36. doi: 10.1111/mec.13746 (PMC5031221; doi:10.1111/mec.13746)
Supplement: Supplementary file 1 — Fig. S1 Neighbour‐Joining tree based on 9 135 genetic markers for 324 individuals from18 populations. Table S1 Number of individuals divided by males and females (F/M) collected for processing, individuals respectively. Table S2a Collection date of abiotic environmental variables and measured physical and chemical characteristics of 27 lochs, from left to right: mean depth (Mean depth), maximum depth (Max depth), surface area (area), pH, conductivity, salinity, composition of the margin substrate (proportions of mud, soft peat, hard peat, gravel, rock). Table S2b Water light spectra (orange ratio), chlorophyll A and chemical characteristics of 27 lochs. Table S3 Trait means (M) and standard deviations (SD) per population in mm (from left to right). Table S4 Loadings of landmarks on the first two PC axes and the percent variance explained by each axis. Table S5 Population‐level summary statistics for RAD sequences. Table S6 Loadings of morphological variables on the 10 PC axes and the percent variance explained by each axis. Table S7 Pearson correlation coefficients (r) among traits for all populations pooled together and each population separately. Table S8 Loadings of abiotic environmental variables on the axes of environmental variation and the percent variance explained by each axis. Table S9 Pearson correlation coefficients (r), P‐values and FDR corrected P‐values of correlations among abiotic environmental variables. Table S10 Summary statistics for the 18 populations from North Uist with RAD sequencing data as outputted by the program populations. Table S11 FST values based on whole genome among 18 freshwater populations of three‐spined stickleback from North Uist. [file MEC-25-4319-s001.docx]

The ecology of an adaptive radiation of three-spined stickleback from North Uist, Scotland

Isabel S. Magalhaes^1^, Daniele D’Agostino ^1^, Paul A. Hohenlohe^2^, and Andrew D. C. MacColl ^1^.

*^1^* *School of Life Sciences, University of Nottingham, University Park, Nottingham, NG7 2RD, U.K.*

*^2^ Institute for Bioinformatics and Evolutionary Studies, Department of Biological Sciences, University of Idaho, Moscow, ID 83844, USA.*

Table of contents for Supporting Information:

Figure S1. Neighbour joining tree …………………………………………….…………….…2

Table S1. Summary table for biotic environmental variables………………….….3

Table S2. Summary table for abiotic environmental variables………………..….4

Table S3. Summary table for phenotypic measurements ………………….……...7

Table S4. Loadings of landmarks on the first 2 PC axes of body shape……….9

Table S5. Population-level summary statistics for RAD sequences………….…10

Table S6. Loadings of morphological variables on the PC axes..…………………11

Table S7. Pearson correlations for phenotypic traits………………………………….12

Table S8. Loadings of environmental variables on the PC axes………..……....17

Table S9. Pearson correlations for environmental variables………………….….18

Table S10. POPULATIONS summary table………….…………………………………..…19

Table S11. F_ST_ values among lakes……………………………………………………………...20

Details of RDA analyses……………………………………………………………………….………21

Figure S1. Neighbour-Joining tree based on 9 135 genetic markers for 324 individuals from18 populations. Codes and colours on the tips of the branches represent individuals and populations respectively.


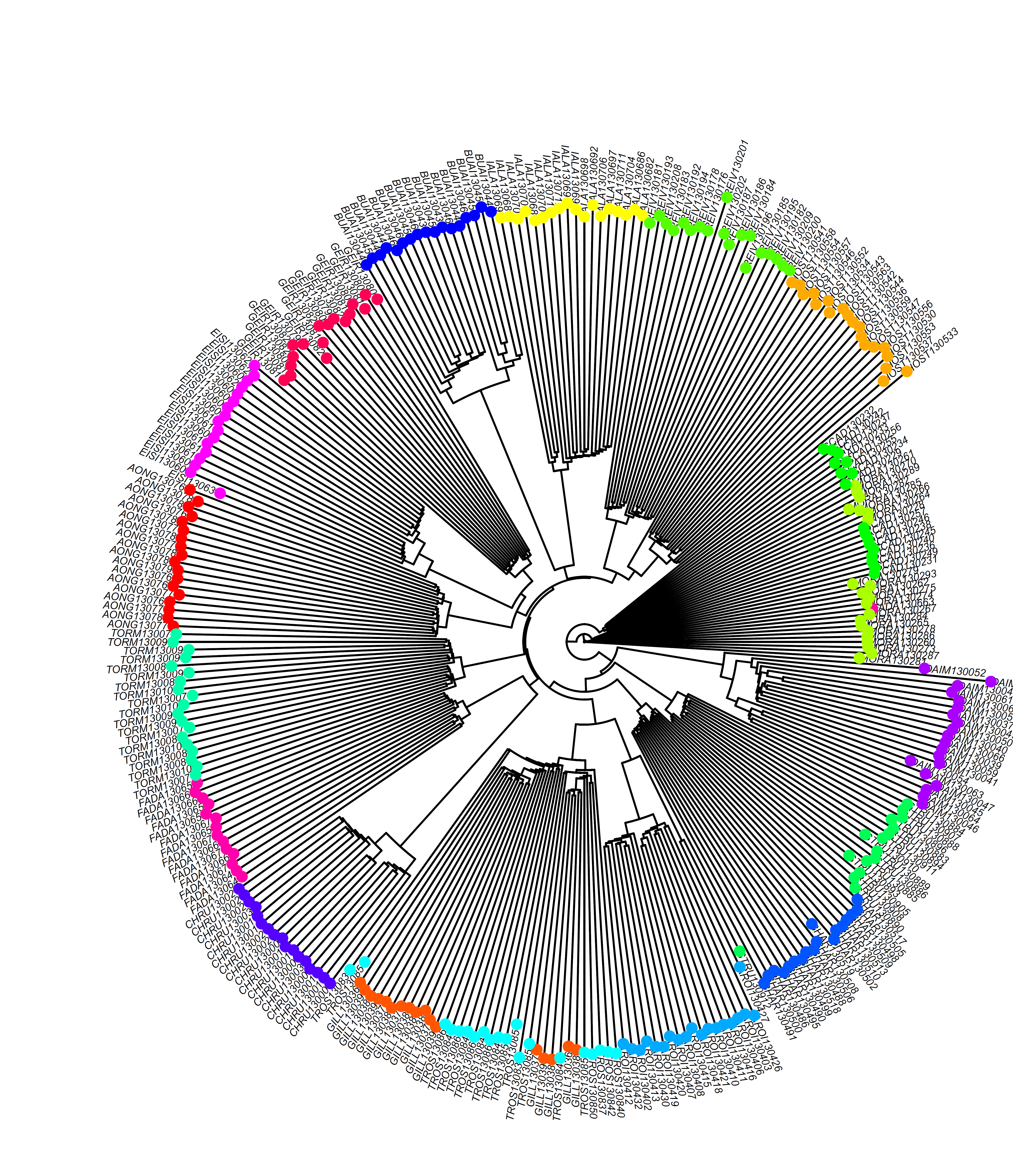


Table S1. Number of individuals divided by males and females (F/M) collected for processing, individuals respectively. catch per unit effort of three-spined stickleback (CPUE) and its standard deviation, and biotic environmental data (from left to right): average (M) and standard deviations (SD) for numbers of *Gyrodactylus* *arcuatus* (Gyro) and *Schistochephalus* spp. (Schisto) per population, absence (0) and presence (1) of trout and its catch rate (fish per angler per hour), absence (0) and presence (1) of *P. pungitius* and proportion of *P. pungitius* caught.

| loch | F/M | CPUE | CPUE | Gyro.M | Gyro.SD | Schisto | Schisto.SD | Trout | Trout | Pung. | %Pung. | Pung. |
| --- | --- | --- | --- | --- | --- | --- | --- | --- | --- | --- | --- | --- |
|  |  |  | SD |  |  |  |  |  | catch rate |  |  | SD |
| AONG | 28/6 | 12.68 | 6.96 | 0.23 | 0.5 | 0.25 | 0.74 | 1 | 0.25 | 0.00 | 0.00 | 0.00 |
| AROI | 17/17 | 0.79 | 0.39 | 2.91 | 4.11 | 0.03 | 0.17 | 1 | N/A | 1.00 | 0.49 | 0.27 |
| BHAR | 22/11 | 2.02 | 2.00 | 0.74 | 1.34 | 5.49 | 10.73 | 1 | 0.26 | 0.00 | 0.00 | 0.00 |
| BUAI | 24/9 | 9.76 | 15.08 | 0.03 | 0.17 | 0 | 0 | 0 | 0.00 | 0.00 | 0.00 | 0.00 |
| CHRU | 19/13 | 8.13 | 9.48 | 0 | 0 | 0.22 | 0.59 | 0 | 0.00 | 0.00 | 0.00 | 0.00 |
| CREI | 20/14 | 9.29 | 2.12 | 0.37 | 0.81 | 0 | 0 | 1 | N/A | 0.00 | 0.00 | N/A |
| DAIM | 12/21 | 2.45 | 1.16 | 0 | 0 | 0 | 0 | 1 | N/A | 1.00 | 0.15 | 0.03 |
| DUBH | 21/11 | 3.18 | 2.04 | 1.83 | 2.37 | 0 | 0 | 1 | 0.05 | 1.00 | 0.37 | 0.07 |
| EISI | 16/18 | 3.19 | 2.05 | 1.69 | 3.29 | 0.2 | 0.58 | 1 | 0.34 | 0.00 | 0.00 | 0.00 |
| FADA | 19/16 | 1.39 | 1.09 | 0.29 | 0.71 | 0 | 0 | 1 | 0.80 | 0.00 | 0.00 | 0.00 |
| FEIT | 24/9 | 7.12 | N/A | 3.69 | 2.91 | 0.06 | 0.24 | 1 | 0.18 | 1.00 | 0.60 | N/A |
| FHAI | 33/2 | 10.03 | 0.78 | 1.4 | 3.08 | 0.54 | 1.24 | 1 | 0.31 | 0.00 | 0.03 | 0.31 |
| GEIR | 20/14 | 6.65 | 1.40 | 2.2 | 3.1 | 0.11 | 0.53 | 1 | 0.12 | 0.00 | 0.00 | 0.00 |
| GILL | 20/11 | 0.97 | 0.33 | 6.31 | 6.44 | 0.17 | 0.47 | 1 | 0.30 | 1.00 | 0.18 | 0.09 |
| GROG | 25/9 | 12.07 | 7.17 | 0.37 | 0.69 | 0.14 | 0.43 | 1 | 0.13 | 1.00 | 0.04 | 0.04 |
| HOST | 16/18 | 10.84 | 10.79 | 4.6 | 4.99 | 1.57 | 1.36 | 1 | 0.16 | 1.00 | 0.28 | 0.13 |
| IALA | 28/7 | 11.25 | 5.25 | 0.14 | 0.36 | 0 | 0 | 0 | 0.00 | 0.00 | 0.00 | 0.00 |
| MAGA | 21/14 | 1.84 | 4.11 | 1.91 | 2.17 | 0 | 0 | 1 | 0.20 | 1.00 | 0.64 | 0.25 |
| MAIG | 23/12 | 1.66 | 1.54 | 0.51 | 0.78 | 0.03 | 0.17 | 1 | 0.57 | 1.00 | 0.02 | 0.02 |
| MORA | 8/22 | 1.44 | 0.98 | 0.29 | 0.52 | 0 | 0 | 1 | 2.29 | 1.00 | 0.01 | 0.06 |
| NAGE | 16/19 | 2.79 | 1.71 | 2.83 | 4.16 | 0.89 | 2.33 | 1 | N/A | 0.00 | 0.01 | 0.01 |
| REIV | 17/18 | 42.79 | 42.76 | 1.17 | 1.76 | 0.06 | 0.24 | 0 | 0.00 | 1.00 | 0.19 | 0.23 |
| SAND | 32/3 | 12.28 | N/A | 1.69 | 1.89 | 0.03 | 0.17 | 1 | 0.20 | 1.00 | 0.07 | N/A |
| SCAD | 24/11 | 30.43 | 28.41 | 1.29 | 1.78 | 0.09 | 0.28 | 1 | 1.29 | 1.00 | 0.04 | 0.02 |
| STRU | 22/13 | 15.75 | 14.82 | 2.86 | 6.38 | 1.63 | 3.37 | 1 | 0.51 | 0.00 | 0.00 | 0.00 |
| TORM | 13/17 | 16.88 | 32.45 | 0.57 | 1.09 | 0.09 | 0.51 | 1 | 0.25 | 0.00 | 0.00 | 0.00 |
| TROS | 18/15 | 1.9 | N/A | 1.14 | 1.31 | 0.03 | 0.17 | 1 | 0.10 | 1.00 | 0.27 | N/A |

Table S2. Collection date of abiotic environmental variables and measured physical and chemical characteristics of 27 lochs, from left to right: mean depth (Mean depth), maximum depth (Max depth), surface area (area), pH, conductivity, salinity, composition of the margin substrate (proportions of mud, soft peat, hard peat, gravel, rock). Information on surface area, average depth and maximum depth is partly based on Murray & Pullar 1910. The values presented were measured in 2013, standard deviations for pH and conductivity were estimated from 2 to 8 point measurements done over 6 years (2006-2011).

| Lake | Date | Mean | Max depth | Area(m2) | pH | pH  SD | Cond. | Cond.  SD | Salinity | Mud | Soft | Hard peat | Sand | Gravel | Rock |
| --- | --- | --- | --- | --- | --- | --- | --- | --- | --- | --- | --- | --- | --- | --- | --- |
|  |  | Depth (m) | (m) |  |  |  | (uS/cm) |  |  |  | peat |  |  |  |  |
| AONG | 16.05.13 | 1.017 | 1.4 | 95822 | 6.845 | 0.21 | 206.84 | 41.54 | 98.00 | 0.08 | 0.02 | 0.18 | 0.54 | 0.06 | 0.14 |
| AROI | 09.05.13 | 0.913 | 1.8 | 65416 | 6.39 | 0.37 | 202.40 | 30.84 | 116.00 | 0.00 | 0.00 | 0.00 | 0.50 | 0.11 | 0.41 |
| BHAR | 14.05.13 | 4.138 | 11.278 | 538536 | 6.005 | 0.19 | 147.60 | 8.54 | 76.00 | 0.45 | 0.06 | 0.04 | 0.00 | 0.06 | 0.40 |
| BUAI | 13.05.13 | 0.842 | 1.2 | 16516 | 6.73 | 0.26 | 212.00 | 35.86 | 102.00 | 0.46 | 0.11 | 0.15 | 0.00 | 0.02 | 0.27 |
| CHRU | 29.04.13 | 0.815 | 1.2 | 21309 | 6.46 | 0.37 | 165.50 | 23.47 | 91.00 | 0.00 | 0.15 | 0.30 | 0.27 | 0.05 | 0.24 |
| CREI | 16.05.13 | 0.819 | 1.5 | 15982 | 6.765 | 0.43 | 202.00 | 38.04 | 105.00 | 0.03 | 0.10 | 0.53 | 0.09 | 0.00 | 0.25 |
| DAIM | 30.04.13 | 1.242 | 1.8 | 36066 | 6.367 | 0.22 | 191.88 | 31.31 | 110.00 | 0.00 | 0.05 | 0.12 | 0.17 | 0.09 | 0.58 |
| DUBH | 02.05.13 | 1.358 | 2.9 | 226363 | 6.907 | 0.05 | 202.75 | 33.72 | 116.00 | 0.00 | 0.42 | 0.22 | 0.06 | 0.02 | 0.30 |
| EISI | 17.05.13 | 1.105 | 1.8 | 114387 | 6.77 | 0.17 | 157.50 | 31.77 | 185.00 | 0.05 | 0.00 | 0.00 | 0.28 | 0.24 | 0.44 |
| FADA | 17.05.13 | 0.903 | 1.9 | 106000 | 6.67 | 0.32 | 144.25 | 15.49 | 75.00 | 0.18 | 0.00 | 0.08 | 0.25 | 0.13 | 0.36 |
| FEIT | 05.06.13 | 0.64 | 1 | 156872 | 8.705 | 0.45 | 470.50 | 30.45 | 224.00 | 0.57 | 0.00 | 0.00 | 0.26 | 0.04 | 0.13 |
| FHAI | 17.05.13 | 0.961 | 1.9 | 80268 | 6.415 | 0.18 | 177.00 | 35.89 | 107.00 | 0.00 | 0.06 | 0.04 | 0.22 | 0.13 | 0.56 |
| GEIR | 16.05.13 | 2.148 | 5.486 | 1881837 | 6.5 | 0.40 | 149.44 | 21.41 | 81.00 | 0.14 | 0.00 | 0.05 | 0.26 | 0.03 | 0.53 |
| GILL | 09.05.13 | 1.014 | 1.6 | 140597 | 6.7 | 0.16 | 182.25 | 25.66 | 103.00 | 0.00 | 0.00 | 0.02 | 0.41 | 0.25 | 0.33 |
| GROG | 19.05.13 | 0.687 | 1 | 148368 | 8.05 | 0.28 | 373.50 | 51.91 | 202.00 | 0.11 | 0.00 | 0.00 | 0.37 | 0.12 | 0.41 |
| HOST | 15.05.13 | 4.243 | 9.449 | 258301 | 8.26 | 0.18 | 360.75 | 33.02 | 192.00 | 0.05 | 0.00 | 0.00 | 0.63 | 0.12 | 0.20 |
| IALA | 16.05.13 | 0.411 | 0.75 | 4581 | 6.39 | 0.09 | 207.50 | 48.15 | 87.00 | 0.63 | 0.23 | 0.12 | 0.00 | 0.00 | 0.02 |
| MAGA | 03.05.13 | 0.798 | 1.3 | 64600 | 7.675 | 0.21 | 283.88 | 42.78 | 153.00 | 0.00 | 0.00 | 0.00 | 0.45 | 0.02 | 0.53 |
| MAIG | 08.05.13 | 3.401 | 13.4 | 96070 | 6.73 | 0.23 | 169.75 | 57.77 | 88.00 | 0.00 | 0.09 | 0.06 | 0.17 | 0.26 | 0.44 |
| MORA | 07.05.13 | 2.563 | 6.096 | 378284 | 6.525 | 0.52 | 179.28 | 14.57 | 91.00 | 0.00 | 0.00 | 0.02 | 0.09 | 0.26 | 0.64 |
| NAGE | 16.05.13 | 0.808 | 1.3 | 74404 | 7.315 | 0.40 | 290.00 | 48.57 | 150.00 | 0.44 | 0.00 | 0.00 | 0.11 | 0.13 | 0.32 |
| REIV | 04.05.13 | 0.742 | 1 | 60999 | 8.655 | 0.30 | 504.13 | 69.62 | 287.00 | 0.35 | 0.00 | 0.00 | 0.47 | 0.00 | 0.19 |
| SAND | 19.05.13 | 0.766 | 1.7 | 155286 | 8.205 | 0.05 | 412.75 | 48.69 | 226.00 | 0.00 | 0.00 | 0.00 | 0.91 | 0.04 | 0.05 |
| SCAD | 06.05.13 | 3.048 | 15.24 | 5516207 | 6.187 | 0.29 | 145.70 | 12.20 | 73.00 | 0.00 | 0.12 | 0.11 | 0.11 | 0.01 | 0.65 |
| STRU | 17.05.13 | 1.284 | 2.7 | 192399 | 6.88 | 0.20 | 187.25 | 32.36 | 106.00 | 0.08 | 0.10 | 0.08 | 0.20 | 0.11 | 0.44 |
| TORM | 01.05.13 | 1.887 | 3.048 | 210793 | 6.695 | 0.19 | 195.20 | 24.45 | 104.00 | 0.00 | 0.05 | 0.05 | 0.30 | 0.07 | 0.53 |
| TROS | 19.05.13 | 0.89 | 1.5 | 65404 | 6.56 | 0.21 | 192.50 | 27.58 | 110.00 | 0.11 | 0.00 | 0.00 | 0.50 | 0.09 | 0.31 |

Table S2b. Water light spectra (orange ratio), chlorophyll A and chemical characteristics of 27 lochs.

|  |  |  |  |  |  |  |  |  |  |  |  |  |  |
| --- | --- | --- | --- | --- | --- | --- | --- | --- | --- | --- | --- | --- | --- |
|  | Orange | ChlA |  |  |  |  |  |  |  |  |  |  |  |
| **loch** | ratio | (ug/L) | Cu(µg/L) | Zn(µg/L) | Cd(µg/L) | Mn (µg/L) | Pb (µg/L) | Na(mg/L) | Ca(mg/L) | Mg(mg/L) | K(mg/L) | Sulphate | DOC |
| **AONG** | 0.399 | 0.913 | 3.63E-11 | 8.74E-07 | 6.57E-11 | 9.49E-08 | 5.99E-12 | 0.000824 | 0.000102 | 0.000107 | 1.86E-05 | 0.808 | 14.25 |
| **AROI** | 0.36 | 1.082 | 2.77E-12 | 4.87E-07 | 2.83E-11 | 1.99E-07 | 8.26E-12 | 0.001048 | 6.54E-05 | 0.000124 | 2.42E-05 | 0.823 | 12.8 |
| **BHAR** | 0.315 | 0.847 | 3.28E-11 | 9.24E-07 | 2.24E-11 | 1.79E-07 | 5.31E-11 | 0.000804 | 3.42E-05 | 9.78E-05 | 2.11E-05 | 1.31 | 7.067 |
| **BUAI** | 0.427 | 1.726 | 1.97E-11 | 6.56E-07 | 3.69E-11 | 3.35E-08 | 1.44E-11 | 0.001565 | 6.01E-05 | 0.000122 | 2.56E-05 | 1.423 | 6.84 |
| **CHRU** | 0.323 | 2.827 | 2.63E-12 | 5.96E-07 | 3.69E-11 | 1.53E-07 | 1.45E-11 | 0.000808 | 5.00E-05 | 9.95E-05 | 1.40E-05 | 1.485 | 9.373 |
| **CREI** | 0.322 | 0.503 | 1.38E-11 | 4.99E-07 | 2.38E-11 | 6.50E-08 | 6.67E-12 | 0.00113 | 7.63E-05 | 0.00011 | 3.07E-05 | 0.609 | 13.67 |
| **DAIM** | 0.431 | 0.879 | 1.20E-11 | 4.89E-07 | 2.90E-11 | 7.58E-08 | 1.53E-11 | 0.001059 | 5.20E-05 | 1.00E-04 | 2.29E-05 | 0.635 | 11.47 |
| **DUBH** | 0.368 | 0.629 | 1.40E-09 | 7.29E-07 | 3.26E-11 | 4.84E-08 | 4.39E-12 | 0.001023 | 6.45E-05 | 0.000133 | 3.13E-05 | 0.512 | 11.16 |
| **EISI** | 0.454 | 0.076 | 1.55E-10 | 7.45E-07 | 2.80E-11 | 6.79E-08 | 2.93E-12 | 0.000974 | 6.50E-05 | 0.000122 | 2.53E-05 | 1 | 8.457 |
| **FADA** | 0.364 | 0.155 | 5.04E-12 | 7.36E-07 | 1.17E-11 | 2.06E-08 | 1.51E-12 | 0.000745 | 4.06E-05 | 9.13E-05 | 2.01E-05 | 0.59 | 9.115 |
| **FEIT** | 0.697 | 1.556 | 3.59E-14 | 6.87E-08 | 5.02E-12 | 2.73E-09 | 1.80E-15 | 0.001816 | 0.000776 | 0.00022 | 4.37E-05 | 0.993 | 9.181 |
| **FHAI** | 0.394 | 0.733 | 1.43E-12 | 4.08E-07 | 2.36E-11 | 9.09E-08 | 3.23E-12 | 0.001023 | 5.77E-05 | 0.000123 | 2.77E-05 | 0.991 | 9.551 |
| **GEIR** | 0.175 | 0.249 | 3.94E-12 | 4.47E-07 | 1.81E-11 | 1.90E-07 | 1.04E-11 | 0.00074 | 4.01E-05 | 9.16E-05 | 2.13E-05 | 1.137 | 8.066 |
| **GILL** | 0.49 | 0.456 | 5.93E-12 | 3.69E-07 | 1.86E-11 | 7.64E-08 | 5.77E-12 | 0.000941 | 6.04E-05 | 0.000122 | 2.90E-05 | 0.842 | 13.77 |
| **GROG** | 0.468 | 1.394 | 2.33E-13 | 2.98E-07 | 1.16E-11 | 2.45E-08 | 2.26E-14 | 0.001602 | 0.000638 | 0.000234 | 6.08E-05 | 1.512 | 12.35 |
| **HOST** | 0.729 | 0.624 | 3.23E-13 | 3.07E-07 | 6.77E-12 | 5.56E-08 | 2.23E-14 | 0.001193 | 0.000723 | 0.000196 | 4.94E-05 | 0.706 | 7.426 |
| **IALA** | 0.351 | 1.597 | 3.08E-11 | 5.27E-07 | 1.52E-11 | 1.09E-07 | 8.23E-12 | 0.000918 | 5.95E-05 | 9.72E-05 | 2.34E-05 | 0.962 | 15.9 |
| **MAGA** | 0.517 | 0.407 | 1.70E-12 | 5.15E-07 | 1.79E-11 | 3.67E-07 | 2.76E-13 | 0.001478 | 0.000291 | 0.000184 | 3.50E-05 | 1.36 | 13.25 |
| **MAIG** | 0.457 | 0.155 | 1.21E-09 | 7.75E-07 | 2.50E-11 | 3.60E-08 | 1.19E-12 | 0.001051 | 7.70E-05 | 0.000109 | 2.37E-05 | 1.487 | 7.402 |
| **MORA** | 0.488 | 0.579 | 4.36E-11 | 6.14E-07 | 1.53E-11 | 9.96E-08 | 9.42E-12 | 0.001038 | 5.83E-05 | 0.000111 | 2.85E-05 | 0.837 | 8.9 |
| **NAGE** | 0.661 | 0.779 | 5.97E-12 | 8.29E-07 | 3.07E-11 | 1.09E-07 | 1.76E-12 | 0.001438 | 8.71E-05 | 0.000148 | 2.64E-05 | 1.209 | 8.877 |
| **REIV** | 0.609 | 6.349 | 5.97E-15 | 2.96E-08 | 1.41E-12 | 8.51E-10 | 1.26E-15 | 0.001746 | 0.000449 | 0.000186 | 7.15E-05 | 2.001 | 9.819 |
| **SAND** | 0.599 | 0.459 | 4.67E-13 | 3.51E-07 | 3.38E-12 | 2.01E-08 | 5.80E-14 | 0.001552 | 0.000752 | 0.000211 | 5.66E-05 | 1.317 | 9.919 |
| **SCAD** | 0.235 | 0.436 | 2.03E-11 | 8.37E-07 | 3.17E-11 | 1.53E-07 | 2.19E-11 | 0.000853 | 3.27E-05 | 9.58E-05 | 2.11E-05 | 1.379 | 6.97 |
| **STRU** | 0.592 | 0.513 | 1.97E-12 | 6.86E-07 | 1.75E-11 | 6.13E-08 | 6.38E-13 | 0.001081 | 8.45E-05 | 0.000127 | 2.19E-05 | 0.777 | 10.83 |
| **TORM** | 0.228 | 0.816 | 7.89E-10 | 7.77E-07 | 2.25E-11 | 3.50E-08 | 6.09E-13 | 0.001099 | 9.19E-05 | 0.00013 | 2.70E-05 | 1.618 | 9.361 |
| **TROS** | 0.356 | 0.21 | 4.42E-11 | 9.53E-07 | 2.01E-11 | 2.61E-07 | 9.86E-12 | 0.001129 | 6.60E-05 | 0.000138 | 3.57E-05 | 1.132 | 8.855 |

Table S3. Trait means (M) and standard deviations (SD) per population in mm (from left to right): standard length (SL), number of armour plates (n. plates), length of 1^st^ dorsal spine (DS1) and 2nd dorsal spine (DS2), length of biggest armour plate (BAP), length of the ascending process of the pelvis (LP) and height of the ascending process of the pelvis (HP), length of pelvic spine (PS) and first two PC scores for body shape (bodyPC).

| loch | SL(mm)  M | SL(mm)  SD | n.plate  M | n.plate  SD | DS1  M | DS1  SD | DS2  M | DS2  SD | BAP.M | BAP.SD | LP.M | LP.SD | HP.M | HP.SD | PS.M | PS.SD | bodyPC1  M | bodyPC1  SD | bodyPC2  M | bodyPC2  SD |
| --- | --- | --- | --- | --- | --- | --- | --- | --- | --- | --- | --- | --- | --- | --- | --- | --- | --- | --- | --- | --- |
| AONG | 28.621 | 4.082 | 3.118 | 0.729 | -0.034 | 0.025 | -0.030 | 0.022 | -0.005 | 0.050 | 0.006 | 0.022 | 0.017 | 0.035 | -0.043 | 0.029 | -0.006 | 0.009 | -0.004 | 0.008 |
| AROI | 42.894 | 5.859 | 3.941 | 0.547 | 0.047 | 0.035 | 0.032 | 0.045 | 0.012 | 0.050 | 0.001 | 0.031 | 0.044 | 0.037 | 0.095 | 0.051 | -0.018 | 0.010 | 0.005 | 0.011 |
| BHAR | 28.139 | 4.384 | 0.333 | 0.854 | -0.041 | 0.043 | -0.169 | 0.074 | -0.254 | 0.085 | -0.034 | 0.021 | -0.285 | 0.075 | -0.282 | 0.072 | -0.015 | 0.009 | 0.001 | 0.010 |
| BUAI | 33.421 | 3.460 | 3.242 | 0.561 | 0.017 | 0.018 | 0.017 | 0.018 | 0.022 | 0.039 | -0.022 | 0.061 | -0.077 | 0.159 | -0.162 | 0.153 | 0.035 | 0.006 | 0.003 | 0.010 |
| CHRU | 30.600 | 6.835 | 3.781 | 0.491 | 0.002 | 0.019 | -0.005 | 0.019 | 0.023 | 0.043 | 0.006 | 0.033 | -0.024 | 0.057 | -0.010 | 0.039 | 0.030 | 0.009 | 0.008 | 0.013 |
| CREI | 28.456 | 3.932 | 3.500 | 0.826 | 0.020 | 0.027 | 0.023 | 0.026 | 0.016 | 0.052 | 0.014 | 0.023 | 0.008 | 0.028 | 0.021 | 0.040 | 0.009 | 0.009 | -0.001 | 0.012 |
| DAIM | 32.091 | 2.699 | 3.212 | 0.781 | -0.021 | 0.055 | -0.006 | 0.021 | -0.028 | 0.047 | 0.002 | 0.020 | -0.032 | 0.032 | -0.024 | 0.033 | -0.009 | 0.007 | 0.009 | 0.014 |
| DUBH | 33.584 | 6.225 | 5.094 | 0.734 | 0.010 | 0.025 | 0.008 | 0.022 | 0.044 | 0.038 | 0.017 | 0.025 | 0.020 | 0.044 | 0.025 | 0.032 | -0.005 | 0.009 | 0.010 | 0.009 |
| EISI | 32.006 | 5.544 | 5.235 | 1.156 | 0.009 | 0.028 | 0.002 | 0.028 | 0.019 | 0.043 | 0.005 | 0.025 | 0.000 | 0.034 | -0.010 | 0.043 | 0.005 | 0.010 | -0.001 | 0.011 |
| FADA | 27.840 | 3.585 | 0.229 | 0.770 | -0.143 | 0.057 | -0.146 | 0.117 | -0.266 | 0.058 | -0.185 | 0.027 | -0.300 | 0.049 | -0.287 | 0.046 | -0.015 | 0.009 | -0.006 | 0.013 |
| FEIT | 39.315 | 7.948 | 4.242 | 0.708 | 0.016 | 0.044 | 0.011 | 0.038 | 0.009 | 0.060 | 0.003 | 0.034 | 0.014 | 0.050 | 0.002 | 0.054 | 0.011 | 0.008 | -0.003 | 0.009 |
| FHAI | 32.786 | 5.077 | 2.286 | 1.341 | -0.051 | 0.035 | -0.048 | 0.037 | -0.233 | 0.088 | -0.014 | 0.029 | -0.125 | 0.069 | -0.079 | 0.051 | 0.008 | 0.010 | -0.011 | 0.007 |
| GEIR | 29.253 | 4.025 | 1.647 | 1.475 | -0.021 | 0.027 | -0.023 | 0.021 | -0.124 | 0.135 | 0.009 | 0.017 | 0.014 | 0.027 | -0.037 | 0.029 | -0.008 | 0.011 | -0.003 | 0.010 |
| GILL | 36.865 | 9.189 | 4.290 | 0.643 | 0.080 | 0.040 | 0.081 | 0.034 | 0.065 | 0.053 | 0.031 | 0.042 | 0.059 | 0.049 | 0.090 | 0.055 | -0.006 | 0.010 | -0.002 | 0.009 |
| GROG | 35.476 | 5.174 | 4.412 | 1.104 | 0.019 | 0.045 | 0.023 | 0.031 | 0.005 | 0.050 | 0.010 | 0.020 | 0.030 | 0.032 | 0.033 | 0.059 | 0.000 | 0.008 | -0.013 | 0.009 |
| HOST | 37.065 | 6.634 | 4.588 | 0.857 | 0.028 | 0.036 | 0.023 | 0.047 | 0.000 | 0.048 | 0.011 | 0.022 | 0.028 | 0.042 | 0.034 | 0.046 | -0.002 | 0.011 | -0.008 | 0.012 |
| IALA | 35.143 | 5.483 | 4.371 | 0.770 | -0.010 | 0.025 | -0.010 | 0.025 | 0.067 | 0.049 | 0.015 | 0.031 | 0.021 | 0.041 | -0.031 | 0.048 | 0.022 | 0.008 | 0.006 | 0.010 |
| MAGA | 39.320 | 5.975 | 4.543 | 0.852 | 0.022 | 0.030 | 0.020 | 0.030 | 0.007 | 0.052 | 0.011 | 0.024 | 0.011 | 0.043 | 0.038 | 0.048 | 0.003 | 0.008 | -0.001 | 0.012 |
| MAIG | 31.114 | 3.492 | 0.086 | 0.373 | -0.121 | 0.074 | -0.211 | 0.052 | -0.315 | 0.058 | -0.046 | 0.023 | -0.331 | 0.052 | -0.328 | 0.044 | -0.017 | 0.007 | 0.001 | 0.012 |
| MORA | 31.020 | 2.752 | 0.033 | 0.183 | -0.178 | 0.041 | -0.201 | 0.047 | -0.315 | 0.046 | -0.063 | 0.023 | -0.343 | 0.037 | -0.327 | 0.035 | -0.034 | 0.008 | 0.003 | 0.011 |
| NAGE | 31.457 | 5.149 | 4.343 | 0.873 | 0.007 | 0.028 | -0.011 | 0.043 | 0.030 | 0.029 | 0.001 | 0.020 | 0.028 | 0.030 | 0.004 | 0.027 | 0.005 | 0.008 | -0.002 | 0.011 |
| REIV | 38.543 | 8.436 | 5.486 | 1.222 | 0.009 | 0.042 | 0.010 | 0.035 | 0.067 | 0.038 | 0.033 | 0.029 | 0.065 | 0.041 | 0.034 | 0.060 | 0.024 | 0.010 | 0.008 | 0.011 |
| SAND | 38.717 | 7.992 | 3.371 | 0.770 | -0.037 | 0.046 | -0.016 | 0.076 | 0.013 | 0.050 | 0.011 | 0.026 | -0.004 | 0.053 | -0.054 | 0.067 | 0.004 | 0.007 | -0.014 | 0.010 |
| SCAD | 31.820 | 5.406 | 0.143 | 0.494 | -0.147 | 0.082 | -0.137 | 0.079 | -0.325 | 0.089 | -0.059 | 0.034 | -0.352 | 0.073 | -0.337 | 0.069 | -0.023 | 0.009 | 0.006 | 0.012 |
| STRU | 31.054 | 5.901 | 0.343 | 0.802 | -0.032 | 0.047 | -0.033 | 0.045 | -0.293 | 0.120 | 0.005 | 0.030 | -0.051 | 0.060 | -0.040 | 0.056 | 0.009 | 0.009 | -0.002 | 0.012 |
| TORM | 29.467 | 3.573 | 0.233 | 0.568 | -0.079 | 0.066 | -0.134 | 0.072 | -0.273 | 0.055 | -0.007 | 0.018 | -0.178 | 0.146 | -0.086 | 0.036 | -0.007 | 0.010 | 0.007 | 0.011 |
| TROS | 39.648 | 5.817 | 4.030 | 0.585 | 0.072 | 0.029 | 0.066 | 0.031 | 0.044 | 0.046 | 0.021 | 0.043 | 0.072 | 0.025 | 0.077 | 0.061 | -0.007 | 0.009 | 0.005 | 0.010 |

Table S4.Loadings of landmarks on the first two PC axes and the percent variance explained by each axis. The highest loadings on each PC are in bold.

|  | bodyPC1 | bodyPC2 |
| --- | --- | --- |
| x1 | -0.0968 | -0.0725 |
| y1 | -0.0508 | -0.0894 |
| x2 | -0.1075 | -0.2687 |
| y2 | 0.0240 | -0.0149 |
| x3 | **0.5152** | -0.2656 |
| y3 | -0.0090 | 0.1693 |
| x4 | 0.1936 | **0.3838** |
| y4 | -0.0051 | -0.0385 |
| x5 | **-0.3336** | -0.1559 |
| y5 | 0.0383 | 0.0408 |
| x6 | -0.3151 | -0.1132 |
| y6 | 0.0123 | -0.0012 |
| x7 | **-0.3454** | -0.1519 |
| y7 | 0.0031 | -0.0420 |
| x8 | 0.1858 | **0.3612** |
| y8 | 0.0448 | 0.0048 |
| x9 | **0.4967** | **-0.4032** |
| y9 | -0.0869 | -0.1333 |
| x10 | -0.1008 | **0.4194** |
| y10 | -0.1295 | -0.1391 |
| x11 | -0.0801 | -0.0545 |
| y11 | 0.0233 | 0.0317 |
| x12 | -0.0145 | 0.1021 |
| y12 | 0.0076 | 0.0291 |
| x13 | 0.0026 | 0.2191 |
| y13 | 0.1281 | 0.1828 |
| % variance explained | 34.2 | 16.5 |

Table S5. Population-level summary statistics for RAD sequences. From left to right: number of individuals analysed per population , average individual total number of reads (Total) and retained reads after quality controls (Retained), average number of reads that were aligned to the reference genome by GSnap (GSnap Aligned) and percentage of the retained reads those aligned reads represent (% Aligned), number of aligned reads that were used in the Stacks analyses (Used by Stacks) and percentage of the aligned reads those reads represent (% used by Stacks), and mean individual coverage depth (Mean Coverage Depth).

| POP | N | Total | Retained | GSnap aligned | % Aligned | Used by Stacks | % used by Stacks | Mean Coverage Depth |
| --- | --- | --- | --- | --- | --- | --- | --- | --- |
| AONG | 18 | 1169374 | 1135693 | 990305 | 87.19 | 990305 | 100.0 | 25.20942 |
| AROI | 19 | 1115062 | 1080725 | 929302 | 85.97 | 929302 | 100.0 | 23.60154 |
| AROI | 17 | 1275770 | 1240482 | 1079111 | 87.00 | 1079111 | 100.0 | 27.23649 |
| BUAI | 18 | 1072651 | 1053387 | 922307 | 87.57 | 922307 | 100.0 | 24.03681 |
| CHRU | 17 | 1074909 | 1048642 | 913812 | 87.06 | 913812 | 100.0 | 23.51634 |
| DAIM | 20 | 1182563 | 1154506 | 1002716 | 86.83 | 1002716 | 100.0 | 25.36071 |
| EISI | 17 | 1188532 | 1151336 | 990814 | 85.98 | 990814 | 100.0 | 25.18494 |
| EISI | 15 | 1263095 | 1229761 | 1065986 | 86.69 | 1065986 | 100.0 | 27.34407 |
| GEIR | 17 | 1034467 | 997840 | 869490 | 87.12 | 869490 | 100.0 | 22.34513 |
| GILL | 17 | 964687 | 943364 | 817401 | 86.58 | 817401 | 100.0 | 21.30855 |
| HOST | 19 | 1119894 | 1099674 | 936889 | 85.22 | 936889 | 100.0 | 23.47165 |
| IALA | 18 | 1150907 | 1122633 | 967195 | 86.18 | 967195 | 100.0 | 24.42878 |
| MORA | 21 | 1098546 | 1075007 | 924764 | 86.02 | 924764 | 100.0 | 23.17826 |
| OBSE | 18 | 1290998 | 1270293 | 1097402 | 86.39 | 1097402 | 100.0 | 27.14613 |
| OBSM | 16 | 1376643 | 1353280 | 1164720 | 85.99 | 1164720 | 100.0 | 28.66981 |
| REIV | 19 | 1302291 | 1278813 | 1098901 | 85.85 | 1098901 | 100.0 | 27.12813 |
| SCAD | 17 | 1051377 | 1028638 | 888367 | 86.39 | 888367 | 100.0 | 22.26045 |
| STRU | 16 | 1076197 | 1047285 | 900156 | 85.95 | 900156 | 100.0 | 22.52909 |
| TORM | 19 | 1095031 | 1069661 | 916790 | 85.73 | 916790 | 100.0 | 23.13811 |
| TROS | 20 | 1169343 | 1138749 | 978741 | 85.87 | 978741 | 100.0 | 24.59232 |

Table S6. Loadings of morphological variables on the 10 PC axes and the percent variance explained by each axis. The highest loadings on each of the first 5 PCs are in bold.

|  | PC1 | PC2 | PC3 | PC4 | PC5 | PC6 | PC7 | PC8 | PC9 | PC10 |
| --- | --- | --- | --- | --- | --- | --- | --- | --- | --- | --- |
| SL | 0.102 | **0.761** | -0.547 | 0.047 | -0.216 | 0.203 | -0.056 | 0.010 | -0.131 | -0.019 |
| bodyPC1 | 0.229 | -0.065 | 0.008 | **-0.929** | -0.224 | 0.150 | 0.003 | -0.049 | 0.037 | -0.056 |
| bodyPC2 | 0.007 | 0.569 | **0.813** | -0.016 | -0.006 | 0.085 | 0.068 | -0.030 | 0.018 | 0.036 |
| n.plate | **0.356** | 0.219 | -0.111 | -0.081 | 0.331 | -0.542 | -0.083 | 0.020 | 0.613 | 0.146 |
| DS1 | **0.360** | -0.113 | 0.101 | 0.127 | 0.097 | 0.389 | -0.809 | 0.001 | 0.018 | 0.126 |
| DS2 | **0.369** | -0.074 | -0.009 | 0.079 | 0.201 | 0.412 | 0.425 | 0.666 | 0.092 | 0.090 |
| BAP | **0.378** | 0.048 | 0.041 | -0.068 | 0.337 | -0.383 | -0.049 | 0.107 | -0.697 | -0.299 |
| LP | **0.308** | -0.105 | 0.121 | 0.204 | **-0.792** | -0.350 | -0.064 | 0.290 | 0.008 | -0.005 |
| HP | **0.394** | -0.085 | -0.015 | 0.100 | -0.046 | 0.039 | 0.310 | -0.505 | -0.224 | 0.650 |
| PS | **0.389** | -0.073 | 0.019 | 0.221 | -0.047 | 0.211 | 0.217 | -0.448 | 0.246 | -0.662 |
|  |  |  |  |  |  |  |  |  |  |  |
| St. deviation | 2.354 | 1.014 | 1.000 | 0.878 | 0.747 | 0.617 | 0.527 | 0.461 | 0.382 | 0.286 |
| Variance explained | 0.554 | 0.103 | 0.100 | 0.077 | 0.056 | 0.038 | 0.028 | 0.021 | 0.015 | 0.008 |
| Cumulative % variance explained | 0.554 | 0.657 | 0.757 | 0.834 | 0.890 | 0.928 | 0.956 | 0.977 | 0.992 | 1.000 |

Table S7. Pearson correlation coefficients (r) among traits for all populations pooled together and each population separately. P-values were FDR corrected for multiple testing. Significant values are in bold.

|  |  | AONG |  | AROI |  | BHAR |  | BUAI |  | CHRU |  | CREI |  | DAIM |  | DUBH |  |
| --- | --- | --- | --- | --- | --- | --- | --- | --- | --- | --- | --- | --- | --- | --- | --- | --- | --- |
| trait1 | trait2 | r | p-value | r | p-value | r | p-value | r | p-value | r | p-value | r | p-value | r | p-value | r | p-value |
| SL | n.plate | 0.33 | 0.16 | 0.11 | 0.91 | -0.06 | 0.80 | -0.40 | 0.15 | -0.22 | 0.30 | 0.24 | 0.37 | -0.05 | 0.84 | 0.14 | 0.82 |
| SL | bodyPC1 | 0.02 | 0.96 | 0.19 | 0.90 | -0.05 | 0.84 | 0.24 | 0.42 | -0.34 | 0.10 | 0.19 | 0.50 | -0.16 | 0.64 | **0.47** | **0.04** |
| SL | bodyPC2 | -0.17 | 0.67 | 0.06 | 0.95 | -0.18 | 0.46 | -0.13 | 0.62 | 0.39 | 0.06 | -0.14 | 0.65 | -0.14 | 0.64 | 0.24 | 0.44 |
| SL | DS1 | -0.38 | 0.12 | -0.24 | 0.78 | **-0.49** | **0.01** | -0.20 | 0.48 | **-0.63** | **0.00** | -0.44 | 0.06 | -0.30 | 0.54 | **-0.59** | **0.01** |
| SL | DS2 | -0.34 | 0.16 | -0.18 | 0.90 | **-0.70** | **0.00** | 0.06 | 0.79 | **-0.59** | **0.00** | -0.27 | 0.29 | -0.18 | 0.64 | **-0.64** | **0.00** |
| SL | BAP | -0.17 | 0.67 | -0.15 | 0.91 | **-0.83** | **0.00** | -0.39 | 0.15 | **-0.60** | **0.00** | **-0.46** | **0.04** | -0.31 | 0.54 | -0.17 | 0.77 |
| SL | LP | 0.04 | 0.96 | -0.01 | 0.96 | -0.39 | 0.07 | 0.17 | 0.57 | **-0.66** | **0.00** | -0.42 | 0.06 | -0.16 | 0.64 | 0.00 | 0.99 |
| SL | HP | -0.71 | **0.00** | -0.30 | 0.66 | **-0.67** | **0.00** | 0.30 | 0.25 | **-0.60** | **0.00** | -0.29 | 0.27 | -0.45 | 0.14 | **-0.55** | **0.01** |
| SL | PS | -0.56 | **0.01** | -0.22 | 0.90 | **-0.71** | **0.00** | 0.23 | 0.44 | **-0.54** | **0.01** | **-0.63** | **0.00** | -0.08 | 0.83 | **-0.46** | **0.04** |
| n.plate | bodyPC1 | 0.14 | 0.71 | -0.02 | 0.96 | 0.25 | 0.28 | -0.26 | 0.35 | -0.21 | 0.31 | -0.08 | 0.83 | -0.24 | 0.56 | 0.15 | 0.80 |
| n.plate | bodyPC2 | 0.05 | 0.91 | -0.15 | 0.91 | 0.30 | 0.21 | 0.13 | 0.62 | 0.09 | 0.64 | 0.38 | 0.09 | 0.18 | 0.64 | 0.26 | 0.39 |
| n.plate | DS1 | -0.02 | 0.96 | 0.19 | 0.90 | 0.10 | 0.65 | -0.05 | 0.81 | 0.34 | 0.10 | -0.04 | 0.94 | 0.22 | 0.57 | 0.16 | 0.80 |
| n.plate | DS2 | 0.09 | 0.79 | 0.04 | 0.96 | -0.04 | 0.87 | -0.10 | 0.73 | **0.49** | **0.01** | 0.23 | 0.39 | -0.19 | 0.64 | 0.02 | 0.99 |
| n.plate | BAP | 0.06 | 0.91 | -0.01 | 0.97 | **0.54** | **0.01** | 0.32 | 0.25 | -0.04 | 0.84 | 0.31 | 0.22 | 0.15 | 0.64 | 0.04 | 0.99 |
| n.plate | LP | 0.33 | 0.16 | -0.03 | 0.96 | 0.19 | 0.45 | 0.05 | 0.79 | 0.10 | 0.63 | -0.09 | 0.80 | 0.00 | 0.98 | -0.30 | 0.28 |
| n.plate | HP | -0.33 | 0.16 | 0.15 | 0.91 | 0.34 | 0.12 | -0.31 | 0.25 | -0.02 | 0.93 | -0.24 | 0.38 | 0.06 | 0.84 | -0.03 | 0.99 |
| n.plate | PS | -0.13 | 0.71 | 0.11 | 0.91 | 0.00 | 0.99 | -0.20 | 0.48 | 0.10 | 0.63 | 0.00 | 0.99 | -0.03 | 0.89 | 0.09 | 0.95 |
| bodyPC1 | bodyPC2 | 0.40 | 0.11 | -0.12 | 0.91 | 0.21 | 0.38 | 0.02 | 0.92 | -0.35 | 0.09 | -0.23 | 0.38 | 0.10 | 0.82 | 0.05 | 0.99 |
| bodyPC1 | DS1 | -0.03 | 0.96 | -0.02 | 0.96 | 0.11 | 0.63 | 0.13 | 0.62 | 0.28 | 0.19 | -0.15 | 0.63 | 0.30 | 0.54 | -0.31 | 0.26 |
| bodyPC1 | DS2 | 0.03 | 0.96 | 0.09 | 0.92 | -0.15 | 0.56 | 0.27 | 0.35 | 0.21 | 0.31 | -0.04 | 0.94 | 0.07 | 0.84 | -0.38 | 0.12 |
| bodyPC1 | BAP | 0.12 | 0.71 | 0.11 | 0.91 | 0.17 | 0.48 | 0.08 | 0.75 | 0.25 | 0.25 | -0.18 | 0.56 | 0.08 | 0.83 | -0.03 | 0.99 |
| bodyPC1 | LP | -0.07 | 0.85 | 0.12 | 0.91 | -0.14 | 0.56 | 0.22 | 0.44 | 0.11 | 0.63 | 0.09 | 0.80 | 0.08 | 0.83 | -0.17 | 0.77 |
| bodyPC1 | HP | 0.18 | 0.64 | 0.09 | 0.92 | 0.26 | 0.26 | 0.16 | 0.58 | 0.33 | 0.11 | 0.04 | 0.94 | -0.08 | 0.83 | -0.26 | 0.39 |
| bodyPC1 | PS | 0.12 | 0.71 | 0.06 | 0.95 | 0.12 | 0.62 | 0.22 | 0.44 | 0.27 | 0.20 | -0.14 | 0.67 | 0.15 | 0.64 | -0.21 | 0.57 |
| bodyPC2 | DS1 | 0.21 | 0.53 | 0.24 | 0.78 | 0.14 | 0.58 | -0.24 | 0.42 | -0.18 | 0.39 | 0.08 | 0.83 | -0.23 | 0.56 | 0.11 | 0.95 |
| bodyPC2 | DS2 | 0.10 | 0.78 | 0.06 | 0.95 | -0.09 | 0.71 | -0.30 | 0.25 | -0.18 | 0.39 | 0.04 | 0.94 | 0.22 | 0.57 | -0.01 | 0.99 |
| bodyPC2 | BAP | -0.01 | 0.96 | 0.09 | 0.92 | 0.29 | 0.22 | -0.06 | 0.79 | -0.22 | 0.31 | 0.43 | 0.06 | -0.04 | 0.84 | 0.09 | 0.95 |
| bodyPC2 | LP | 0.01 | 0.96 | -0.18 | 0.90 | 0.02 | 0.92 | -0.09 | 0.73 | -0.24 | 0.26 | 0.12 | 0.71 | 0.15 | 0.64 | -0.02 | 0.99 |
| bodyPC2 | HP | 0.14 | 0.71 | 0.25 | 0.78 | 0.27 | 0.23 | -0.33 | 0.25 | -0.19 | 0.38 | 0.32 | 0.21 | 0.17 | 0.64 | 0.08 | 0.97 |
| bodyPC2 | PS | 0.16 | 0.68 | 0.04 | 0.96 | 0.27 | 0.23 | -0.22 | 0.44 | -0.10 | 0.63 | 0.22 | 0.39 | 0.27 | 0.54 | 0.04 | 0.99 |
| DS1 | DS2 | **0.85** | **0.00** | **0.60** | **0.00** | 0.12 | 0.63 | **0.72** | **0.00** | **0.68** | **0.00** | **0.73** | **0.00** | 0.25 | 0.56 | **0.85** | **0.00** |
| DS1 | BAP | 0.23 | 0.47 | 0.41 | 0.19 | **0.45** | **0.03** | 0.31 | 0.25 | 0.38 | 0.06 | 0.28 | 0.28 | 0.26 | 0.54 | 0.09 | 0.95 |
| DS1 | LP | 0.39 | 0.12 | 0.06 | 0.95 | 0.21 | 0.37 | 0.15 | 0.59 | **0.52** | **0.01** | 0.01 | 0.99 | -0.05 | 0.84 | 0.03 | 0.99 |
| DS1 | HP | 0.34 | 0.16 | 0.13 | 0.91 | **0.44** | **0.03** | 0.18 | 0.53 | **0.52** | **0.01** | 0.11 | 0.78 | 0.05 | 0.84 | **0.53** | **0.01** |
| DS1 | PS | **0.62** | **0.00** | **0.61** | **0.00** | **0.45** | **0.03** | 0.19 | 0.50 | **0.59** | **0.00** | **0.72** | **0.00** | -0.11 | 0.78 | **0.50** | **0.03** |
| DS2 | BAP | 0.12 | 0.71 | 0.19 | 0.90 | **0.53** | **0.01** | 0.07 | 0.77 | **0.50** | **0.01** | 0.40 | 0.08 | 0.45 | 0.14 | 0.14 | 0.82 |
| DS2 | LP | 0.51 | **0.01** | -0.03 | 0.96 | 0.28 | 0.22 | 0.16 | 0.58 | **0.47** | **0.01** | 0.00 | 0.99 | 0.18 | 0.64 | -0.07 | 0.98 |
| DS2 | HP | 0.32 | 0.16 | 0.05 | 0.96 | 0.40 | 0.05 | 0.33 | 0.25 | **0.54** | **0.01** | 0.00 | 0.99 | 0.27 | 0.54 | 0.40 | 0.10 |
| DS2 | PS | **0.73** | **0.00** | 0.46 | 0.10 | **0.47** | **0.02** | 0.38 | 0.15 | **0.57** | **0.00** | **0.61** | **0.00** | 0.38 | 0.35 | **0.54** | **0.01** |
| BAP | LP | 0.16 | 0.68 | -0.14 | 0.91 | 0.27 | 0.23 | 0.13 | 0.62 | **0.53** | **0.01** | 0.39 | 0.08 | 0.28 | 0.54 | -0.01 | 0.99 |
| BAP | HP | 0.26 | 0.35 | 0.15 | 0.91 | **0.64** | **0.00** | -0.11 | 0.70 | **0.67** | **0.00** | -0.02 | 0.99 | 0.14 | 0.64 | 0.02 | 0.99 |
| BAP | PS | 0.07 | 0.85 | 0.34 | 0.46 | **0.56** | **0.00** | -0.07 | 0.78 | **0.53** | **0.01** | **0.55** | **0.01** | 0.27 | 0.54 | -0.38 | 0.12 |
| LP | HP | 0.13 | 0.71 | -0.03 | 0.96 | **0.57** | **0.00** | **0.52** | **0.02** | **0.54** | **0.01** | 0.39 | 0.08 | 0.05 | 0.84 | 0.07 | 0.98 |
| LP | PS | 0.37 | 0.12 | -0.07 | 0.95 | **0.43** | **0.04** | **0.58** | **0.00** | **0.53** | **0.01** | 0.29 | 0.27 | **0.56** | **0.03** | 0.07 | 0.98 |
| HP | PS | 0.53 | **0.01** | 0.26 | 0.78 | **0.80** | **0.00** | **0.83** | **0.00** | **0.65** | **0.00** | 0.15 | 0.63 | 0.24 | 0.56 | 0.35 | 0.17 |

Table S7 (continued)

|  |  | EISI |  | FADA |  | FEIT |  | FHAI |  | GEIR |  | GILL |  | GROG |  | HOST |  | IALA |  | MAGA |  |
| --- | --- | --- | --- | --- | --- | --- | --- | --- | --- | --- | --- | --- | --- | --- | --- | --- | --- | --- | --- | --- | --- |
| trait1 | trait2 | r | p-value | r | p-value | r | p-value | r | p-value | r | p-value | r | p-value | r | p-value | r | p-value | r | p-value | r | p-value |
| SL | n.plate | 0.35 | 0.07 | 0.04 | 0.86 | -0.18 | 0.54 | 0.00 | 0.99 | 0.08 | 0.83 | -0.20 | 0.59 | -0.01 | 0.99 | -0.05 | 0.88 | -0.07 | 0.99 | 0.30 | 0.16 |
| SL | bodyPC1 | -0.33 | 0.09 | 0.29 | 0.16 | -0.04 | 0.83 | 0.14 | 0.62 | 0.37 | 0.14 | **0.52** | **0.01** | -0.15 | 0.70 | 0.00 | 0.99 | -0.05 | 0.99 | 0.05 | 0.89 |
| SL | bodyPC2 | 0.10 | 0.63 | -0.23 | 0.25 | 0.17 | 0.57 | 0.08 | 0.79 | -0.12 | 0.79 | 0.18 | 0.61 | -0.38 | 0.09 | 0.25 | 0.41 | -0.30 | 0.27 | -0.22 | 0.32 |
| SL | DS1 | **-0.78** | **0.00** | **-0.57** | **0.00** | **-0.57** | **0.00** | **-0.80** | **0.00** | -0.44 | 0.06 | -0.13 | 0.75 | **-0.57** | **0.00** | -0.11 | 0.88 | **-0.66** | **0.00** | **-0.41** | **0.04** |
| SL | DS2 | **-0.65** | **0.00** | -0.32 | 0.14 | **-0.47** | **0.02** | **-0.72** | **0.00** | **-0.65** | **0.00** | -0.05 | 0.86 | **-0.73** | **0.00** | -0.10 | 0.88 | **-0.49** | **0.02** | **-0.41** | **0.04** |
| SL | BAP | **-0.44** | **0.02** | **-1.00** | **0.00** | **-0.56** | **0.00** | **-0.71** | **0.00** | -0.13 | 0.79 | -0.09 | 0.79 | 0.06 | 0.92 | -0.23 | 0.42 | 0.01 | 0.99 | -0.26 | 0.25 |
| SL | LP | **-0.59** | **0.00** | **-0.97** | **0.00** | **-0.49** | **0.01** | **-0.52** | **0.01** | 0.25 | 0.40 | -0.27 | 0.32 | -0.14 | 0.71 | -0.35 | 0.20 | -0.40 | 0.08 | -0.01 | 0.96 |
| SL | HP | **-0.67** | **0.00** | **-1.00** | **0.00** | **-0.51** | **0.01** | **-0.46** | **0.01** | -0.10 | 0.83 | **-0.53** | **0.01** | -0.33 | 0.14 | -0.22 | 0.45 | -0.31 | 0.27 | **-0.70** | **0.00** |
| SL | PS | **-0.83** | **0.00** | **-1.00** | **0.00** | **-0.63** | **0.00** | **-0.80** | **0.00** | **-0.67** | **0.00** | 0.03 | 0.89 | -0.51 | 0.02 | -0.06 | 0.88 | **-0.82** | **0.00** | **-0.46** | **0.02** |
| n.plate | bodyPC1 | -0.24 | 0.23 | -0.06 | 0.86 | 0.07 | 0.77 | 0.02 | 0.95 | 0.13 | 0.79 | -0.03 | 0.89 | 0.28 | 0.25 | -0.23 | 0.42 | 0.03 | 0.99 | 0.02 | 0.95 |
| n.plate | bodyPC2 | 0.30 | 0.13 | -0.27 | 0.20 | -0.05 | 0.80 | 0.04 | 0.86 | 0.34 | 0.17 | 0.14 | 0.73 | 0.09 | 0.81 | -0.25 | 0.41 | -0.06 | 0.99 | 0.18 | 0.42 |
| n.plate | DS1 | **-0.46** | **0.01** | -0.15 | 0.49 | 0.13 | 0.65 | 0.14 | 0.62 | -0.05 | 0.90 | 0.17 | 0.62 | 0.12 | 0.75 | 0.06 | 0.88 | 0.10 | 0.99 | 0.01 | 0.96 |
| n.plate | DS2 | **-0.57** | **0.00** | 0.03 | 0.89 | 0.09 | 0.73 | 0.11 | 0.68 | -0.26 | 0.38 | 0.09 | 0.79 | 0.04 | 0.95 | -0.08 | 0.88 | 0.06 | 0.99 | -0.03 | 0.95 |
| n.plate | BAP | **-0.44** | **0.02** | -0.02 | 0.90 | 0.12 | 0.68 | 0.51 | 0.01 | **0.75** | **0.00** | -0.10 | 0.79 | 0.40 | 0.09 | -0.13 | 0.86 | 0.12 | 0.95 | 0.00 | 0.98 |
| n.plate | LP | -0.06 | 0.75 | -0.05 | 0.86 | -0.13 | 0.65 | 0.04 | 0.86 | -0.07 | 0.83 | 0.05 | 0.86 | 0.01 | 0.99 | 0.01 | 0.98 | 0.05 | 0.99 | -0.35 | 0.11 |
| n.plate | HP | -0.26 | 0.20 | -0.04 | 0.86 | 0.06 | 0.80 | 0.08 | 0.77 | -0.38 | 0.13 | 0.12 | 0.77 | -0.02 | 0.99 | 0.05 | 0.88 | 0.00 | 0.99 | -0.46 | 0.02 |
| n.plate | PS | **-0.44** | **0.02** | -0.04 | 0.86 | 0.09 | 0.73 | 0.14 | 0.62 | -0.05 | 0.90 | 0.05 | 0.86 | 0.03 | 0.99 | -0.10 | 0.88 | 0.08 | 0.99 | 0.08 | 0.79 |
| bodyPC1 | bodyPC2 | -0.06 | 0.75 | -0.39 | 0.06 | 0.16 | 0.57 | 0.14 | 0.62 | 0.04 | 0.90 | 0.13 | 0.75 | 0.39 | 0.09 | 0.24 | 0.42 | -0.15 | 0.87 | 0.14 | 0.56 |
| bodyPC1 | DS1 | 0.30 | 0.13 | -0.04 | 0.86 | 0.26 | 0.27 | -0.12 | 0.66 | -0.35 | 0.16 | -0.03 | 0.89 | 0.19 | 0.55 | 0.34 | 0.20 | 0.23 | 0.50 | 0.14 | 0.56 |
| bodyPC1 | DS2 | 0.23 | 0.25 | -0.18 | 0.40 | 0.30 | 0.18 | -0.21 | 0.43 | -0.25 | 0.40 | 0.07 | 0.86 | 0.27 | 0.26 | 0.27 | 0.41 | 0.28 | 0.35 | 0.05 | 0.89 |
| bodyPC1 | BAP | 0.20 | 0.31 | -0.30 | 0.16 | 0.11 | 0.72 | -0.14 | 0.62 | 0.09 | 0.83 | -0.28 | 0.32 | 0.36 | 0.11 | 0.39 | 0.16 | 0.12 | 0.95 | 0.25 | 0.25 |
| bodyPC1 | LP | 0.15 | 0.47 | -0.29 | 0.16 | 0.11 | 0.72 | 0.10 | 0.73 | 0.16 | 0.72 | 0.03 | 0.89 | -0.12 | 0.75 | 0.02 | 0.97 | -0.02 | 0.99 | 0.24 | 0.26 |
| bodyPC1 | HP | 0.19 | 0.34 | -0.29 | 0.16 | 0.27 | 0.25 | 0.15 | 0.62 | 0.17 | 0.72 | -0.22 | 0.49 | 0.18 | 0.60 | 0.18 | 0.59 | 0.16 | 0.86 | 0.10 | 0.69 |
| bodyPC1 | PS | 0.24 | 0.23 | -0.29 | 0.16 | 0.16 | 0.57 | -0.23 | 0.36 | -0.17 | 0.72 | 0.19 | 0.59 | 0.36 | 0.11 | 0.46 | 0.08 | 0.00 | 0.99 | 0.24 | 0.26 |
| bodyPC2 | DS1 | -0.23 | 0.25 | 0.03 | 0.89 | 0.08 | 0.75 | -0.07 | 0.80 | 0.17 | 0.72 | -0.12 | 0.77 | 0.39 | 0.09 | 0.34 | 0.20 | 0.22 | 0.50 | **0.48** | **0.02** |
| bodyPC2 | DS2 | -0.31 | 0.12 | 0.26 | 0.22 | 0.00 | 0.98 | -0.11 | 0.68 | -0.07 | 0.83 | -0.18 | 0.61 | 0.36 | 0.11 | 0.28 | 0.39 | 0.00 | 0.99 | 0.38 | 0.08 |
| bodyPC2 | BAP | 0.09 | 0.67 | 0.23 | 0.25 | -0.19 | 0.54 | -0.18 | 0.54 | 0.39 | 0.13 | -0.11 | 0.79 | 0.38 | 0.09 | 0.27 | 0.41 | 0.35 | 0.17 | 0.37 | 0.08 |
| bodyPC2 | LP | -0.07 | 0.74 | 0.25 | 0.22 | -0.16 | 0.57 | -0.05 | 0.83 | -0.08 | 0.83 | -0.23 | 0.46 | -0.02 | 0.99 | -0.03 | 0.96 | 0.02 | 0.99 | 0.02 | 0.95 |
| bodyPC2 | HP | -0.01 | 0.97 | 0.23 | 0.25 | 0.08 | 0.75 | 0.07 | 0.80 | -0.12 | 0.79 | -0.09 | 0.79 | 0.28 | 0.25 | 0.05 | 0.88 | 0.04 | 0.99 | 0.11 | 0.66 |
| bodyPC2 | PS | -0.17 | 0.42 | 0.23 | 0.25 | -0.10 | 0.73 | -0.20 | 0.43 | 0.03 | 0.91 | -0.06 | 0.86 | 0.22 | 0.42 | 0.36 | 0.20 | 0.23 | 0.50 | 0.46 | 0.02 |
| DS1 | DS2 | **0.91** | **0.00** | 0.14 | 0.51 | **0.88** | **0.00** | **0.91** | **0.00** | **0.71** | **0.00** | **0.85** | **0.00** | **0.81** | **0.00** | **0.83** | **0.00** | **0.77** | **0.00** | **0.89** | **0.00** |
| DS1 | BAP | **0.47** | **0.01** | **0.56** | **0.00** | **0.57** | **0.00** | **0.60** | **0.00** | 0.15 | 0.75 | 0.39 | 0.10 | 0.01 | 0.99 | 0.38 | 0.17 | 0.14 | 0.88 | 0.29 | 0.18 |
| DS1 | LP | **0.63** | **0.00** | **0.53** | **0.00** | **0.52** | **0.01** | **0.50** | **0.01** | 0.14 | 0.79 | 0.30 | 0.26 | 0.08 | 0.81 | 0.07 | 0.88 | -0.06 | 0.99 | 0.20 | 0.39 |
| DS1 | HP | **0.67** | **0.00** | **0.57** | **0.00** | **0.62** | **0.00** | 0.37 | 0.07 | 0.03 | 0.91 | 0.44 | 0.05 | 0.50 | 0.02 | 0.17 | 0.61 | 0.44 | 0.04 | **0.60** | **0.00** |
| DS1 | PS | **0.85** | **0.00** | **0.57** | **0.00** | **0.74** | **0.00** | **0.80** | **0.00** | **0.58** | **0.00** | **0.62** | **0.00** | **0.68** | **0.00** | **0.75** | **0.00** | **0.55** | **0.00** | **0.79** | **0.00** |
| DS2 | BAP | **0.47** | **0.01** | 0.31 | 0.14 | **0.57** | **0.00** | **0.51** | **0.01** | 0.00 | 1.00 | **0.48** | **0.03** | 0.00 | 0.99 | 0.25 | 0.41 | 0.12 | 0.95 | 0.28 | 0.20 |
| DS2 | LP | **0.57** | **0.00** | 0.33 | 0.14 | **0.42** | **0.04** | **0.46** | **0.01** | 0.00 | 1.00 | 0.31 | 0.26 | 0.13 | 0.75 | 0.06 | 0.88 | 0.03 | 0.99 | 0.32 | 0.15 |
| DS2 | HP | **0.59** | **0.00** | 0.32 | 0.14 | **0.71** | **0.00** | 0.23 | 0.36 | 0.33 | 0.18 | 0.31 | 0.26 | 0.32 | 0.17 | 0.07 | 0.88 | 0.49 | 0.02 | **0.64** | **0.00** |
| DS2 | PS | **0.76** | **0.00** | 0.32 | 0.14 | **0.82** | **0.00** | **0.80** | **0.00** | **0.80** | **0.00** | **0.61** | **0.00** | **0.76** | **0.00** | **0.63** | **0.00** | 0.48 | 0.02 | **0.70** | **0.00** |
| BAP | LP | **0.54** | **0.00** | **0.97** | **0.00** | **0.41** | **0.04** | **0.52** | **0.01** | -0.26 | 0.38 | 0.39 | 0.10 | -0.10 | 0.80 | 0.06 | 0.88 | -0.06 | 0.99 | 0.32 | 0.15 |
| BAP | HP | 0.36 | 0.07 | **1.00** | **0.00** | **0.74** | **0.00** | **0.51** | **0.01** | -0.10 | 0.83 | **0.52** | **0.01** | -0.12 | 0.75 | 0.20 | 0.51 | 0.08 | 0.99 | 0.31 | 0.15 |
| BAP | PS | **0.48** | **0.01** | **1.00** | **0.00** | **0.63** | **0.00** | **0.62** | **0.00** | 0.14 | 0.79 | 0.42 | 0.07 | -0.08 | 0.81 | 0.44 | 0.08 | -0.01 | 0.99 | **0.47** | **0.02** |
| LP | HP | **0.59** | **0.00** | **0.97** | **0.00** | 0.34 | 0.11 | 0.38 | 0.05 | 0.10 | 0.83 | **0.65** | **0.00** | 0.11 | 0.77 | 0.02 | 0.97 | -0.05 | 0.99 | 0.34 | 0.11 |
| LP | PS | **0.51** | **0.01** | **0.97** | **0.00** | **0.43** | **0.03** | **0.43** | **0.02** | -0.07 | 0.83 | **0.57** | **0.01** | -0.14 | 0.71 | 0.09 | 0.88 | **0.57** | **0.00** | 0.17 | 0.48 |
| HP | PS | **0.68** | **0.00** | **1.00** | **0.00** | **0.78** | **0.00** | 0.38 | 0.06 | 0.36 | 0.14 | **0.47** | **0.04** | 0.42 | 0.07 | 0.08 | 0.88 | 0.23 | 0.50 | **0.69** | **0.00** |

Table S7 (continued)

|  |  | MAIG |  | MORA |  | NAGE |  | REIV |  | SAND |  | SCAD |  | STRU |  | TORM |  | TROS |  | all |  |
| --- | --- | --- | --- | --- | --- | --- | --- | --- | --- | --- | --- | --- | --- | --- | --- | --- | --- | --- | --- | --- | --- |
| trait1 | trait2 | r | p-value | r | p-value | r | p-value | r | p-value | r | p-value | r | p-value | r | p-value | r | p-value | r | p-value | r | p-value |
| SL | n.plate | -0.07 | 0.84 | -0.03 | 0.90 | 0.52 | 0.02 | -0.18 | 0.45 | 0.31 | 0.13 | -0.30 | 0.18 | -0.14 | 0.53 | 0.48 | 0.09 | -0.30 | 0.35 | **0.31** | **0.00** |
| SL | bodyPC1 | 0.19 | 0.56 | -0.10 | 0.77 | 0.00 | 1.00 | -0.58 | 0.00 | **-0.51** | **0.01** | -0.15 | 0.53 | -0.17 | 0.46 | 0.09 | 0.80 | -0.10 | 0.91 | **0.08** | **0.03** |
| SL | bodyPC2 | 0.13 | 0.71 | -0.29 | 0.31 | 0.27 | 0.37 | 0.42 | 0.06 | 0.19 | 0.37 | 0.25 | 0.27 | -0.11 | 0.60 | -0.34 | 0.38 | **-0.54** | **0.01** | 0.01 | 0.83 |
| SL | DS1 | -0.22 | 0.47 | -0.28 | 0.31 | -0.29 | 0.30 | -0.39 | 0.09 | **-0.89** | **0.00** | -0.50 | 0.01 | **-0.50** | **0.01** | **-0.73** | **0.00** | -0.36 | 0.24 | **0.09** | **0.01** |
| SL | DS2 | **-0.73** | **0.00** | -0.36 | 0.25 | -0.24 | 0.39 | -0.33 | 0.15 | **-0.55** | **0.00** | -0.39 | 0.07 | -0.35 | 0.08 | -0.05 | 0.88 | -0.22 | 0.59 | **0.16** | **0.00** |
| SL | BAP | **-0.97** | **0.00** | **-0.97** | **0.00** | -0.29 | 0.30 | -0.24 | 0.31 | -0.39 | 0.07 | -0.98 | 0.00 | **-0.86** | **0.00** | **-0.68** | **0.00** | 0.03 | 0.97 | **0.17** | **0.00** |
| SL | LP | -0.37 | 0.08 | -0.14 | 0.65 | -0.30 | 0.30 | -0.53 | 0.01 | -0.26 | 0.22 | -0.18 | 0.45 | **-0.58** | **0.00** | -0.19 | 0.56 | 0.01 | 0.97 | **0.10** | **0.00** |
| SL | HP | **-0.60** | **0.00** | **-1.00** | **0.00** | **-0.65** | **0.00** | -0.38 | 0.09 | **-0.62** | **0.00** | **-0.99** | **0.00** | **-0.66** | **0.00** | 0.05 | 0.88 | -0.05 | 0.97 | **0.18** | **0.00** |
| SL | PS | **-1.00** | **0.00** | **-1.00** | **0.00** | 0.06 | 0.90 | -0.09 | 0.72 | **-0.87** | **0.00** | **-1.00** | **0.00** | **-0.61** | **0.00** | -0.44 | 0.13 | **-0.54** | **0.01** | **0.18** | **0.00** |
| n.plate | bodyPC1 | -0.07 | **0.84** | **0.02** | 0.90 | 0.17 | 0.60 | 0.11 | 0.66 | -0.15 | 0.47 | -0.37 | 0.08 | 0.16 | 0.46 | 0.12 | 0.76 | -0.21 | 0.59 | **0.43** | **0.00** |
| n.plate | bodyPC2 | -0.01 | 0.97 | 0.17 | 0.59 | 0.34 | 0.30 | -0.17 | 0.46 | 0.14 | 0.49 | -0.28 | 0.20 | 0.36 | 0.07 | -0.33 | 0.38 | 0.07 | 0.97 | 0.04 | 0.25 |
| n.plate | DS1 | 0.22 | 0.47 | -0.05 | 0.87 | 0.21 | 0.48 | 0.09 | 0.71 | -0.33 | 0.11 | 0.07 | 0.74 | 0.08 | 0.71 | -0.31 | 0.40 | 0.02 | 0.97 | **0.61** | **0.00** |
| n.plate | DS2 | -0.01 | 0.97 | -0.06 | 0.85 | 0.06 | 0.90 | 0.12 | 0.63 | -0.30 | 0.16 | 0.13 | 0.55 | -0.15 | 0.52 | 0.29 | 0.40 | -0.03 | 0.97 | **0.64** | **0.00** |
| n.plate | BAP | 0.18 | 0.57 | 0.27 | 0.33 | -0.13 | 0.71 | -0.11 | 0.66 | -0.12 | 0.57 | 0.45 | 0.03 | **0.59** | **0.00** | 0.19 | 0.56 | -0.02 | 0.97 | **0.81** | **0.00** |
| n.plate | LP | -0.02 | 0.96 | -0.18 | 0.58 | -0.14 | 0.70 | 0.00 | 0.98 | 0.02 | 0.92 | -0.15 | 0.53 | 0.02 | 0.89 | -0.18 | 0.57 | -0.13 | 0.82 | **0.47** | **0.00** |
| n.plate | HP | 0.29 | 0.25 | 0.03 | 0.90 | -0.21 | 0.48 | -0.06 | 0.80 | -0.37 | 0.08 | 0.29 | 0.18 | 0.10 | 0.63 | -0.21 | 0.56 | -0.22 | 0.59 | **0.66** | **0.00** |
| n.plate | PS | 0.07 | 0.84 | 0.03 | 0.90 | 0.29 | 0.30 | 0.08 | 0.73 | -0.34 | 0.10 | 0.30 | 0.18 | 0.24 | 0.28 | -0.49 | 0.08 | 0.15 | 0.79 | **0.64** | **0.00** |
| bodyPC1 | bodyPC2 | -0.12 | 0.71 | -0.24 | 0.40 | 0.17 | 0.59 | -0.43 | 0.05 | -0.10 | 0.61 | -0.33 | 0.12 | -0.22 | 0.31 | 0.06 | 0.87 | 0.35 | 0.24 | 0.00 | 0.93 |
| bodyPC1 | DS1 | 0.03 | 0.96 | 0.31 | 0.31 | -0.01 | 0.99 | 0.33 | 0.14 | 0.43 | 0.03 | 0.12 | 0.57 | 0.25 | 0.26 | -0.31 | 0.40 | 0.26 | 0.49 | **0.38** | **0.00** |
| bodyPC1 | DS2 | 0.01 | 0.97 | 0.07 | 0.85 | -0.11 | 0.75 | 0.30 | 0.18 | 0.13 | 0.53 | 0.03 | 0.89 | 0.23 | 0.29 | 0.07 | 0.84 | 0.22 | 0.59 | **0.41** | **0.00** |
| bodyPC1 | BAP | -0.17 | 0.64 | 0.10 | 0.77 | 0.24 | 0.39 | 0.20 | 0.40 | 0.25 | 0.22 | 0.10 | 0.65 | 0.20 | 0.37 | 0.20 | 0.56 | 0.31 | 0.35 | **0.46** | **0.00** |
| bodyPC1 | LP | 0.12 | 0.71 | -0.03 | 0.90 | 0.14 | 0.69 | 0.58 | 0.00 | 0.01 | 0.94 | 0.07 | 0.75 | 0.04 | 0.83 | 0.12 | 0.76 | -0.01 | 0.97 | **0.33** | **0.00** |
| bodyPC1 | HP | -0.10 | 0.79 | 0.10 | 0.77 | 0.07 | 0.90 | 0.20 | 0.40 | 0.39 | 0.07 | 0.12 | 0.57 | 0.13 | 0.57 | 0.10 | 0.80 | 0.28 | 0.45 | **0.44** | **0.00** |
| bodyPC1 | PS | -0.19 | 0.56 | 0.10 | 0.77 | -0.08 | 0.90 | 0.34 | 0.14 | 0.34 | 0.10 | 0.15 | 0.53 | 0.23 | 0.28 | 0.01 | 0.97 | 0.05 | 0.97 | **0.36** | **0.00** |
| bodyPC2 | DS1 | -0.05 | 0.91 | -0.20 | 0.53 | 0.46 | 0.05 | 0.16 | 0.48 | -0.18 | 0.38 | -0.04 | 0.87 | -0.17 | 0.46 | 0.26 | 0.53 | 0.39 | 0.17 | 0.03 | 0.46 |
| bodyPC2 | DS2 | -0.12 | 0.71 | 0.27 | 0.33 | -0.10 | 0.79 | 0.08 | 0.73 | -0.21 | 0.33 | -0.19 | 0.42 | -0.29 | 0.17 | 0.21 | 0.56 | 0.16 | 0.72 | -0.02 | 0.61 |
| bodyPC2 | BAP | -0.08 | 0.84 | 0.32 | 0.31 | 0.05 | 0.92 | 0.05 | 0.82 | -0.02 | 0.92 | -0.30 | 0.18 | 0.24 | 0.28 | 0.30 | 0.40 | 0.18 | 0.65 | 0.06 | 0.08 |
| bodyPC2 | LP | 0.13 | 0.71 | 0.30 | 0.31 | -0.02 | 0.98 | -0.18 | 0.45 | 0.18 | 0.38 | -0.01 | 0.97 | -0.09 | 0.67 | -0.13 | 0.75 | 0.08 | 0.97 | 0.04 | 0.31 |
| bodyPC2 | HP | -0.13 | 0.71 | 0.29 | 0.31 | 0.01 | 0.99 | 0.22 | 0.36 | -0.21 | 0.33 | -0.26 | 0.23 | 0.12 | 0.59 | -0.09 | 0.80 | 0.05 | 0.97 | -0.04 | 0.30 |
| bodyPC2 | PS | -0.13 | 0.71 | 0.29 | 0.31 | 0.31 | 0.30 | 0.01 | 0.98 | -0.28 | 0.20 | -0.25 | 0.27 | 0.05 | 0.81 | 0.21 | 0.56 | 0.32 | 0.34 | 0.00 | 0.93 |
| DS1 | DS2 | -0.03 | 0.96 | -0.09 | 0.77 | 0.25 | 0.39 | 0.83 | 0.00 | **0.62** | **0.00** | -0.49 | 0.01 | **0.56** | **0.00** | -0.19 | 0.56 | **0.87** | **0.00** | **0.73** | **0.00** |
| DS1 | BAP | 0.22 | 0.47 | 0.26 | 0.34 | 0.04 | 0.92 | 0.47 | 0.04 | 0.34 | 0.10 | 0.48 | 0.02 | **0.44** | **0.02** | 0.40 | 0.20 | -0.03 | 0.97 | **0.71** | **0.00** |
| DS1 | LP | 0.08 | 0.84 | -0.18 | 0.58 | -0.14 | 0.69 | 0.26 | 0.26 | 0.20 | 0.33 | 0.21 | 0.36 | **0.60** | **0.00** | 0.17 | 0.61 | 0.08 | 0.97 | **0.58** | **0.00** |
| DS1 | HP | 0.39 | 0.07 | 0.28 | 0.31 | 0.51 | 0.02 | 0.45 | 0.04 | **0.69** | **0.00** | 0.54 | 0.01 | **0.48** | **0.01** | -0.20 | 0.56 | -0.21 | 0.59 | **0.74** | **0.00** |
| DS1 | PS | 0.22 | 0.47 | 0.28 | 0.31 | 0.61 | 0.00 | 0.39 | 0.09 | **0.88** | **0.00** | 0.50 | 0.01 | **0.70** | **0.00** | 0.09 | 0.80 | **0.73** | **0.00** | **0.78** | **0.00** |
| DS2 | BAP | **0.69** | **0.00** | 0.33 | 0.31 | -0.04 | 0.92 | 0.30 | 0.18 | 0.20 | 0.33 | 0.39 | 0.07 | 0.30 | 0.15 | 0.35 | 0.38 | -0.11 | 0.89 | **0.74** | **0.00** |
| DS2 | LP | 0.07 | 0.84 | 0.23 | 0.42 | -0.04 | 0.92 | 0.20 | 0.40 | 0.09 | 0.65 | -0.12 | 0.57 | **0.64** | **0.00** | 0.04 | 0.90 | 0.00 | 0.99 | **0.54** | **0.00** |
| DS2 | HP | 0.43 | 0.04 | 0.36 | 0.25 | 0.12 | 0.74 | 0.31 | 0.17 | **0.44** | **0.03** | 0.35 | 0.11 | **0.50** | **0.01** | 0.03 | 0.91 | -0.12 | 0.89 | **0.78** | **0.00** |
| DS2 | PS | **0.73** | **0.00** | 0.36 | 0.25 | 0.18 | 0.59 | 0.37 | 0.10 | **0.52** | **0.01** | 0.39 | 0.07 | **0.64** | **0.00** | -0.20 | 0.56 | **0.61** | **0.00** | **0.80** | **0.00** |
| BAP | LP | 0.42 | 0.05 | 0.10 | 0.77 | 0.22 | 0.47 | 0.21 | 0.40 | **0.35** | **0.10** | 0.14 | 0.54 | **0.46** | **0.01** | 0.13 | 0.75 | 0.27 | 0.45 | **0.54** | **0.00** |
| BAP | HP | **0.68** | **0.00** | **0.97** | **0.00** | 0.29 | 0.30 | 0.26 | 0.26 | **0.45** | **0.03** | **0.97** | **0.00** | **0.59** | **0.00** | -0.23 | 0.56 | 0.03 | 0.97 | **0.79** | **0.00** |
| BAP | PS | **0.97** | **0.00** | **0.97** | **0.00** | -0.06 | 0.90 | 0.31 | 0.17 | 0.26 | 0.22 | **0.98** | **0.00** | **0.62** | **0.00** | 0.20 | 0.56 | -0.09 | 0.94 | **0.73** | **0.00** |
| LP | HP | 0.50 | 0.01 | 0.14 | 0.65 | 0.25 | 0.39 | 0.46 | 0.04 | 0.26 | 0.22 | 0.19 | 0.42 | **0.63** | **0.00** | 0.21 | 0.56 | -0.16 | 0.72 | **0.67** | **0.00** |
| LP | PS | 0.37 | 0.08 | 0.14 | 0.65 | -0.30 | 0.30 | 0.35 | 0.13 | 0.27 | 0.22 | 0.18 | 0.45 | **0.79** | **0.00** | -0.08 | 0.80 | 0.03 | 0.97 | **0.67** | **0.00** |
| HP | PS | **0.60** | **0.00** | **1.00** | **0.00** | 0.29 | 0.30 | 0.14 | 0.58 | **0.57** | **0.00** | **0.99** | **0.00** | **0.73** | **0.00** | 0.02 | 0.94 | 0.19 | 0.63 | **0.90** | **0.00** |

Table S8. Loadings of abiotic environmental variables on the axes of environmental variation and the percent variance explained by each axis. The highest loadings on each of the first 10 PCs are in bold.

|  | PC1 | PC2 | PC3 | PC4 | PC5 | PC6 | PC7 | PC8 | PC9 | PC10 | PC11 | PC12 | PC13 | PC14 | PC15 | PC16 | PC17 | PC18 | PC19 | PC20 | PC21 | PC22 | PC23 | PC24 | PC25 |
| --- | --- | --- | --- | --- | --- | --- | --- | --- | --- | --- | --- | --- | --- | --- | --- | --- | --- | --- | --- | --- | --- | --- | --- | --- | --- |
| mean_depth | -0.104 | **-0.425** | 0.101 | -0.177 | 0.000 | -0.256 | 0.090 | -0.138 | 0.232 | -0.226 | 0.027 | -0.018 | 0.128 | -0.237 | 0.184 | -0.126 | 0.201 | -0.165 | 0.274 | -0.225 | -0.480 | -0.104 | 0.102 | 0.023 | 0.009 |
| max_depth | -0.126 | **-0.425** | 0.165 | -0.167 | -0.096 | -0.191 | 0.029 | -0.068 | 0.053 | -0.069 | 0.223 | -0.121 | 0.264 | 0.042 | 0.018 | 0.002 | -0.199 | -0.012 | 0.036 | 0.457 | 0.404 | 0.276 | -0.149 | 0.208 | 0.003 |
| area | -0.110 | **-0.263** | **0.221** | 0.105 | **-0.335** | -0.141 | -0.233 | 0.074 | **-0.421** | 0.287 | 0.396 | -0.021 | -0.052 | 0.217 | -0.013 | 0.162 | -0.095 | 0.043 | -0.154 | -0.207 | -0.247 | -0.049 | 0.027 | -0.177 | -0.004 |
| pH | **0.310** | -0.043 | 0.015 | -0.092 | -0.068 | -0.059 | 0.070 | 0.030 | -0.117 | -0.053 | 0.032 | 0.055 | 0.143 | -0.113 | 0.119 | 0.112 | 0.232 | 0.168 | -0.263 | -0.439 | 0.247 | 0.318 | 0.384 | 0.381 | 0.017 |
| conductivity | **0.314** | -0.017 | 0.109 | -0.019 | -0.042 | -0.045 | 0.043 | 0.013 | -0.041 | -0.070 | 0.088 | -0.031 | -0.013 | -0.093 | 0.119 | 0.015 | -0.097 | 0.067 | -0.235 | 0.087 | -0.005 | -0.717 | -0.247 | 0.438 | 0.004 |
| salinity | **0.305** | -0.045 | 0.022 | -0.022 | -0.040 | 0.069 | 0.020 | -0.056 | -0.041 | -0.077 | 0.047 | 0.100 | -0.236 | 0.128 | 0.170 | 0.459 | 0.145 | 0.447 | 0.513 | 0.241 | -0.126 | 0.071 | -0.007 | 0.019 | -0.002 |
| mud | 0.080 | 0.102 | **0.380** | 0.107 | **0.515** | -0.125 | 0.124 | 0.208 | -0.013 | 0.254 | 0.129 | -0.116 | 0.120 | -0.179 | 0.118 | 0.164 | 0.049 | -0.081 | 0.053 | -0.018 | 0.022 | 0.043 | -0.062 | -0.097 | -0.527 |
| softpeat | -0.116 | 0.224 | **0.259** | **-0.405** | -0.171 | -0.033 | 0.011 | 0.273 | 0.140 | -0.016 | 0.166 | 0.259 | -0.227 | 0.271 | -0.342 | -0.164 | 0.166 | -0.126 | 0.171 | 0.015 | 0.016 | -0.053 | 0.134 | 0.261 | -0.233 |
| hardpeat | -0.121 | 0.320 | 0.160 | -0.163 | -0.220 | -0.180 | -0.139 | -0.256 | -0.190 | -0.344 | -0.364 | -0.093 | 0.399 | 0.203 | 0.040 | 0.186 | 0.020 | 0.080 | -0.060 | -0.030 | -0.056 | -0.042 | -0.098 | -0.092 | -0.315 |
| Sand | 0.184 | -0.069 | **-0.275** | 0.158 | **-0.349** | -0.076 | 0.289 | -0.327 | 0.148 | 0.242 | -0.021 | 0.136 | -0.099 | -0.015 | -0.033 | -0.156 | -0.270 | 0.022 | 0.014 | -0.045 | -0.069 | 0.095 | -0.016 | 0.059 | -0.561 |
| Gravel | -0.045 | **-0.205** | **-0.423** | -0.155 | **0.358** | 0.172 | -0.076 | -0.167 | 0.092 | -0.092 | 0.198 | -0.179 | 0.048 | 0.332 | -0.262 | 0.361 | 0.057 | -0.134 | -0.202 | 0.000 | -0.139 | -0.083 | 0.125 | 0.120 | -0.209 |
| Rock | -0.160 | **-0.275** | -0.148 | 0.084 | -0.083 | 0.303 | **-0.383** | 0.273 | -0.151 | -0.311 | -0.050 | -0.030 | -0.189 | -0.204 | 0.188 | -0.211 | 0.144 | 0.129 | -0.013 | 0.044 | 0.143 | -0.041 | 0.009 | -0.010 | -0.456 |
| Cu | -0.074 | -0.025 | 0.023 | **-0.582** | -0.159 | 0.299 | 0.210 | 0.188 | 0.225 | 0.072 | 0.009 | -0.047 | -0.011 | -0.246 | 0.105 | 0.234 | -0.266 | 0.208 | -0.225 | -0.106 | -0.029 | -0.016 | -0.070 | -0.308 | -0.014 |
| Zn | **-0.248** | -0.042 | -0.014 | -0.026 | 0.057 | 0.105 | **0.503** | 0.055 | -0.174 | 0.102 | -0.091 | -0.051 | -0.035 | 0.427 | 0.478 | -0.216 | 0.227 | 0.081 | -0.191 | 0.191 | -0.093 | -0.032 | 0.117 | 0.036 | -0.004 |
| Cd | -0.197 | 0.172 | -0.002 | 0.010 | -0.093 | 0.128 | **0.407** | -0.270 | **-0.421** | -0.282 | 0.310 | -0.130 | -0.223 | -0.329 | -0.106 | 0.145 | 0.069 | -0.250 | 0.123 | -0.031 | 0.154 | -0.037 | 0.000 | -0.019 | -0.001 |
| Mn | -0.111 | -0.045 | -0.051 | **0.461** | -0.226 | 0.004 | 0.276 | **0.419** | 0.244 | -0.283 | 0.058 | 0.306 | 0.275 | 0.037 | -0.065 | 0.342 | 0.017 | -0.104 | -0.073 | 0.041 | 0.003 | -0.082 | 0.057 | -0.069 | -0.006 |
| Pb | -0.173 | -0.124 | **0.322** | 0.218 | 0.127 | -0.205 | 0.112 | -0.084 | 0.245 | -0.290 | -0.161 | -0.235 | -0.504 | 0.137 | -0.152 | 0.046 | -0.198 | 0.282 | -0.198 | -0.190 | 0.067 | 0.041 | 0.039 | 0.012 | 0.003 |
| Na | **0.279** | 0.000 | 0.101 | -0.013 | 0.050 | 0.166 | 0.065 | 0.252 | -0.264 | -0.236 | -0.137 | -0.165 | 0.038 | 0.038 | -0.038 | -0.103 | -0.546 | -0.192 | 0.084 | 0.146 | -0.339 | 0.160 | 0.329 | 0.127 | 0.006 |
| Ca | **0.289** | -0.111 | 0.010 | -0.063 | -0.118 | -0.251 | 0.068 | 0.007 | -0.031 | 0.028 | -0.098 | -0.164 | -0.043 | -0.146 | -0.282 | -0.061 | 0.297 | 0.040 | -0.201 | 0.405 | 0.062 | -0.188 | 0.418 | -0.404 | -0.036 |
| Mg | **0.297** | -0.079 | -0.038 | -0.034 | -0.118 | -0.024 | 0.132 | 0.219 | -0.064 | -0.105 | -0.086 | -0.195 | -0.121 | 0.043 | -0.173 | -0.031 | 0.317 | -0.154 | -0.194 | -0.005 | -0.234 | 0.351 | -0.613 | 0.010 | 0.009 |
| K | **0.291** | -0.083 | 0.056 | -0.047 | -0.149 | 0.033 | -0.077 | -0.005 | 0.158 | -0.036 | -0.077 | -0.155 | -0.184 | 0.306 | 0.349 | 0.124 | -0.026 | -0.540 | 0.142 | -0.192 | 0.371 | -0.113 | 0.032 | -0.229 | -0.002 |
| Sulphate | 0.098 | -0.178 | **0.308** | 0.159 | -0.094 | **0.556** | 0.137 | -0.125 | 0.070 | 0.091 | -0.026 | -0.258 | 0.324 | 0.133 | -0.313 | -0.182 | 0.140 | 0.166 | 0.214 | -0.183 | 0.087 | -0.128 | -0.014 | -0.032 | 0.002 |
| DOC | 0.030 | **0.398** | -0.178 | 0.097 | -0.172 | -0.109 | -0.079 | 0.122 | 0.292 | -0.109 | 0.479 | -0.540 | 0.093 | 0.039 | 0.176 | -0.173 | 0.015 | 0.174 | 0.061 | 0.006 | -0.083 | 0.050 | 0.073 | -0.006 | 0.004 |
| ChlA | 0.160 | 0.110 | **0.349** | 0.084 | 0.026 | 0.308 | -0.173 | **-0.370** | 0.211 | -0.191 | 0.281 | 0.313 | -0.044 | -0.014 | 0.168 | -0.079 | 0.071 | -0.113 | -0.361 | 0.220 | -0.182 | 0.184 | 0.037 | -0.108 | -0.006 |
| orangeratio | **0.244** | -0.063 | -0.131 | -0.120 | 0.273 | -0.156 | 0.144 | 0.034 | -0.166 | **-0.341** | 0.281 | 0.296 | 0.119 | 0.221 | -0.056 | -0.325 | -0.142 | 0.219 | 0.057 | -0.209 | 0.153 | -0.069 | -0.145 | -0.370 | -0.009 |
|  |  |  |  |  |  |  |  |  |  |  |  |  |  |  |  |  |  |  |  |  |  |  |  |  |  |
| St deviation | 3.085 | 1.818 | 1.569 | 1.384 | 1.208 | 1.104 | 0.996 | 0.921 | 0.839 | 0.742 | 0.694 | 0.596 | 0.567 | 0.496 | 0.435 | 0.357 | 0.324 | 0.284 | 0.228 | 0.159 | 0.133 | 0.103 | 0.089 | 0.031 | 0.003 |
| variance explained | 0.381 | 0.132 | 0.098 | 0.077 | 0.058 | 0.049 | 0.040 | 0.034 | 0.028 | 0.022 | 0.019 | 0.014 | 0.013 | 0.010 | 0.008 | 0.005 | 0.004 | 0.003 | 0.002 | 0.001 | 0.001 | 0.000 | 0.000 | 0.000 | 0.000 |
| Cumulative % variance  explained | 0.381 | 0.513 | 0.612 | 0.688 | 0.747 | 0.795 | 0.835 | 0.869 | 0.897 | 0.919 | 0.938 | 0.953 | 0.965 | 0.975 | 0.983 | 0.988 | 0.992 | 0.995 | 0.998 | 0.999 | 0.999 | 1.000 | 1.000 | 1.000 | 1.000 |

Table S9. Pearson correlation coefficients (r), P-values and FDR corrected P-values of correlations among abiotic environmental variables. Significant values are in bold.

|  | mean_  depth | max_  depth | Area | pH | Cond. | Salin. | mud | Soft  peat | Hard  peat | Sand | Gravel | Rock | Cu | Zn | Cd | Mn | Pb | Na | Ca | Mg | K. | Sulph. | DOC | ChlA | Orange  ratio |
| --- | --- | --- | --- | --- | --- | --- | --- | --- | --- | --- | --- | --- | --- | --- | --- | --- | --- | --- | --- | --- | --- | --- | --- | --- | --- |
| mean_depth | | **0.00** | 0.15 | 0.52 | 0.42 | 0.38 | 0.60 | 0.95 | 0.60 | 0.63 | 0.40 | 0.22 | 0.39 | 0.34 | 0.90 | 0.86 | 0.07 | 0.22 | 0.92 | 0.52 | 0.63 | 0.92 | 0.01 | 0.36 | 0.78 |
| max_depth | **0.91** |  | **0.00** | 0.38 | 0.33 | 0.26 | 0.60 | 0.82 | 0.71 | 0.48 | 0.54 | 0.16 | 0.33 | 0.25 | 0.96 | 0.78 | 0.08 | 0.22 | 0.70 | 0.38 | 0.53 | 0.64 | **0.02** | 0.36 | 0.54 |
| area | 0.39 | **0.64** |  | 0.39 | 0.44 | 0.38 | 0.64 | 0.74 | 1.00 | 0.58 | 0.48 | 0.12 | 0.81 | 0.52 | 0.71 | 0.52 | 0.24 | 0.33 | 0.61 | 0.41 | 0.54 | 0.60 | 0.23 | 0.60 | 0.16 |
| pH | -0.19 | -0.25 | -0.24 |  | **0.00** | **0.00** | 0.51 | 0.28 | 0.25 | **0.02** | 0.59 | 0.07 | 0.70 | **0.00** | **0.02** | 0.22 | 0.02 | **0.00** | **0.00** | **0.00** | **0.00** | 0.35 | 0.96 | 0.12 | **0.00** |
| conductivity | -0.23 | -0.27 | -0.22 | **0.96** |  | **0.00** | 0.25 | 0.33 | 0.25 | **0.04** | 0.34 | **0.04** | 0.54 | **0.00** | **0.02** | 0.24 | 0.11 | **0.00** | **0.00** | **0.00** | **0.00** | 0.22 | 0.86 | **0.02** | **0.00** |
| salinity | -0.25 | -0.31 | -0.25 | **0.91** | **0.92** |  | 0.56 | 0.24 | 0.17 | **0.02** | 0.70 | 0.14 | 0.59 | **0.00** | **0.03** | 0.24 | 0.07 | **0.00** | **0.00** | **0.00** | **0.00** | 0.23 | 0.97 | **0.03** | **0.00** |
| mud | -0.15 | -0.15 | -0.13 | 0.20 | 0.32 | 0.17 |  | 0.90 | 0.66 | 0.19 | 0.23 | 0.05 | 0.39 | 0.66 | 0.54 | 0.60 | 0.44 | 0.23 | 0.62 | 0.70 | 0.71 | 0.52 | 0.86 | 0.19 | 0.46 |
| softpeat | -0.02 | 0.07 | 0.09 | -0.30 | -0.28 | -0.33 | 0.04 |  | **0.03** | **0.04** | 0.22 | 0.61 | 0.01 | 0.42 | 0.33 | 0.70 | 0.63 | 0.39 | 0.25 | 0.27 | 0.34 | 0.50 | 0.60 | 0.94 | 0.25 |
| hardpeat | -0.15 | -0.10 | 0.00 | -0.31 | -0.31 | -0.38 | -0.12 | **0.52** |  | 0.19 | 0.16 | 0.53 | 0.68 | 0.59 | 0.11 | 0.72 | 0.63 | 0.32 | 0.23 | 0.12 | 0.23 | 0.32 | 0.33 | 0.96 | 0.15 |
| Sand | -0.13 | -0.21 | -0.17 | **0.54** | **0.49** | **0.54** | -0.36 | **-0.51** | -0.37 |  | 0.97 | 0.25 | 0.45 | 0.22 | 0.40 | 0.87 | 0.12 | 0.33 | **0.01** | **0.02** | **0.02** | 0.67 | 0.63 | 0.83 | 0.20 |
| Gravel | 0.24 | 0.18 | -0.21 | -0.16 | -0.27 | -0.11 | -0.33 | -0.35 | -0.38 | 0.01 |  | 0.20 | 0.63 | 0.61 | 0.90 | 0.59 | 0.55 | 0.48 | 0.59 | 0.69 | 0.60 | 0.54 | 0.59 | 0.19 | 0.44 |
| Rock | 0.35 | 0.39 | 0.41 | -0.46 | -0.51 | -0.40 | -0.49 | -0.14 | -0.19 | -0.32 | 0.36 |  | 0.71 | 0.32 | 0.72 | 0.25 | 0.53 | 0.25 | 0.08 | 0.23 | 0.22 | 0.96 | 0.27 | 0.21 | 0.19 |
| Cu | 0.25 | 0.28 | -0.07 | -0.11 | -0.18 | -0.16 | -0.24 | 0.58 | 0.12 | -0.22 | 0.13 | 0.10 |  | 0.29 | 0.58 | 0.42 | 0.60 | 0.67 | 0.54 | 0.69 | 0.70 | 0.96 | 0.67 | 0.59 | 0.53 |
| Zn | 0.27 | 0.32 | 0.19 | **-0.69** | **-0.73** | **-0.68** | -0.13 | 0.23 | 0.16 | -0.35 | 0.15 | 0.28 | 0.30 |  | 0.01 | 0.25 | 0.13 | **0.01** | **0.00** | **0.01** | **0.00** | 0.70 | 0.46 | **0.04** | 0.06 |
| Cd | -0.04 | 0.01 | 0.10 | **-0.54** | **-0.55** | **-0.53** | -0.18 | 0.27 | 0.42 | -0.24 | -0.04 | 0.10 | 0.16 | 0.62 |  | 0.52 | 0.38 | 0.09 | **0.01** | **0.02** | **0.00** | 0.59 | 0.65 | 0.53 | 0.12 |
| Mn | 0.05 | 0.08 | 0.19 | -0.34 | -0.33 | -0.33 | -0.15 | -0.10 | -0.10 | 0.05 | -0.16 | 0.31 | -0.23 | 0.32 | 0.19 |  | 0.24 | 0.36 | 0.25 | 0.50 | 0.25 | 0.90 | 0.68 | 0.40 | 0.25 |
| Pb | 0.47 | 0.45 | 0.32 | -0.55 | -0.42 | -0.46 | 0.22 | 0.13 | 0.14 | -0.42 | -0.17 | 0.19 | -0.15 | 0.41 | 0.25 | 0.33 |  | 0.12 | 0.16 | 0.06 | 0.15 | 0.81 | 0.33 | 0.90 | 0.08 |
| Na | -0.34 | -0.34 | -0.27 | **0.83** | **0.87** | **0.80** | 0.33 | -0.25 | -0.28 | 0.28 | -0.21 | -0.31 | -0.12 | **-0.57** | -0.44 | -0.26 | -0.41 |  | **0.00** | **0.00** | **0.00** | 0.12 | 0.94 | 0.11 | **0.00** |
| Ca | -0.03 | -0.10 | -0.14 | **0.91** | **0.89** | **0.82** | 0.14 | -0.31 | -0.34 | **0.60** | -0.16 | -0.45 | -0.18 | **-0.69** | **-0.59** | -0.32 | -0.38 | **0.72** |  | **0.00** | **0.00** | 0.49 | 0.92 | 0.39 | **0.00** |
| Mg | -0.19 | -0.25 | -0.24 | **0.92** | **0.90** | **0.86** | 0.11 | -0.30 | -0.42 | **0.56** | -0.11 | -0.33 | -0.11 | **-0.62** | **-0.54** | -0.20 | -0.47 | **0.85** | **0.92** |  | **0.00** | 0.31 | 0.91 | 0.38 | **0.00** |
| K | -0.14 | -0.19 | -0.18 | **0.86** | **0.89** | **0.89** | 0.10 | -0.27 | -0.34 | **0.53** | -0.15 | -0.35 | -0.10 | **-0.68** | **-0.65** | -0.31 | -0.39 | **0.77** | **0.83** | **0.87** |  | 0.19 | 0.94 | 0.05 | **0.01** |
| Sulphate | 0.03 | 0.13 | 0.15 | 0.27 | 0.34 | 0.34 | 0.19 | -0.20 | -0.28 | 0.12 | -0.18 | 0.01 | 0.01 | -0.11 | -0.16 | 0.04 | 0.07 | 0.41 | 0.20 | 0.29 | 0.37 |  | 0.24 | **0.02** | 0.94 |
| DOC | -0.57 | **-0.56** | -0.34 | 0.01 | 0.05 | -0.01 | -0.05 | 0.15 | 0.28 | 0.14 | -0.16 | -0.30 | -0.12 | -0.21 | 0.13 | 0.12 | -0.28 | -0.02 | -0.03 | 0.04 | 0.02 | -0.33 |  | 0.90 | 0.94 |
| ChlA | -0.26 | -0.26 | -0.15 | 0.41 | **0.55** | **0.51** | 0.37 | 0.02 | 0.01 | 0.06 | -0.36 | -0.35 | -0.16 | **-0.50** | -0.18 | -0.24 | -0.04 | 0.43 | 0.25 | 0.25 | 0.49 | **0.54** | 0.04 |  | 0.48 |
| Orange.ratio | -0.08 | -0.18 | -0.38 | **0.77** | **0.72** | **0.70** | 0.21 | -0.31 | -0.39 | 0.36 | 0.22 | -0.37 | -0.18 | -0.47 | -0.41 | -0.32 | -0.45 | **0.67** | **0.67** | **0.70** | **0.57** | -0.02 | -0.03 | 0.21 |  |

Table S10. Summary statistics for the 18 populations from North Uist with RAD sequencing data as outputted by the program POPULATIONS. Statistics shown for analyses including a) only nucleotide positions that are polymorphic in at least one population (Variant positions) and b) for analyses including all nucleotide positions.

| a) Variant positions | |  |  |  |  |  |  |  |  |  |  |  |  |  |  |  |  |  |  |  |  |  |  |  |  |
| --- | --- | --- | --- | --- | --- | --- | --- | --- | --- | --- | --- | --- | --- | --- | --- | --- | --- | --- | --- | --- | --- | --- | --- | --- | --- |
| pop _ID | Private | Num | Var | Std | P | Var | Std | Obs | Var | Std | Ob | Var | Std | Exp | Var | Std | Exp | Var | Std | π | Var | Std | F_IS_ | Var | Std |
|  |  | ind |  | Err |  |  | Err | Het |  | Err | sHom |  | Err | Het |  | Err | Hom |  | Err |  |  | Err |  |  | Err |
| whole genome | |  |  |  |  |  |  |  |  |  |  |  |  |  |  |  |  |  |  |  |  |  |  |  |  |
| FADA | 0 | 14.662 | 0.440 | 0.007 | 0.862 | 0.024 | 0.002 | 0.178 | 0.041 | 0.002 | 0.822 | 0.041 | 0.002 | 0.191 | 0.035 | 0.002 | 0.810 | 0.035 | 0.002 | 0.197 | 0.037 | 0.002 | 0.076 | 16.672 | 0.007 |
| IALA | 6 | 17.758 | 0.320 | 0.006 | 0.895 | 0.021 | 0.002 | 0.151 | 0.039 | 0.002 | 0.849 | 0.039 | 0.002 | 0.146 | 0.031 | 0.002 | 0.854 | 0.031 | 0.002 | 0.151 | 0.033 | 0.002 | 0.003 | 22.596 | 0.006 |
| BUAI | 31 | 16.803 | 0.266 | 0.005 | 0.962 | 0.010 | 0.001 | 0.059 | 0.023 | 0.002 | 0.942 | 0.023 | 0.002 | 0.053 | 0.015 | 0.001 | 0.947 | 0.015 | 0.001 | 0.055 | 0.016 | 0.001 | -0.007 | 36.128 | 0.005 |
| AONG | 1 | 17.718 | 0.391 | 0.007 | 0.864 | 0.026 | 0.002 | 0.187 | 0.046 | 0.002 | 0.813 | 0.046 | 0.002 | 0.184 | 0.038 | 0.002 | 0.817 | 0.038 | 0.002 | 0.189 | 0.040 | 0.002 | 0.008 | 20.626 | 0.007 |
| GEIR | 0 | 16.438 | 0.697 | 0.009 | 0.837 | 0.024 | 0.002 | 0.222 | 0.040 | 0.002 | 0.778 | 0.040 | 0.002 | 0.225 | 0.033 | 0.002 | 0.775 | 0.033 | 0.002 | 0.232 | 0.036 | 0.002 | 0.034 | 11.876 | 0.009 |
| EISI | 1 | 16.550 | 0.574 | 0.008 | 0.906 | 0.020 | 0.002 | 0.134 | 0.038 | 0.002 | 0.866 | 0.038 | 0.002 | 0.131 | 0.031 | 0.002 | 0.869 | 0.031 | 0.002 | 0.135 | 0.033 | 0.002 | 0.008 | 26.459 | 0.008 |
| HOST | 0 | 18.675 | 0.407 | 0.007 | 0.842 | 0.024 | 0.002 | 0.220 | 0.043 | 0.002 | 0.780 | 0.043 | 0.002 | 0.217 | 0.034 | 0.002 | 0.783 | 0.034 | 0.002 | 0.223 | 0.036 | 0.002 | 0.013 | 12.461 | 0.007 |
| REIV | 1 | 18.690 | 0.357 | 0.006 | 0.842 | 0.026 | 0.002 | 0.221 | 0.047 | 0.002 | 0.780 | 0.047 | 0.002 | 0.214 | 0.037 | 0.002 | 0.786 | 0.037 | 0.002 | 0.220 | 0.039 | 0.002 | 0.004 | 15.254 | 0.006 |
| AROI | 1 | 18.655 | 0.436 | 0.007 | 0.905 | 0.021 | 0.002 | 0.136 | 0.039 | 0.002 | 0.865 | 0.039 | 0.002 | 0.130 | 0.031 | 0.002 | 0.870 | 0.031 | 0.002 | 0.134 | 0.033 | 0.002 | -0.002 | 24.081 | 0.007 |
| GILL | 0 | 16.501 | 0.643 | 0.008 | 0.866 | 0.025 | 0.002 | 0.180 | 0.043 | 0.002 | 0.820 | 0.043 | 0.002 | 0.182 | 0.037 | 0.002 | 0.818 | 0.037 | 0.002 | 0.188 | 0.040 | 0.002 | 0.022 | 21.226 | 0.008 |
| TROS | 0 | 19.465 | 0.678 | 0.009 | 0.847 | 0.025 | 0.002 | 0.213 | 0.045 | 0.002 | 0.787 | 0.045 | 0.002 | 0.209 | 0.035 | 0.002 | 0.791 | 0.035 | 0.002 | 0.215 | 0.037 | 0.002 | 0.008 | 14.135 | 0.009 |
| STRU | 0 | 15.568 | 0.510 | 0.008 | 0.879 | 0.023 | 0.002 | 0.175 | 0.046 | 0.002 | 0.825 | 0.046 | 0.002 | 0.166 | 0.034 | 0.002 | 0.834 | 0.034 | 0.002 | 0.172 | 0.037 | 0.002 | 0.000 | 19.878 | 0.008 |
| TORM | 1 | 18.630 | 0.472 | 0.007 | 0.856 | 0.026 | 0.002 | 0.200 | 0.046 | 0.002 | 0.800 | 0.046 | 0.002 | 0.196 | 0.037 | 0.002 | 0.804 | 0.037 | 0.002 | 0.202 | 0.039 | 0.002 | 0.008 | 17.774 | 0.007 |
| BHAR | 0 | 17.658 | 0.427 | 0.007 | 0.847 | 0.025 | 0.002 | 0.216 | 0.044 | 0.002 | 0.784 | 0.044 | 0.002 | 0.211 | 0.035 | 0.002 | 0.789 | 0.035 | 0.002 | 0.217 | 0.037 | 0.002 | 0.005 | 14.412 | 0.007 |
| MORA | 0 | 20.656 | 0.469 | 0.007 | 0.841 | 0.024 | 0.002 | 0.224 | 0.043 | 0.002 | 0.776 | 0.043 | 0.002 | 0.219 | 0.034 | 0.002 | 0.781 | 0.034 | 0.002 | 0.224 | 0.036 | 0.002 | 0.005 | 12.507 | 0.007 |
| SCAD | 0 | 16.673 | 0.394 | 0.007 | 0.837 | 0.025 | 0.002 | 0.228 | 0.043 | 0.002 | 0.772 | 0.043 | 0.002 | 0.224 | 0.034 | 0.002 | 0.776 | 0.034 | 0.002 | 0.231 | 0.036 | 0.002 | 0.011 | 12.237 | 0.007 |
| DAIM | 7 | 19.616 | 0.542 | 0.008 | 0.917 | 0.021 | 0.002 | 0.115 | 0.040 | 0.002 | 0.885 | 0.040 | 0.002 | 0.110 | 0.032 | 0.002 | 0.890 | 0.032 | 0.002 | 0.113 | 0.033 | 0.002 | -0.003 | 31.506 | 0.008 |
| CHRU | 25 | 16.840 | 0.225 | 0.005 | 0.965 | 0.011 | 0.001 | 0.051 | 0.025 | 0.002 | 0.950 | 0.025 | 0.002 | 0.045 | 0.016 | 0.001 | 0.955 | 0.016 | 0.001 | 0.047 | 0.017 | 0.001 | -0.007 | 42.270 | 0.005 |

| b) All positions (variant and fixed) | | |  |  |  |  |  |  |  |  |  |  |  |  |  |  |  |  |  |  |  |  |  |  |  |  |  |  |  |
| --- | --- | --- | --- | --- | --- | --- | --- | --- | --- | --- | --- | --- | --- | --- | --- | --- | --- | --- | --- | --- | --- | --- | --- | --- | --- | --- | --- | --- | --- |
| pop _ID | Private | Sites | Variant | Poly. | %Poly | N | Var | Std | P | Var | Std | Obs | Var | Std | Obs | Var | Std | Exp | Var | Std | Exp | Var | Std | π | Var | Std | F_IS_ | Var | Std |
|  |  |  | sites | Sites | loci | ind |  | Err |  |  | Err | Het |  | Err | Hom |  | Err | Het |  | Err | Hom |  | Err |  |  | Err |  |  | Err |
| Whole genome | |  |  |  |  |  |  |  |  |  |  |  |  |  |  |  |  |  |  |  |  |  |  |  |  |  |  |  |  |
| FADA | 0 | 1122281 | 9135 | 6108 | 0.544 | 14.886 | 0.154 | 0.000 | 0.999 | 0.000 | 0.000 | 0.002 | 0.001 | 0.000 | 0.999 | 0.001 | 0.000 | 0.002 | 0.001 | 0.000 | 0.998 | 0.001 | 0.000 | 0.002 | 0.001 | 0.000 | 0.001 | 48.743 | 0.000 |
| IALA | 6 | 1122281 | 9135 | 4933 | 0.440 | 17.967 | 0.049 | 0.000 | 0.999 | 0.000 | 0.000 | 0.001 | 0.001 | 0.000 | 0.999 | 0.001 | 0.000 | 0.001 | 0.000 | 0.000 | 0.999 | 0.000 | 0.000 | 0.001 | 0.001 | 0.000 | 0.000 | 48.785 | 0.000 |
| BUAI | 31 | 1122281 | 9135 | 2390 | 0.213 | 16.933 | 0.091 | 0.000 | 1.000 | 0.000 | 0.000 | 0.001 | 0.000 | 0.000 | 1.000 | 0.000 | 0.000 | 0.000 | 0.000 | 0.000 | 1.000 | 0.000 | 0.000 | 0.000 | 0.000 | 0.000 | 0.000 | 48.895 | 0.000 |
| AONG | 1 | 1122281 | 9135 | 5305 | 0.473 | 17.956 | 0.062 | 0.000 | 0.999 | 0.000 | 0.000 | 0.002 | 0.001 | 0.000 | 0.999 | 0.001 | 0.000 | 0.002 | 0.001 | 0.000 | 0.999 | 0.001 | 0.000 | 0.002 | 0.001 | 0.000 | 0.000 | 48.770 | 0.000 |
| GEIR | 0 | 1122281 | 9135 | 6952 | 0.620 | 16.879 | 0.165 | 0.000 | 0.999 | 0.000 | 0.000 | 0.002 | 0.001 | 0.000 | 0.998 | 0.001 | 0.000 | 0.002 | 0.001 | 0.000 | 0.998 | 0.001 | 0.000 | 0.002 | 0.001 | 0.000 | 0.000 | 48.701 | 0.000 |
| EISI | 1 | 1122281 | 9135 | 4221 | 0.376 | 16.858 | 0.200 | 0.000 | 0.999 | 0.000 | 0.000 | 0.001 | 0.001 | 0.000 | 0.999 | 0.001 | 0.000 | 0.001 | 0.000 | 0.000 | 0.999 | 0.000 | 0.000 | 0.001 | 0.000 | 0.000 | 0.000 | 48.817 | 0.000 |
| HOST | 0 | 1122281 | 9135 | 6829 | 0.609 | 18.964 | 0.050 | 0.000 | 0.999 | 0.000 | 0.000 | 0.002 | 0.001 | 0.000 | 0.998 | 0.001 | 0.000 | 0.002 | 0.001 | 0.000 | 0.998 | 0.001 | 0.000 | 0.002 | 0.001 | 0.000 | 0.000 | 48.704 | 0.000 |
| REIV | 1 | 1122281 | 9135 | 6302 | 0.562 | 18.906 | 0.103 | 0.000 | 0.999 | 0.000 | 0.000 | 0.002 | 0.001 | 0.000 | 0.998 | 0.001 | 0.000 | 0.002 | 0.001 | 0.000 | 0.998 | 0.001 | 0.000 | 0.002 | 0.001 | 0.000 | 0.000 | 48.726 | 0.000 |
| AROI | 1 | 1122281 | 9135 | 4649 | 0.414 | 18.947 | 0.074 | 0.000 | 0.999 | 0.000 | 0.000 | 0.001 | 0.001 | 0.000 | 0.999 | 0.001 | 0.000 | 0.001 | 0.000 | 0.000 | 0.999 | 0.000 | 0.000 | 0.001 | 0.000 | 0.000 | 0.000 | 48.797 | 0.000 |
| GILL | 0 | 1122281 | 9135 | 5210 | 0.464 | 16.870 | 0.189 | 0.000 | 0.999 | 0.000 | 0.000 | 0.002 | 0.001 | 0.000 | 0.999 | 0.001 | 0.000 | 0.002 | 0.001 | 0.000 | 0.999 | 0.001 | 0.000 | 0.002 | 0.001 | 0.000 | 0.000 | 48.775 | 0.000 |
| TROS | 0 | 1122281 | 9135 | 6514 | 0.580 | 19.895 | 0.129 | 0.000 | 0.999 | 0.000 | 0.000 | 0.002 | 0.001 | 0.000 | 0.998 | 0.001 | 0.000 | 0.002 | 0.001 | 0.000 | 0.998 | 0.001 | 0.000 | 0.002 | 0.001 | 0.000 | 0.000 | 48.717 | 0.000 |
| STRU | 0 | 1122281 | 9135 | 5437 | 0.485 | 15.966 | 0.049 | 0.000 | 0.999 | 0.000 | 0.000 | 0.001 | 0.001 | 0.000 | 0.999 | 0.001 | 0.000 | 0.001 | 0.001 | 0.000 | 0.999 | 0.001 | 0.000 | 0.001 | 0.001 | 0.000 | 0.000 | 48.763 | 0.000 |
| TORM | 1 | 1122281 | 9135 | 5837 | 0.520 | 18.968 | 0.048 | 0.000 | 0.999 | 0.000 | 0.000 | 0.002 | 0.001 | 0.000 | 0.998 | 0.001 | 0.000 | 0.002 | 0.001 | 0.000 | 0.998 | 0.001 | 0.000 | 0.002 | 0.001 | 0.000 | 0.000 | 48.747 | 0.000 |
| BHAR | 0 | 1122281 | 9135 | 6460 | 0.576 | 17.928 | 0.094 | 0.000 | 0.999 | 0.000 | 0.000 | 0.002 | 0.001 | 0.000 | 0.998 | 0.001 | 0.000 | 0.002 | 0.001 | 0.000 | 0.998 | 0.001 | 0.000 | 0.002 | 0.001 | 0.000 | 0.000 | 48.719 | 0.000 |
| MORA | 0 | 1122281 | 9135 | 6814 | 0.607 | 20.966 | 0.050 | 0.000 | 0.999 | 0.000 | 0.000 | 0.002 | 0.001 | 0.000 | 0.998 | 0.001 | 0.000 | 0.002 | 0.001 | 0.000 | 0.998 | 0.001 | 0.000 | 0.002 | 0.001 | 0.000 | 0.000 | 48.703 | 0.000 |
| SCAD | 0 | 1122281 | 9135 | 6870 | 0.612 | 16.965 | 0.049 | 0.000 | 0.999 | 0.000 | 0.000 | 0.002 | 0.001 | 0.000 | 0.998 | 0.001 | 0.000 | 0.002 | 0.001 | 0.000 | 0.998 | 0.001 | 0.000 | 0.002 | 0.001 | 0.000 | 0.000 | 48.702 | 0.000 |
| DAIM | 7 | 1122281 | 9135 | 3261 | 0.291 | 19.932 | 0.106 | 0.000 | 0.999 | 0.000 | 0.000 | 0.001 | 0.000 | 0.000 | 0.999 | 0.000 | 0.000 | 0.001 | 0.000 | 0.000 | 0.999 | 0.000 | 0.000 | 0.001 | 0.000 | 0.000 | 0.000 | 48.858 | 0.000 |
| CHRU | 25 | 1122281 | 9135 | 1244 | 0.111 | 16.941 | 0.079 | 0.000 | 1.000 | 0.000 | 0.000 | 0.000 | 0.000 | 0.000 | 1.000 | 0.000 | 0.000 | 0.000 | 0.000 | 0.000 | 1.000 | 0.000 | 0.000 | 0.000 | 0.000 | 0.000 | 0.000 | 48.945 | 0.000 |

Table S11. F_ST_ values based on whole genome among 18 freshwater populations of three-spined stickleback from North Uist. Highest and lowest values in bold.

|  | FADA | IALA | BUAI | AONG | GEIR | EISI | HOST | REIV | AROI | GILL | TROS | STRU | TORM | BHAR | MORA | SCAD | DAIM |
| --- | --- | --- | --- | --- | --- | --- | --- | --- | --- | --- | --- | --- | --- | --- | --- | --- | --- |
| a) whole genome | |  |  |  |  |  |  |  |  |  |  |  |  |  |  |  |  |
| IALA | 0.188 |  |  |  |  |  |  |  |  |  |  |  |  |  |  |  |  |
| BUAI | 0.289 | 0.340 |  |  |  |  |  |  |  |  |  |  |  |  |  |  |  |
| AONG | 0.161 | 0.212 | 0.316 |  |  |  |  |  |  |  |  |  |  |  |  |  |  |
| GEIR | 0.114 | 0.157 | 0.251 | 0.103 |  |  |  |  |  |  |  |  |  |  |  |  |  |
| EISI | 0.206 | 0.258 | 0.387 | 0.209 | 0.137 |  |  |  |  |  |  |  |  |  |  |  |  |
| HOST | 0.122 | 0.166 | 0.253 | 0.136 | 0.096 | 0.179 |  |  |  |  |  |  |  |  |  |  |  |
| REIV | 0.137 | 0.179 | 0.279 | 0.152 | 0.109 | 0.195 | 0.100 |  |  |  |  |  |  |  |  |  |  |
| AROI | 0.211 | 0.255 | 0.368 | 0.224 | 0.174 | 0.279 | 0.180 | 0.201 |  |  |  |  |  |  |  |  |  |
| GILL | 0.162 | 0.212 | 0.324 | 0.178 | 0.128 | 0.227 | 0.134 | 0.155 | 0.196 |  |  |  |  |  |  |  |  |
| TROS | 0.136 | 0.177 | 0.268 | 0.148 | 0.107 | 0.190 | 0.112 | 0.131 | 0.147 | **0.024** |  |  |  |  |  |  |  |
| STRU | 0.170 | 0.220 | 0.322 | 0.190 | 0.142 | 0.237 | 0.149 | 0.165 | 0.246 | 0.189 | 0.160 |  |  |  |  |  |  |
| TORM | 0.142 | 0.198 | 0.290 | 0.164 | 0.118 | 0.208 | 0.129 | 0.143 | 0.217 | 0.169 | 0.142 | 0.170 |  |  |  |  |  |
| BHAR | 0.124 | 0.173 | 0.262 | 0.147 | 0.100 | 0.188 | 0.112 | 0.124 | 0.193 | 0.146 | 0.123 | 0.142 | 0.122 |  |  |  |  |
| MORA | 0.125 | 0.177 | 0.253 | 0.145 | 0.101 | 0.188 | 0.114 | 0.127 | 0.189 | 0.145 | 0.124 | 0.105 | 0.129 | 0.095 |  |  |  |
| SCAD | 0.123 | 0.176 | 0.257 | 0.142 | 0.097 | 0.185 | 0.109 | 0.123 | 0.189 | 0.142 | 0.120 | 0.105 | 0.127 | 0.092 | **0.023** |  |  |
| DAIM | 0.246 | 0.307 | **0.447** | 0.266 | 0.205 | 0.329 | 0.217 | 0.232 | 0.328 | 0.274 | 0.230 | 0.234 | 0.249 | 0.215 | 0.164 | 0.160 |  |
| CHRU | 0.281 | 0.374 | **0.575** | 0.338 | 0.265 | 0.424 | 0.266 | 0.290 | 0.400 | 0.343 | 0.283 | 0.331 | 0.311 | 0.280 | 0.251 | 0.255 | **0.473** |

1. Summary of initial model with all environmental variables

Call: rda(formula = Resp ~ envPC1 + envPC2 + envPC3 + envPC4 + envPC5 + envPC6 + envPC7 + envPC8 +envPC9 + envPC10, scale = TRUE)

Partitioning of correlations:

|  | Inertia | Proportion (R^2^) | Adjusted R^2^ |
| --- | --- | --- | --- |
| Total | 10 | 1 |  |
| Constrained | 5.851 | 0.5851 | 0.326 |
| Unconstrained | 4.149 | 0.4149 |  |

Eigenvalues and their contribution to the correlations

Importance of components:

|  | RDA1 | RDA2 | RDA3 | RDA4 | RDA5 | RDA6 | RDA7 | RDA8 | RDA9 | RDA10 |
| --- | --- | --- | --- | --- | --- | --- | --- | --- | --- | --- |
| Eigenvalue | 4.394 | 0.784 | 0.328 | 0.198 | 0.064 | 0.048 | 0.020 | 0.010 | 0.004 | 0.000 |
| Proportion Explained | 0.439 | 0.078 | 0.033 | 0.020 | 0.006 | 0.005 | 0.002 | 0.001 | 0.000 | 0.000 |
| Cumulative proportion | 0.439 | 0.518 | 0.551 | 0.570 | 0.577 | 0.582 | 0.584 | 0.585 | 0.585 | 0.585 |

Result of permutation test of RDA results (1000 permutations).

|  | Df | Var | F | Pr(>F) |
| --- | --- | --- | --- | --- |
| Model | 10 | 5.851 | 2.2564 | 0.015 |
| Residual | 16 | 4.149 |  |  |

Backward selection of variables- last step:

| variable | Df | AIC | F | Pr(>F) |  |
| --- | --- | --- | --- | --- | --- |
| envPC2 | 1 | 58.466 | 7.5113 | 0.01 | ** |
| envPC1 | 1 | 59.591 | 8.8525 | 0.005 | ** |

2. Summary of results of most parsimonious model for abiotic variables

Call: rda(formula = Resp~envPC1+envPC2, scale=TRUE)

Partitioning of correlations:

|  | Inertia | Proportion (R^2^) | Adjusted R^2^ |
| --- | --- | --- | --- |
| Total | 10 | 1 |  |
| Constrained | 4.464 | 0.446 | 0.356 |
| Unconstrained | 5.536 | 0.554 |  |

Eigenvalues and their contribution to the correlations

Importance of components:

|  | | RDA1 | RDA2 | |
| --- | --- | --- | --- | --- |
| Eigenvalue | 3.6675 | | 0.38662 |  |
| Proportion explained | 0.3668 | | 0.03866 |  |
| Cumulative proportion | 0.3668 | | 0.40541 |  |

Permutation test for rda under reduced model:

|  | Df | Var | F | Pr(>F) |  |
| --- | --- | --- | --- | --- | --- |
| Model | 2 | 4.0541 | 8.1819 | 0.001 | *** |
| Residual | 24 | 5.9459 |  |  |  |
| *Tests for axes* | |  |  |  |  |
| RDA1 | 1 | 3.6675 | 14.8033 | 0.001 | *** |
| RDA2 | 1 | 0.3866 | 1.5605 | 0.196 |  |
| Residual | 24 | 5.9459 |  |  |  |
| *Tests for variables* | |  |  |  |  |
| envPC1 | 1 | 2.1932 | 8.8525 | 0.002 | ** |
| envPC2 | 1 | 1.8609 | 7.5113 | 0.001 | *** |
| Residual | 24 | 5.9459 |  |  |  |

3. Summary of Initial model with all biotic environmental variables

Call: rda(formula = Resp ~ %Pung + trout + pungitius + gyro + schisto, scale = TRUE)

Partitioning of correlations:

|  | Inertia | Proportion | Adjusted R^2^ |
| --- | --- | --- | --- |
| Total | 10 | 1 |  |
| Constrained | 4.456 | 0.4456 | 0.314 |
| Unconstrained | 5.544 | 0.5544 |  |

Eigenvalues and their contribution to the correlations

Importance of components:

|  | RDA1 | RDA2 | RDA3 | RDA4 | RDA5 |
| --- | --- | --- | --- | --- | --- |
| Eigenvalue | 3.3041 | 0.69456 | 0.3529 | 0.07272 | 0.03166 |
| Proportion explained | 0.3304 | 0.06946 | 0.03529 | 0.00727 | 0.00317 |
| Cumulative proportion | 0.3304 | 0.39987 | 0.43516 | 0.44243 | 0.4456 |

Permutation test for rda under reduced model:

|  | Df | Variance | F | Pr(>F) |  |
| --- | --- | --- | --- | --- | --- |
| Model | 5 | 4.456 | 3.3757 | 0.005 | ** |
| Residual | 21 | 5.544 |  |  |  |
| *Tests for axes* | |  |  |  |  |
| RDA1 | 1 | 3.3041 | 12.5156 | 0.001 | *** |
| RDA2 | 1 | 0.6946 | 2.6309 | 0.053 | . |
| RDA3 | 1 | 0.3529 | 1.3367 | 0.242 |  |
| RDA4 | 1 | 0.0727 | 0.2755 | 0.86 |  |
| RDA5 | 1 | 0.0317 | 0.1199 | 0.981 |  |
| Residual | 21 | 5.544 |  |  |  |
| *Tests for variables* | |  |  |  |  |
| X.Pung | 1 | 1.7933 | 6.7929 | 0.003 | ** |
| trout | 1 | 1.3628 | 5.1621 | 0.009 | ** |
| pungitius | 1 | 0.3126 | 1.1842 | 0.286 |  |
| gyro | 1 | 0.7191 | 2.7237 | 0.073 | . |
| schisto | 1 | 0.2682 | 1.0158 | 0.31 |  |
| Residual | 21 | 5.544 |  |  |  |

Backward selection of variables- last step:

|  | Df | AIC | F | Pr(>F) |  |
| --- | --- | --- | --- | --- | --- |
| Trout_presence | 1 | 59.815 | 4.779 | 0.025 | * |
| %Pung | 1 | 62.172 | 7.4042 | 0.015 | * |

4. Summary of results of most parsimonious model for biotic variables

Call: rda(formula = Resp~%Pung + trout, scale=TRUE)

Partitioning of correlations:

|  | Inertia | Proportion | Adjusted R^2^ |
| --- | --- | --- | --- |
| Total | 10 | 1 |  |
| Constrained | 3.156 | 0.3156 | 0.25858 |
| Unconstrained | 6.844 | 0.6844 |  |

Eigenvalues and their contribution to the correlations

Importance of components:

|  | RDA1 | RDA2 |
| --- | --- | --- |
| Eigenvalue | 2.5819 | 0.5742 |
| Proportion explained | 0.2582 | 0.05742 |
| Cumulative proportion | 0.2582 | 0.31561 |

Permutation test for rda under reduced model:

|  | Df | Variance | F | Pr(>F) |  |
| --- | --- | --- | --- | --- | --- |
| Model | 2 | 3.1561 | 5.5339 | 0.007 | ** |
| Residual | 24 | 6.8439 |  |  |  |
| *Tests for axes* | |  |  |  |  |
| RDA1 | 1 | 2.5819 | 9.0542 | 0.003 | ** |
| RDA2 | 1 | 0.5742 | 2.0136 | 0.111 |  |
| Residual | 24 | 6.8439 |  |  |  |
| *Tests for variables* | |  |  |  |  |
| %Pung | 1 | 1.7933 | 6.2888 | 0.007 | ** |
| Trout_presence | 1 | 1.3628 | 4.779 | 0.021 | * |
| Residual | 24 | 6.8439 |  |  |  |

5. Summary of initial model with all abiotic environmental variables –reduced dataset

Call: rda(formula = Resp ~ envPC1 + envPC2 + envPC3 + envPC4 + envPC5 + envPC6 + envPC7 + envPC8 +envPC9 + envPC10, scale = TRUE)

Partitioning of correlations:

|  | Inertia | Proportion | Adjusted R^2^ |
| --- | --- | --- | --- |
| Total | 10 | 1 |  |
| Constrained | 6.032 | 0.603 | 0.272 |
| Unconstrained | 3.968 | 0.397 |  |

Eigenvalues and their contribution to the correlations

Importance of components:

|  | RDA1 | RDA2 | RDA3 | RDA4 | RDA5 | RDA6 | RDA7 | RDA8 | RDA9 | RDA10 |
| --- | --- | --- | --- | --- | --- | --- | --- | --- | --- | --- |
| Eigenvalue | 4.541 | 0.825 | 0.300 | 0.205 | 0.066 | 0.046 | 0.023 | 0.017 | 0.010 | 0.000 |
| Proportion explained | 0.454 | 0.083 | 0.030 | 0.020 | 0.007 | 0.005 | 0.002 | 0.002 | 0.001 | 0.000 |
| Cumulative proportion | 0.454 | 0.537 | 0.567 | 0.587 | 0.594 | 0.598 | 0.601 | 0.602 | 0.603 | 0.603 |

Permutation test for rda under reduced model:

|  | Df | Variance | F | Pr(>F) |  |
| --- | --- | --- | --- | --- | --- |
| Model | 10 | 6.0316 | 1.8239 | 0.064 | . |
| Residual | 12 | 3.9684 |  |  |  |
| *Tests for axes* | |  |  |  |  |
| RDA1 | 1 | 4.5413 | 13.7324 | 0.001 | *** |
| RDA2 | 1 | 0.8252 | 2.4952 | 0.088 | . |
| RDA3 | 1 | 0.2995 | 0.9057 | 0.414 |  |
| RDA4 | 1 | 0.2048 | 0.6192 | 0.596 |  |
| RDA5 | 1 | 0.0661 | 0.2 | 0.943 |  |
| RDA6 | 1 | 0.0456 | 0.138 | 0.98 |  |
| RDA7 | 1 | 0.0228 | 0.0689 | 0.999 |  |
| RDA8 | 1 | 0.0166 | 0.0501 | 0.999 |  |
| RDA9 | 1 | 0.0097 | 0.0294 | 1 |  |
| RDA10 | 1 | 0 | 0.0001 | 1 |  |
| Residual | 12 | 3.9684 |  |  |  |
| *Tests for variables* | |  |  |  |  |
| envPC1 | 1 | 2.5857 | 7.8189 | 0.001 | *** |
| envPC2 | 1 | 1.6768 | 5.0705 | 0.009 | ** |
| envPC3 | 1 | 0.4395 | 1.3289 | 0.232 |  |
| envPC4 | 1 | 0.2032 | 0.6145 | 0.588 |  |
| envPC5 | 1 | 0.2997 | 0.9064 | 0.369 |  |
| envPC6 | 1 | 0.1744 | 0.5273 | 0.622 |  |
| envPC7 | 1 | 0.196 | 0.5927 | 0.56 |  |
| envPC8 | 1 | 0.1647 | 0.4982 | 0.652 |  |
| envPC9 | 1 | 0.1789 | 0.541 | 0.618 |  |
| envPC10 | 1 | 0.1127 | 0.3407 | 0.793 |  |
| Residual | 12 | 3.9684 |  |  |  |

Backward selection of variables- last step:

| variable | Df | AIC | F | Pr(>F) |  |
| --- | --- | --- | --- | --- | --- |
| envPC2 | 1 | 49.056 | 5.8451 | 0.01 | ** |
| envPC1 | 1 | 51.279 | 8.4682 | 0.01 | ** |

6. Summary of results of most parsimonious model for abiotic variables –reduced dataset

Call: rda(formula = Resp~envPC1+envPC2, scale=TRUE))

Partitioning of correlations:

|  | Inertia | Proportion | Adjusted R^2^ |
| --- | --- | --- | --- |
| Total | 10 | 1 |  |
| Constrained | 4.263 | 0.4263 | 0.368877 |
| Unconstrained | 5.737 | 0.5737 |  |

Eigenvalues and their contribution to the correlations

Importance of components:

|  | RDA1 | RDA2 |
| --- | --- | --- |
| Eigenvalue | 3.86 | 0.403 |
| Proportion explained | 0.386 | 0.0403 |
| Cumulative proportion | 0.386 | 0.4263 |

Permutation test for rda under reduced model:

|  | Df | Var | F | Pr(>F) |  |
| --- | --- | --- | --- | --- | --- |
| Model | 2 | 4.2625 | 7.4293 | 0.001 | *** |
| Residual | 20 | 5.7375 |  |  |  |
| *Tests for axes* | |  |  |  |  |
| RDA1 | 1 | 3.8595 | 13.4537 | 0.001 | *** |
| RDA2 | 1 | 0.403 | 1.4049 | 0.232 |  |
| Residual | 20 | 5.7375 |  |  |  |
| *Tests for variables* | |  |  |  |  |
| envPC1 | 1 | 2.5857 | 9.0134 | 0.002 | ** |
| envPC2 | 1 | 1.6768 | 5.8451 | 0.007 | ** |
| Residual | 20 | 5.7375 |  |  |  |

7. Summary of Initial model with all biotic environmental variables – reduced dataset

Call: rda(formula = Resp~%Pung+trout+pungitius+gyro+schisto+Trout_catch_rate, scale=TRUE)

Partitioning of correlations:

|  | Inertia | Proportion | Adjusted R^2^ |
| --- | --- | --- | --- |
| Total | 10 | 1 |  |
| Constrained | 6.71 | 0.671 | 0.547659 |
| Unconstrained | 3.29 | 0.329 |  |

Eigenvalues and their contribution to the correlations

Importance of components:

|  | RDA1 | RDA2 | RDA3 | RDA4 | RDA5 | RDA6 |
| --- | --- | --- | --- | --- | --- | --- |
| Eigenvalue | 5.3977 | 0.71509 | 0.46675 | 0.08279 | 0.03348 | 0.01448 |
| Proportion explained | 0.5398 | 0.07151 | 0.04667 | 0.00828 | 0.00335 | 0.00145 |
| Cumulative proportion | 0.5398 | 0.61127 | 0.65795 | 0.66623 | 0.66958 | 0.67102 |

Permutation test for rda under reduced model:

|  | Df | Variance | F | Pr(>F) |  |
| --- | --- | --- | --- | --- | --- |
| Model | 6 | 6.7102 | 5.4393 | 0.001 | *** |
| Residual | 16 | 3.2898 |  |  |  |
| *Tests for axes* | |  |  |  |  |
| RDA1 | 1 | 5.3977 | 26.2519 | 0.001 | *** |
| RDA2 | 1 | 0.7151 | 3.4779 | 0.016 | * |
| RDA3 | 1 | 0.4667 | 2.2701 | 0.063 | . |
| RDA4 | 1 | 0.0828 | 0.4026 | 0.77 |  |
| RDA5 | 1 | 0.0335 | 0.1628 | 0.978 |  |
| RDA6 | 1 | 0.0145 | 0.0704 | 0.999 |  |
| Residual | 16 | 3.2898 |  |  |  |
| *Tests for variables* | |  |  |  |  |
| %Pung | 1 | 1.82 | 8.8518 | 0.002 | ** |
| Trout_presence | 1 | 1.7199 | 8.3651 | 0.004 | ** |
| Pungitius_presence | 1 | 0.2925 | 1.4224 | 0.219 |  |
| gyro | 1 | 0.8736 | 4.2486 | 0.021 | * |
| schisto | 1 | 0.2039 | 0.9915 | 0.354 |  |
| Trout_catch_rate | 1 | 1.8004 | 8.7565 | 0.002 | ** |
| Residual | 16 | 3.2898 |  |  |  |

| variable | Df | AIC | F | Pr(>F) |  |
| --- | --- | --- | --- | --- | --- |
| Trout_presence | 1 | 41.778 | 3.2809 | 0.045 | * |
| Pungitius_presence | 1 | 44.589 | 6.1771 | 0.01 | ** |
| Trout_catch_rate | 1 | 51.92 | 15.6282 | 0.005 | ** |

8. Summary of results of most parsimonious model for biotic variables - reduced dataset

Call: rda(formula = Resp~trout+pungitius+Trout_catch_rate, scale=TRUE)

Partitioning of correlations:

|  | Inertia | Proportion | Adjusted R^2^ |
| --- | --- | --- | --- |
| Total | 10 | 1 |  |
| Constrained | 5.776 | 0.5776 | 0.510928 |
| Unconstrained | 4.224 | 0.4224 |  |

Eigenvalues and their contribution to the correlations

Importance of components:

|  | RDA1 | RDA2 | RDA3 |
| --- | --- | --- | --- |
| Eigenvalue | 4.6922 | 0.68536 | 0.39863 |
| Proportion explained | 0.4692 | 0.06854 | 0.03986 |
| Cumulative proportion | 0.4692 | 0.53776 | 0.57762 |

Permutation test for rda under reduced model:

|  | Df | Var | F | Pr(>F) |  |
| --- | --- | --- | --- | --- | --- |
| Model | 3 | 5.7762 | 8.6611 | 0.001 | *** |
| Residual | 19 | 4.2238 |  |  |  |
| *Tests for axes* | |  |  |  |  |
| RDA1 | 1 | 4.6922 | 21.107 | 0.001 | *** |
| RDA2 | 1 | 0.6854 | 3.083 | 0.026 | * |
| RDA3 | 1 | 0.3986 | 1.7932 | 0.138 |  |
| Residual | 19 | 4.2238 |  |  |  |
| *Tests for variables* | |  |  |  |  |
| Trout_presence | 1 | 1.2933 | 5.8175 | 0.012 | * |
| Pungitius_presence | 1 | 1.0087 | 4.5374 | 0.026 | * |
| Trout_catch_rate | 1 | 3.4742 | 15.6282 | 0.001 | *** |
| Residual | 19 | 4.2238 |  |  |  |

9. Summary of results of most parsimonious model for biotic + abiotic variables

rda(formula = Resp ~ envPC1 + envPC2 + %Pung + trout, scale = TRUE)

Partitioning of correlations:

|  | Inertia | Proportion | Adjusted R^2^ |
| --- | --- | --- | --- |
| Total | 10 | 1 |  |
| Constrained | 5.497 | 0.550 | 0.468 |
| Unconstrained | 4.503 | 0.450 |  |

Eigenvalues and their contribution to the correlations

Importance of components:

|  | RDA1 | RDA2 | RDA3 | RDA4 |
| --- | --- | --- | --- | --- |
| Eigenvalue | 4.1168 | 0.68374 | 0.47366 | 0.22276 |
| Proportion explained | 0.4117 | 0.06837 | 0.04737 | 0.02228 |
| Cumulative proportion | 0.4117 | 0.48005 | 0.52742 | 0.5497 |

Permutation test for rda under reduced model:

|  | Df | Var | F | Pr(>F) |  |
| --- | --- | --- | --- | --- | --- |
| Model | 4 | 5.497 | 6.714 | 0.005 | ** |
| Residual | 22 | 4.503 |  |  |  |
| *Tests for axes* | |  |  |  |  |
| RDA1 | 1 | 4.1168 | 20.1129 | 0.005 | ** |
| RDA2 | 1 | 0.6837 | 3.3405 | 0.02714 | * |
| RDA3 | 1 | 0.4737 | 2.3141 | 0.07667 | . |
| RDA4 | 1 | 0.2228 | 1.0883 | 0.36 |  |
| Residual | 22 | 4.503 |  |  |  |
| *Tests for variables* | |  |  |  |  |
| envPC1 | 1 | 2.1932 | 10.715 | 0.01 | ** |
| envPC2 | 1 | 1.8609 | 9.0916 | 0.01 | ** |
| %Pung | 1 | 0.8557 | 4.1805 | 0.02 | * |
| Trout_presence | 1 | 0.5872 | 2.8688 | 0.07 | . |
| Residual | 22 | 4.503 |  |  |  |

10. Summary of results of most parsimonious model for abiotic +biotic variables - reduced dataset

Call: rda(formula = Resp ~ envPC1 + envPC2 + Trout + Pungitius + Trout_catch_rate, scale = TRUE)

Partitioning of correlations:

|  | Inertia | Proportion | Adjusted R^2^ |
| --- | --- | --- | --- |
| Total | 10 | 1 |  |
| Constrained | 6.865 | 0.6865 | 0.5943 |
| Unconstrained | 3.135 | 0.3135 |  |

Eigenvalues and their contribution to the correlations

Importance of components:

|  | RDA1 | RDA2 | RDA3 | RDA4 | RDA5 |
| --- | --- | --- | --- | --- | --- |
| Eigenvalue | 5.3309 | 0.76866 | 0.46448 | 0.22669 | 0.07436 |
| Proportion explained | 0.5331 | 0.07687 | 0.04645 | 0.02267 | 0.00744 |
| Cumulative proportion | 0.5331 | 0.60995 | 0.6564 | 0.67907 | 0.6865 |

Permutation test for RDA under reduced model:

|  | Df | Var | F | Pr(>F) |  |
| --- | --- | --- | --- | --- | --- |
| Model | 5 | 6.865 | 7.4455 | 0.001 | *** |
| Residual | 17 | 3.135 |  |  |  |
| *Tests for axes* | |  |  |  |  |
| RDA1 | 1 | 5.3309 | 28.9078 | 0.001 | *** |
| RDA2 | 1 | 0.7687 | 4.1682 | 0.008 | ** |
| RDA3 | 1 | 0.4645 | 2.5188 | 0.043 | * |
| RDA4 | 1 | 0.2267 | 1.2293 | 0.283 |  |
| RDA5 | 1 | 0.0744 | 0.4033 | 0.834 |  |
| Residual | 17 | 3.135 |  |  |  |
| *Tests for variables* | |  |  |  |  |
| envPC1 | 1 | 2.58571 | 14.0216 | 0.001 | *** |
| envPC2 | 1 | 1.67681 | 9.0929 | 0.002 | ** |
| Trout_presence | 1 | 0.55019 | 2.9835 | 0.056 | . |
| Pungitius_presence | 1 | 0.53847 | 2.92 | 0.06 | . |
| Trout_catch_rate | 1 | 1.51388 | 8.2093 | 0.002 | ** |
| Residual | 17 | 3.13495 |  |  |  |

11. Summary of initial model with genetic variables

Call: rda(formula = Resp ~ genPC1 + genPC2 + genPC3 + genPC4 + genPC5 + genPC6 + genPC7 + genPC8 + genPC9 + genPC10, scale = TRUE)

Partitioning of correlations:

|  | Inertia | Proportion | Adjusted R^2^ |
| --- | --- | --- | --- |
| Total | 10 | 1 |  |
| Constrained | 7.063 | 0.7063 | 0.2867 |
| Unconstrained | 2.937 | 0.2937 |  |

Eigenvalues and their contribution to the correlations

Importance of components:

|  | RDA1 | RDA2 | RDA3 | RDA4 | RDA5 | RDA6 | RDA7 | RDA8 | RDA9 | RDA10 |
| --- | --- | --- | --- | --- | --- | --- | --- | --- | --- | --- |
| Eigenvalue | 5.313 | 0.822 | 0.5129 | 0.2345 | 0.0952 | 0.0488 | 0.022 | 0.0087 | 0.0036 | 0.00042 |
| Proportion explained | 0.752 | 0.116 | 0.0726 | 0.0332 | 0.0134 | 0.0069 | 0.003 | 0.0012 | 0.0005 | 0.00006 |
| Cumulative proportion | 0.752 | 0.868 | 0.9413 | 0.9745 | 0.9880 | 0.9949 | 0.998 | 0.9994 | 0.9999 | 1 |

Permutation test for RDA under reduced model:

|  | Df | Var | F | Pr(>F) |  |
| --- | --- | --- | --- | --- | --- |
| Model | 10 | 7.063 | 1.6834 | 0.125 | n.s. |
| Residual | 7 | 2.937 |  |  |  |
| *Tests for axes* | |  |  |  |  |
| RDA1 | 1 | 5.3133 | 12.6638 | 0.005 | ** |
| RDA2 | 1 | 0.8227 | 1.9609 | 0.178 |  |
| RDA3 | 1 | 0.5129 | 1.2225 | 0.325 |  |
| RDA4 | 1 | 0.2346 | 0.5591 | 0.594 |  |
| RDA5 | 1 | 0.0952 | 0.2269 | 0.861 |  |
| RDA6 | 1 | 0.0489 | 0.1165 | 0.962 |  |
| RDA7 | 1 | 0.0226 | 0.0539 | 0.985 |  |
| RDA8 | 1 | 0.0087 | 0.0208 | 1 |  |
| RDA9 | 1 | 0.0037 | 0.0088 | 1 |  |
| RDA10 | 1 | 0.0004 | 0.001 | 1 |  |
| Residual | 7 | 2.937 |  |  |  |
| *Tests for variables* | |  |  |  |  |
| genPC1 | 1 | 1.41713 | 3.3776 | 0.035 | * |
| genPC2 | 1 | 0.78894 | 1.8804 | 0.141 |  |
| genPC3 | 1 | 0.66891 | 1.5943 | 0.209 |  |
| genPC4 | 1 | 0.5889 | 1.4036 | 0.233 |  |
| genPC5 | 1 | 1.00286 | 2.3902 | 0.091 | . |
| genPC6 | 1 | 0.90353 | 2.1535 | 0.104 |  |
| genPC7 | 1 | 0.4808 | 1.1459 | 0.324 |  |
| genPC8 | 1 | 0.20451 | 0.4874 | 0.683 |  |
| genPC9 | 1 | 0.15881 | 0.3785 | 0.791 |  |
| genPC10 | 1 | 0.84864 | 2.0226 | 0.152 |  |
| Residual | 7 | 2.93698 |  |  |  |

12. Summary of results of most parsimonious model for genetic variables

Call: rda(formula = Resp ~ genPC1, scale = TRUE)

Partitioning of correlations:

|  | Inertia | Proportion | Adjusted R^2^ |
| --- | --- | --- | --- |
| Total | 10 | 1 |  |
| Constrained | 1.417 | 0.1417 | 0.08807 |
| Unconstrained | 8.583 | 0.8583 |  |

Eigenvalues and their contribution to the correlations

Importance of components:

|  | RDA1 |
| --- | --- |
| Eigenvalue | 1.417 |
| Proportion explained | 1 |
| Cumulative proportion | 1 |

Permutation test for rda under reduced model:

|  | Df | Var | F | Pr(>F) |  |
| --- | --- | --- | --- | --- | --- |
| Model | 1 | 1.4171 | 2.6418 | 0.08 | . |
| Residual | 16 | 8.5829 |  |  |  |
| *Tests for axes* | |  |  |  |  |
| RDA1 | 1 | 1.4171 | 2.6418 | 0.084 | . |
| Residual | 16 | 8.5829 |  |  |  |
|  |  |  |  |  |  |
| *Tests for variables* | |  |  |  |  |
| genPC1 | 1 | 1.4171 | 2.6418 | 0.07 | . |
| Residual | 16 | 8.5829 |  |  |  |
